# Single-cell RNA-Seq of human esophageal epithelium in homeostasis and allergic inflammation

**DOI:** 10.1172/jci.insight.159093

**Published:** 2022-06-08

**Authors:** Mark Rochman, Ting Wen, Michael Kotliar, Phillip J. Dexheimer, Netali Ben-Baruch Morgenstern, Julie M. Caldwell, Hee-Woong Lim, Marc E. Rothenberg

**Affiliations:** 1 Division of Allergy and Immunology, Cincinnati Children’s Hospital Medical Center (CCHMC), Cincinnati, Ohio, USA.; 2 Department of Pediatrics, CCHMC and the University of Cincinnati College of Medicine, Cincinnati, Ohio, USA.; 3 Division of Biomedical Informatics, CCHMC, Cincinnati, Ohio, USA.

**Keywords:** Immunology, Inflammation, Cellular immune response, Molecular pathology

## Abstract

Inflammation of the esophageal epithelium is a hallmark of eosinophilic esophagitis (EoE), an emerging chronic allergic disease. Herein, we probed human esophageal epithelial cells at single-cell resolution during homeostasis and EoE. During allergic inflammation, the epithelial differentiation program was blocked, leading to loss of *KRT6^hi^* differentiated populations and expansion of *TOP2^hi^* proliferating, *DSP^hi^* transitioning, and *SERPINB3^hi^* transitioning populations; however, there was stability of the stem cell–enriched PDPN^hi^ basal epithelial compartment. This differentiation program blockade was associated with dysregulation of transcription factors, including nuclear receptor signalers, in the most differentiated epithelial cells and altered NOTCH-related cell-to-cell communication. Each epithelial population expressed genes with allergic disease risk variants, supporting their functional interplay. The esophageal epithelium differed notably between EoE in histologic remission and controls, indicating that remission is a transitory state poised to relapse. Collectively, our data uncover the dynamic nature of the inflamed human esophageal epithelium and provide a framework to better understand esophageal health and disease.

## Graphical abstract

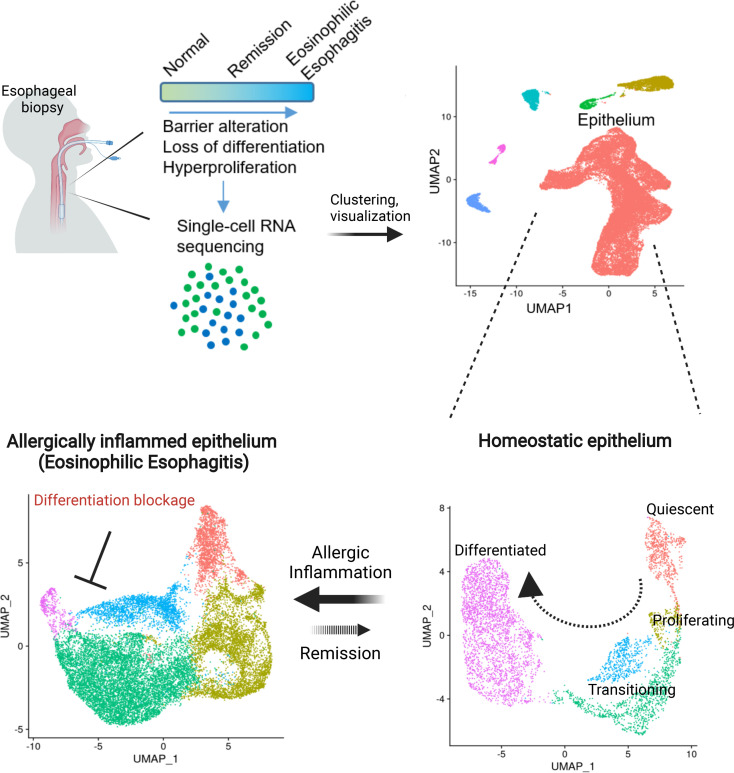

## Introduction

The esophagus has been traditionally thought of as a relatively simple organ that primarily functions as a conduit for food passage from the oral cavity to the stomach. However, accumulating data are changing this view with the emergence of the esophagus as a complex mucosa with a multilayered epithelium designed to maintain homeostasis in the setting of the constant onslaught of antigenic and biophysical stimuli (1, 2). To achieve this, the human esophagus is lined with a multilayered, squamous, nonkeratinized epithelium that forms a protective barrier against extrinsic (e.g., bacteria and food antigens) and intrinsic (e.g., acid) stimuli.

The epithelial cells of the protective mucosal barrier of the human esophagus are primarily defined by their shape, proliferation status, and spatial localization. The layer of the human esophageal epithelium furthest from the lumen (the basal epithelium) consists of mostly quiescent cells and serves as a reservoir of the epithelial progenitors defined by COL17A1/KRT15^hi^ expression (1, 3). Adjacent to the basal layer and closer to the lumen are a few layers of actively proliferating cells. As the epithelial cells move toward the luminal surface, they embark in the differentiation process and become progressively flattened, form a tighter barrier, and lose their nuclei. Differentiated epithelial cells are characterized by the high expression of KRT4, KRT13, and other esophagus-enriched proteins (4). The molecular properties and functional relationship between human epithelial subpopulations, developmental trajectory of the epithelium, and interepithelial communications under homeostasis and allergic inflammation are at an early stage of characterization (1).

Several diseases of the esophagus are characterized by profound epithelial responses, including gastroesophageal reflux disease (GERD), squamous cell carcinoma, and allergic eosinophilic esophagitis (EoE). In EoE, RNA-Seq performed on whole tissue specimens has identified numerous pathways involved in immunity and epithelial biology (5, 6), but it has not revealed the contribution of distinct epithelial subpopulations. Therefore, the detailed changes in the epithelial differentiation program and the origin of basal zone hyperplasia, seen in many esophageal inflammatory diseases, remain unexplored. At the molecular level, genes genetically linked to food allergy, and specifically EoE, are often expressed in the esophagus (7, 8); however, it remains to be determined which specific cell types, if any, are responsible for the disease susceptibility and pathology. To address these gaps in knowledge, we performed single-cell RNA-Seq (scRNA-Seq) of the esophageal biopsies from a spectrum of individuals, including healthy controls and individuals with active and inactive stages of EoE as identified by histologic criteria, and we focused on the epithelial compartment of the esophageal tissue, defined herein as the esophageal epithelial unit (EEU). Collectively, our data highlight previously unappreciated heterogeneity of the homeostatic and inflamed esophageal epithelium and provide a framework to better understand esophageal health and disease.

## Results

### Esophageal tissue cellular composition in the homeostatic and inflamed esophagus.

**Figure 1:**
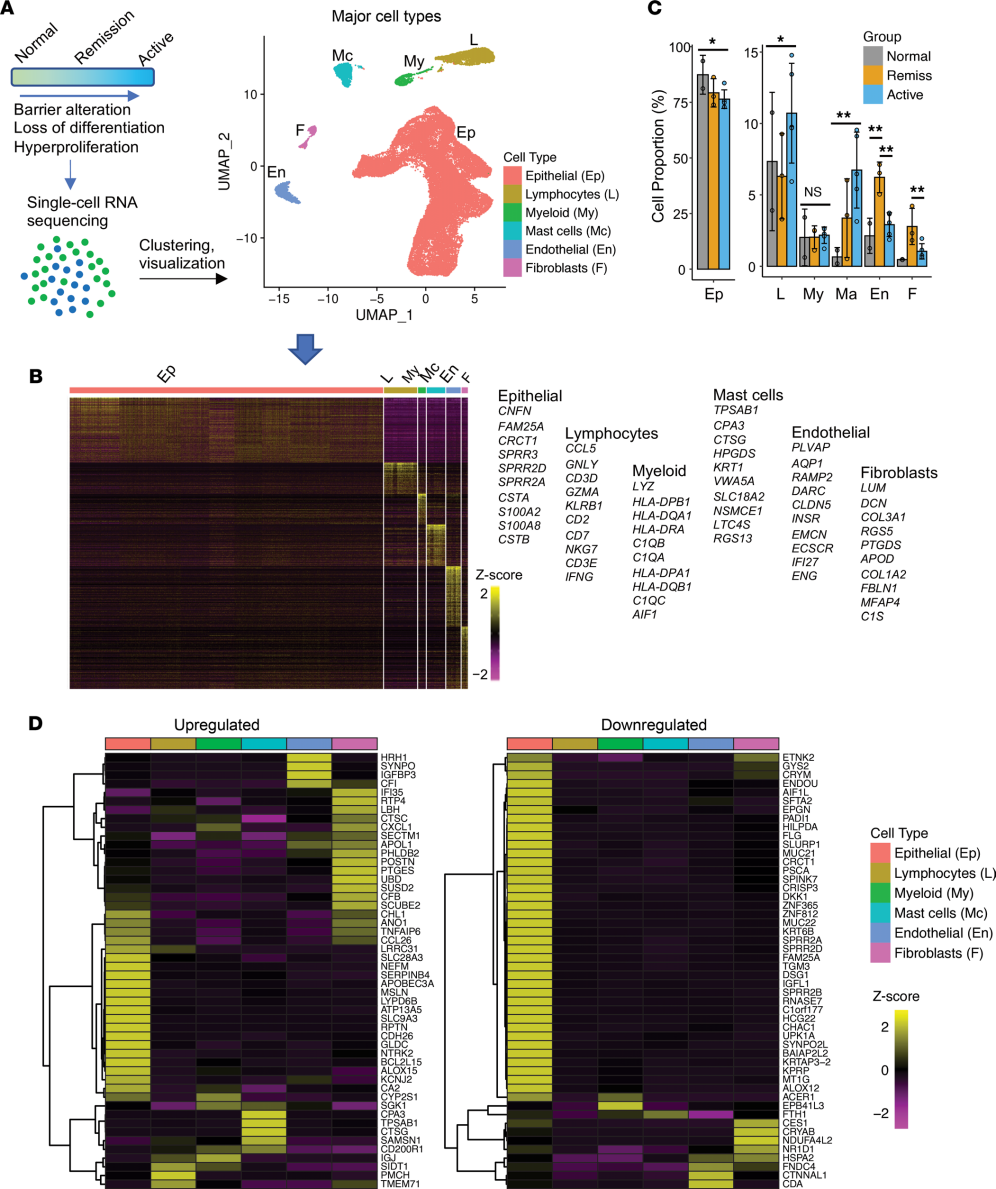
Single-cell analysis of human esophageal biopsies delineating major esophageal cell types. (**A**) Schematic representation of the experimental design and the uniform manifold approximation and projection (UMAP) of all samples depicting major cell types from the esophageal biopsies. *n* = 2, healthy controls (normal); *n* = 3, EoE in remission (remission); *n* = 5, active EoE inflammation (active). (**B**) Marker gene expression heatmap of major cell types and top 10 representative genes per cell type. Each column represents a single cell, and each row represents an individual gene. (**C**) Quantification of the cell types and comparison of their proportion by disease status. *P = 0.06; **P < 0.05; 2-tailed Student’s *t* test. Data are shown as mean ± SD. (**D**) Heatmap of average expression of all 10 samples for top 50 upregulated and downregulated genes from the bulk RNA-Seq EoE transcriptome (6) stratified by the cell type.

To systematically examine the cellular and transcriptional changes in the homeostatic and inflamed esophagus, we performed scRNA-Seq of 40,297 cells derived from esophageal biopsies (*n* = 5 individuals with active EoE, *n* = 3 individuals with EoE in disease remission, *n* = 2 healthy controls) (Figure 1A). To define the major cellular repertoire that was comparable across all samples, we integrated all samples, while retaining sample and disease status information for each cell, and performed unbiased clustering and Uniform Manifold Approximation Projection (UMAP) (9) by Seurat (10). It revealed 6 major cell types, with epithelial cells being the most prevalent in all conditions (82% on average; Figure 1, B and C). Representative markers of the major subpopulations included *CNFN* and *SPRRs* (epithelial cells), *CD3D* (lymphocytes), *HLA* (myeloid cells), *TPSAB1* and *CPA3* (mast cells), *AQP1* and *CLDN5* (endothelial cells), and *COL3A1* (fibroblasts). Marker genes representative for each cell type and gene ontology (GO) analysis confirmed the cell identities (Figure 1B, Supplemental Figure 1, and Supplemental Table 1; supplemental material available online with this article; https://doi.org/10.1172/jci.insight.159093DS1). The percentages of lymphocytes, mast cells, endothelial cells, and fibroblasts were increased, whereas the percentage of epithelial cells was moderately decreased in active EoE compared with healthy controls (Figure 1C). Notably, the cellular composition in EoE remission displayed a higher percentage of mast cells, endothelial cells, and fibroblasts and a modestly decreased the percentage of epithelial cells compared with that of healthy controls (Figure 1C). Analysis of the expression of the genes most highly dysregulated in the EoE transcriptome defined by bulk tissue RNA-Seq (6) revealed the contribution of each cell type to the transcriptional response in the disease (Figure 1D). Notably, esophageal epithelial cells and fibroblasts upregulated several genes that are critical to EoE pathogenesis, including *CDH26*, *POSTN*, *ANO1*, and *CCL26*; the latter 2 were shared between these cell types (Figure 1D, upregulated). In contrast, downregulated genes were primarily expressed in the epithelial component of the esophagus (Figure 1D, downregulated). These findings collectively signify a key role of epithelial responses and their unique cellular trajectory in the inflamed esophagus.

### EEU in the homeostatic esophagus.

**Figure 2:**
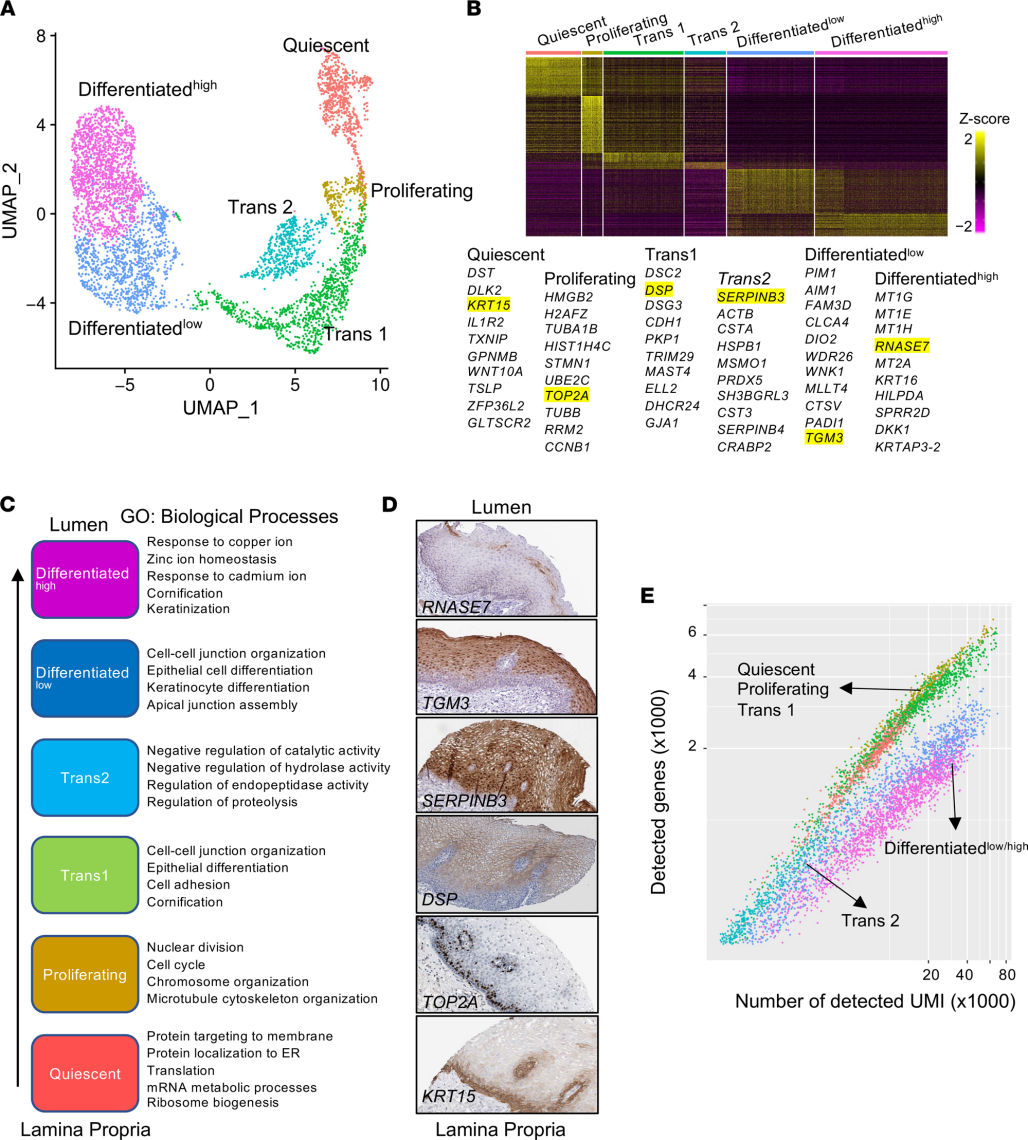
scRNA-Seq analysis of homeostatic (normal) esophageal epithelium. (**A**) UMAP projection depicting 6 esophageal epithelial subpopulations. (**B**) Marker gene expression heatmap and top 10 representative genes of epithelial subpopulations. Each column represents a single cell, and each row represents an individual gene. Genes highlighted in yellow are accompanied by IHC images in **D**. (**C**) The spatial organization of the epithelial subpopulations in the esophageal epithelium. Arrow indicates the direction of the putative differentiation program from the basal cells adjacent to the lamina propria toward the lumen. Most significant biological process GO terms are shown for each subpopulation. (**D**) IHC images of the representative markers (yellow highlighting in **B**) are screenshots from the Human Protein Atlas (HPA) for each subpopulation. See the HPA for magnification of original images (4). (**E**) Scatter plot of UMI counts versus number of detected genes per cell showing relatively smaller number of expressed genes in the differentiated subpopulations. Color coding is the same as in **A**.

We sought to explore the heterogeneity of the esophageal epithelium under homeostatic states. Unbiased clustering of the epithelial compartment from healthy individuals revealed 6 subpopulations of the EEU (representative markers are shown in parentheses): Quiescent (*DST*, *KRT15*), Proliferating (*HMGB2*, *TOP2A*), Transitioning 1 (Trans1; *DSC2*, *DSP*), Trans2 (*SERPINB3*), Differentiated^lo^ (*PADI1*, *TGM3*), and Differentiated^hi^ (*MT1G*, *RNASE7*, *KRT16*) (Figure 2, A and B, and Supplemental Table 2). Consistent with distinct transcriptional signatures, each epithelial subpopulation was characterized by unique biological processes reflecting functional diversity (Figure 2C, biological processes), substantiating that they are discrete subpopulations with a stable presence and defined function. Quiescent cells were highly enriched in genes related to membrane protein targeting and translation; proliferating cells were associated with the cell division genes; and transitioning and differentiated subpopulations were enriched in genes associated with epithelial differentiation, cell junctional complexes, adhesion, and cornification. Notably, the Trans2 subpopulation specifically expressed the esophagus-enriched serine protease inhibitor genes *SERPINB3* and *SERPINB4*, cystatin family genes *CSTA* and *CSTB*, and the peptidase inhibitor 3 gene (*PI3*); it was uniquely enriched in endopeptidase activity, suggesting that the Trans2 subpopulation functions as a primary regulator of the proteolytic activity germane to the protective properties of the esophageal epithelium under homeostatic conditions (7). The Differentiated^hi^ subpopulation was enriched in genes related to response to ions, including copper, zinc, and cadmium (metallothioneins), and had specific expression of the antimicrobial gene *RNASE7* (Figure 2B and Supplemental Figure 2). Immunostaining of the representative markers of each subpopulation revealed that basal cell markers (e.g., *KRT15*) were most strongly expressed in the basal layer of the esophagus, proliferating cell markers (e.g., *TOP2A*) were expressed in the layers adjacent to the basal layer but not the lamina propria, and the transitioning and differentiated cell markers (e.g., *DSP*, *SERPINB3*, *TGM3*, *RNASE7*) were primarily expressed in the suprabasal zone (Figure 2D). When comparing the number of detected genes and the read depth (number of unique molecular identifier [UMI]) across the distinct epithelial subpopulations, we observed that Trans2 and differentiated cells expressed a lower number of genes than did other epithelial subpopulations, suggesting that these cells were highly specialized in function (Figure 2E). These results demonstrate that the differentiation program of the esophageal epithelium mirrors epithelial stratification; less differentiated and proliferating cells reside closer to the lamina propria, and the most differentiated cells reside adjacent to the lumen.

### Basal and suprabasal epithelial layer properties.

**Figure 3:**
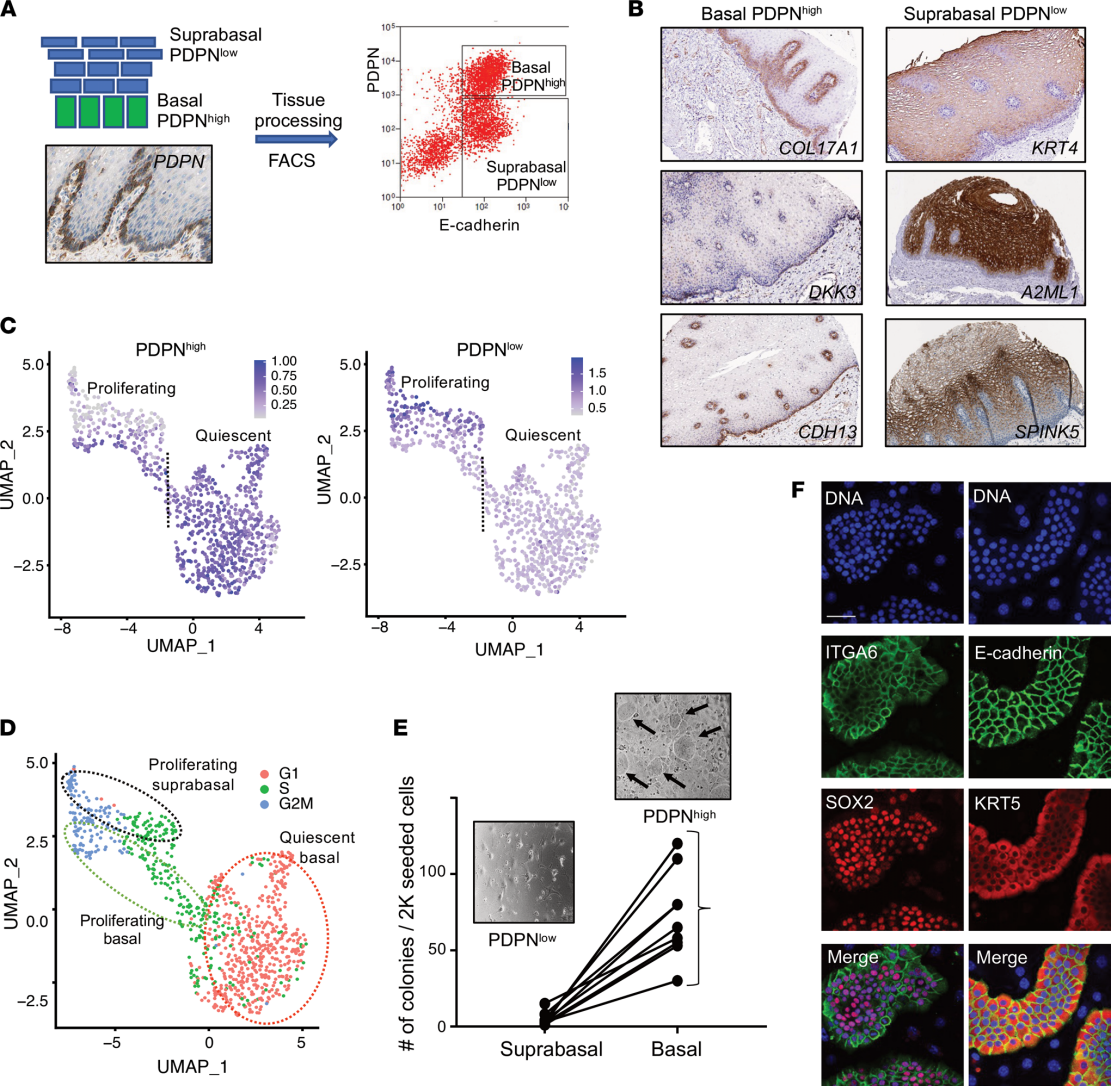
Functional and molecular properties of the human esophageal basal layer cells in homeostasis. (**A**) Schematic representation of the experimental design to purify and characterize basal layer cells (upper left). Podoplanin (PDPN) staining depicting basal layer localization from the Human Protein Atlas (HPA) (lower left). A representative FACS plot shows the gating strategy to purify PDPN^hi^ for basal cells and PDPN^lo^ for suprabasal cells (right). (**B**) IHC images are screenshots from HPA of the representative markers for basal and suprabasal cells identified by differential analysis of bulk RNA-Seq data comparing PDPN^hi^ and PDPN^lo^ cells; see the HPA for magnification of original images (4). (**C**) Average expression of top 20 PDPN^hi^ and PDPN^lo^ marker genes projected onto UMAP of quiescent and proliferating cells from healthy controls distinguishing basal and suprabasal cells; the dotted line separates quiescent and proliferating cells. (**D**) Cell cycle status of cells inferred by Seurat projected onto UMAP of quiescent and proliferating cells. (**E**) Ex vivo colony-forming assay for sorted basal and suprabasal cells. Each line represents cells from a biopsy obtained from a distinct individual. A total number of colonies per 2000 seeded cells are plotted. Representative images of the high-power microscopic field show colony growth of basal PDPN^hi^ cells (arrows; upper image, ×10 magnification). Cells in the PDPN^lo^ image are mitotically inactivated murine fibroblasts that served as feeder layer cells for colony growth. (**F**) Immunofluorescence images of the colonies grown from the PDPN^hi^ cells. Scale bar: 50 μM.

To further delineate early events in the differentiation of the esophageal epithelium, we aimed to molecularly characterize the basal epithelial layer of the esophagus adjacent to the lamina propria. Accordingly, we sorted biopsy-derived epithelial cells by their expression of the basal layer marker podoplanin (PDPN) (11) and defined the gene signature of the basal (PDPN^hi^) and the suprabasal (PDPN^lo^) compartments by bulk RNA-Seq (Figure 3A and Supplemental Tables 3 and 4). Differential expression analysis identified 151 genes specific to basal (PDPN^hi^) cells, including *IGFBP3*, *COL17A1*, and *KRT15* (reads per kilobase of transcript, per million mapped reads [RPKM] > 5, fold change [FC] > 2 versus suprabasal [PDPN^lo^] cells, FDR-adjusted *P* < 0.05; Supplemental Table 3). A unique set of 248 genes was expressed in the suprabasal (PDPN^lo^) cells, including *KRT4* and *KRT13*, *SPINK5*, *SERPINB13*, and most of the esophagus-enriched genes (7) (green font in Supplemental Table 4). Immunostaining of the most highly expressed markers of each cell type showed clear morphologic separation of the epithelium into basal and suprabasal compartments (Figure 3B). Functional enrichment analysis revealed that genes expressed in the basal (PDPN^hi^) cells were associated with cell adhesion, migration, and extracellular matrix processes, whereas genes expressed in the suprabasal (PDPN^lo^) cells were linked to epithelial development, differentiation, and cornification (Supplemental Figure 3A).

We sought to explore the proliferating status of the basal and the suprabasal cells. To do this, we overlaid average gene expression signatures of the PDPN^lo^ and PDPN^hi^ cells with those of quiescent and proliferating populations and with the cell cycle genes. The PDPN^hi^ signature overlaid with the quiescent and part of the proliferating population, whereas the PDPN^lo^ signature was found exclusively in the proliferating population (Figure 3C). Cell cycle analysis using Seurat revealed the presence of genes from the full spectrum of the cell cycle: G1, S, and G2M. Most of the basal (PDPN^hi^) cells were in G1 or S stage, and a small portion of basal (PDPN^hi^) cells were in the G2M stage. Suprabasal (PDPN^lo^) cells were in S and G2M stage (Figure 3D). This finding suggests continuous transition from quiescent basal to proliferating basal to proliferating suprabasal cells in the epithelial compartment.

Basal (PDPN^hi^) cells expressed markers previously associated with epithelial progenitors, including *COL17A1*, *DST*, *KRT15* (1), and specifically those with antiproliferative properties, such as *ZFP36L2* (12) and *BTG2* (13) (Supplemental Figure 3B), collectively suggesting that the basal cells are the source of the esophageal epithelial stem cells. Indeed, ex vivo colony formation revealed preferential growth of the stem cell–like colonies from the basal (PDPN^hi^) compared with suprabasal (PDPN^lo^) cells (Figure 3E, arrows); these colonies expressed the epithelial progenitor markers *ITGA6*, *KRT5,* and *SOX2* (Figure 3F). Collectively, these findings establish the hierarchical developmental trajectory of the EEU of the homeostatic esophagus, suggesting that the sequence of early events stems from the KRT15^hi^ quiescent basal progenitors that rarely divide and progress toward the KRT4^hi^/KRT13^hi^ suprabasal proliferating cells. This trajectory resembles the differentiation program of the epidermis (14). The colony-forming property of the basal (PDPN^hi^) cells revealed that these populations are the source of the esophageal epithelial progenitors.

### EEU differentiation dynamics in allergic inflammation.

**Figure 4:**
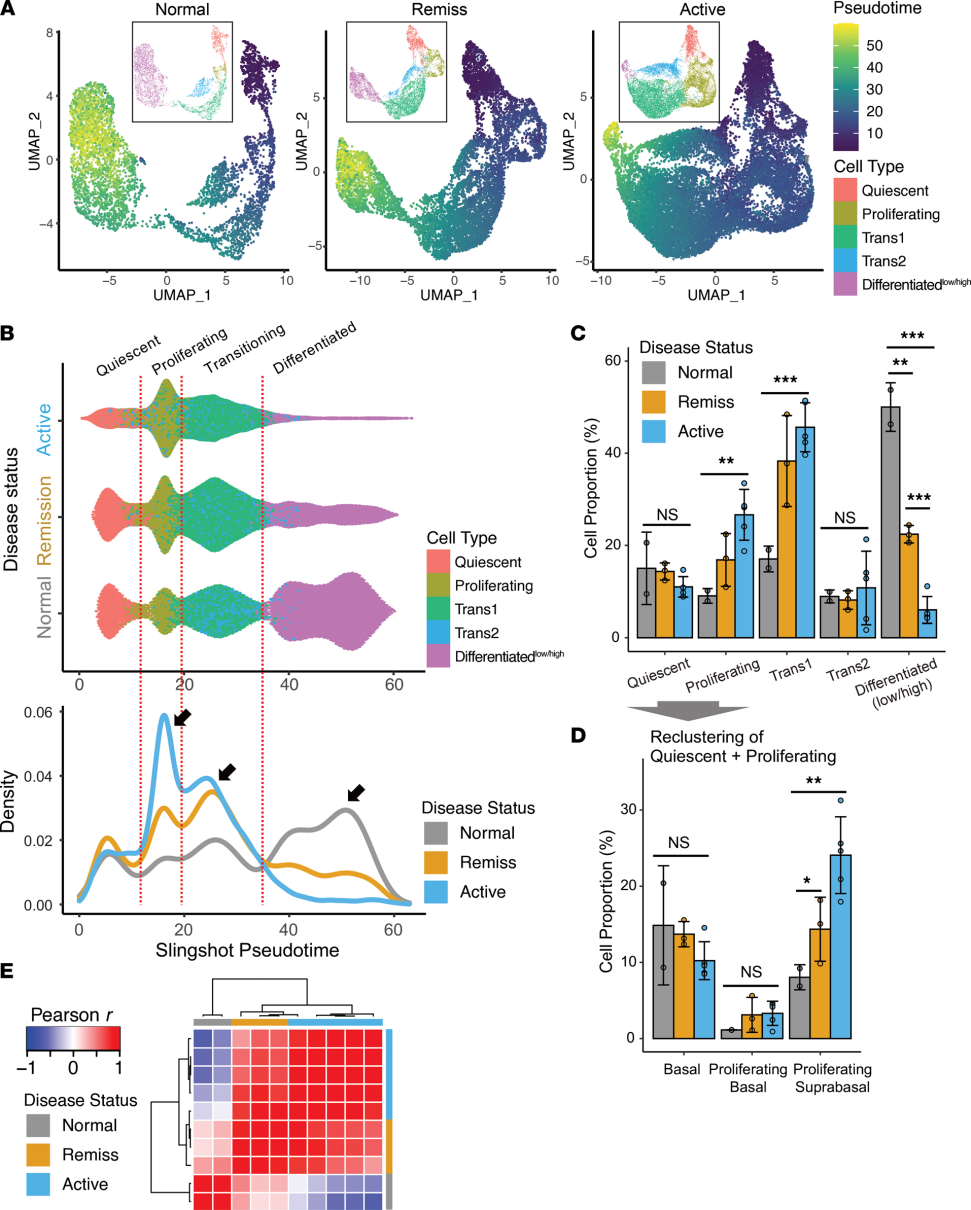
Human esophageal epithelial responses in allergic inflammation. (**A**) Slingshot pseudotime of esophageal epithelium differentiation projected onto UMAP in color scale for healthy controls (Normal), EoE remission (Remiss), and active EoE (Active). Insets show the epithelial subpopulations identified by unbiased clustering. (**B**) Swarm and density plots showing cell densities along the pseudotime. (**C** and **D**) Statistical comparison of the epithelial subpopulation composition among the disease states. *P < 0.05; **P < 0.01; ***P < 0.001 by 2-tailed Student’s *t* test. Data are shown as mean ± SD. (**E**) Hierarchical clustering of all 10 samples comparing the epithelial subpopulation composition by disease status using Pearson correlation coefficient as a similarity measure (healthy controls [Normal], EoE remission [Remiss], and active EoE [Active]). Each column represents an individual sample.

We sought to compare the EEU in individuals with active EoE to the homeostatic conditions along the differentiation trajectory. First, we performed sample integration, UMAP, and clustering analysis by grouping samples by their disease status: normal (healthy control), remission (inactive EoE), and active EoE. To compare EEU differentiation by disease status, we performed pseudotime analysis using Slingshot (15) by combining all 3 groups to project them onto a common pseudotime axis and identified a linear differentiation trajectory (Supplemental Figure 4A). Accordingly, individual cells were assigned with a pseudotime value along the unified trajectory for all samples and color-coded in the UMAP of the corresponding groups (Figure 4A) generated by separate processing of the group. Scatter plots of UMI counts versus the number of detected genes across epithelial subpopulations were generated (Supplemental Figure 4B). Despite the shared differentiation trajectory of the subepithelial units, there was dramatic loss of the fully differentiated cells and an increase of the transitional and proliferating cells (Figure 4A and Supplemental Figure 4B). To further examine EoE-dependent perturbation of differentiation, we visualized cell density along the pseudotime axis and compared the relative proportion of cells at each differentiation stage (Figure 4B). Indeed, active EoE displayed significantly increased proliferating cells and Trans1 cells, as well as a significant decrease of Differentiated^lo/hi^ subpopulations relative to normal controls (Figure 4C). The EoE in remission displayed a similar decrease of Differentiated^lo/hi^ cells, but the decrease was significantly less than that of active EoE (Figure 4C and Supplemental Figure 4B).

Focusing on the quiescent and proliferating subpopulations, we found that only proliferating suprabasal, but not basal cells, were significantly increased and that the proportion of quiescent basal cells remained unchanged (Figure 4D). This finding suggests that basal zone hyperplasia, which is commonly observed in EoE and other types of esophagitis, is primarily driven by the proliferation of the suprabasal epithelial compartment, likely by the proliferating and Trans1 subpopulations. At the same time, EoE remission also displayed a significant increase in the proportion of proliferating suprabasal cells relative to normal, but EoE remission has less of an increase than that of active EoE (Figure 4D). Accordingly, when comparing overall epithelial subpopulation composition by hierarchical clustering, EoE remission samples were more similar to active EoE samples than normal control samples (Figure 4E). These results demonstrate that the differentiation program of the EEU is dynamically regulated as a function of disease activity and that there is a profound disruption of this program in EoE, as evidenced by an increased proportion of transitioning and proliferating epithelial cells and a loss of differentiated epithelial cells. In disease remission, despite histologic improvement, these changes persist at the single-cell level, albeit with less magnitude than that of active disease.

### Altered EEU differentiation program in EoE.

**Figure 5:**
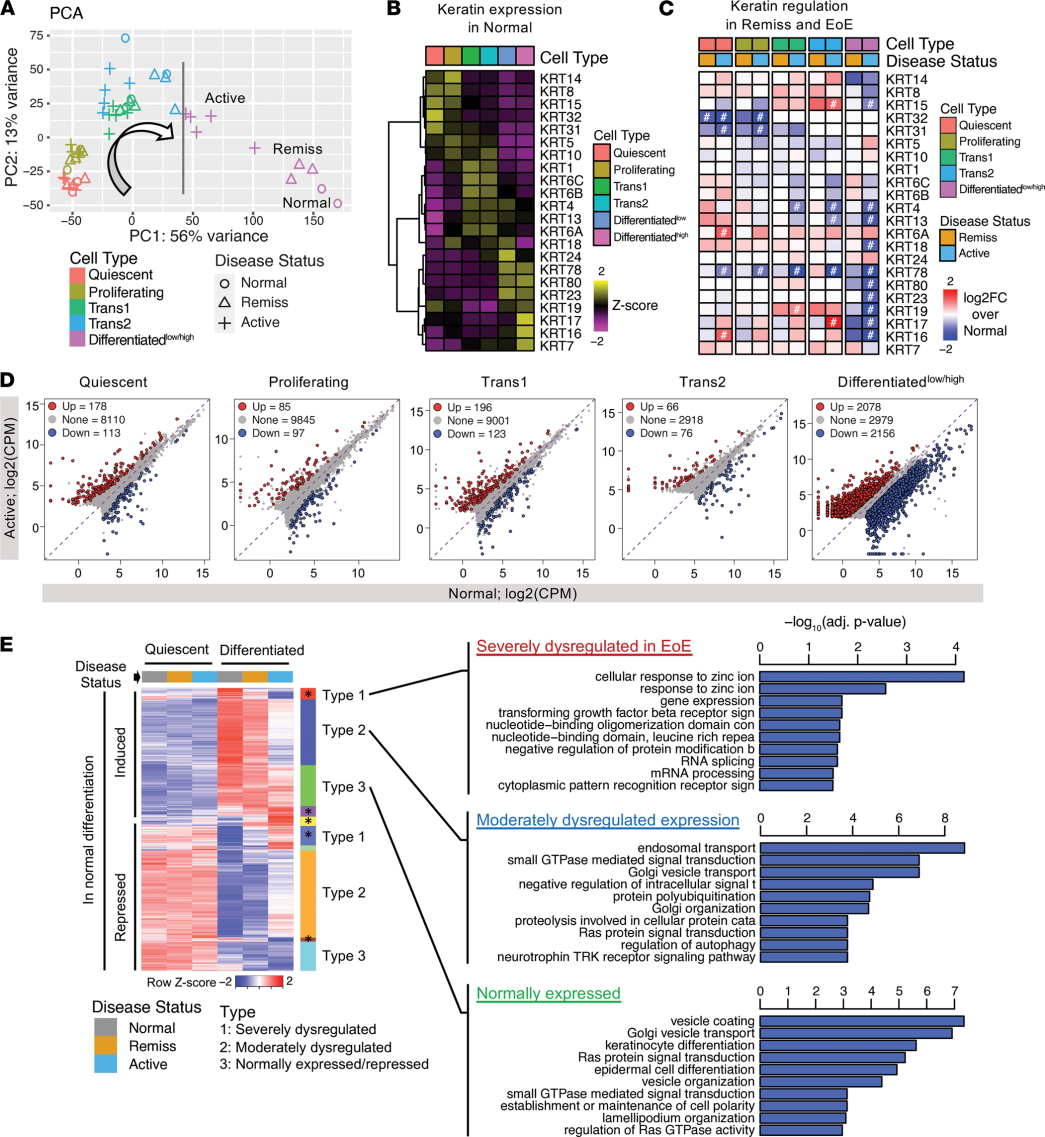
Pseudo-bulk RNA-Seq analysis to compare gene expression dysregulation and epithelial cell fate of human esophageal epithelial subpopulations by disease status. (**A**) Principal component analysis (PCA) of the pseudo-bulk RNA-Seq samples derived from scRNA-Seq by summing UMI counts per gene per epithelial subpopulation per biopsy. The arrow shows developmental trajectory; the vertical line represents developmental blockage in active EoE. (**B**) Hierarchical clustering heatmap of keratin gene expression in the healthy control epithelium (Normal). (**C**) Log_2_ fold change (log_2_FC) of keratin gene expression in active EoE (Active) and EoE remission (Remiss) samples compared with normal by the differential expression analysis of the pseudo-bulk RNA-Seq (^#^FDR-adjusted *P* < 0.05). Genes are ordered the same as in **B**. Note that the Differentiated^hi^ and Differentiated^lo^ cell subpopulations are represented by one unified cell population. (**D**) Scatter plots show differentially expressed genes in epithelial subpopulations of active EoE (EoE) compared with healthy controls (NL) (FC > 1.5 and FDR-adjusted *P* < 0.05). (**E**) Hierarchical clustering heatmap of differentially expressed genes between Differentiated and Quiescent epithelial subpopulations in the normal and diseased esophagus. Two groups of genes are observed that are either induced or repressed upon normal differentiation. In each group, induced or repressed, 3 types of patterns are observed: Type 1, severely dysregulated; Type 2, moderately dysregulated; and Type 3, normally expressed (induced or repressed). Asterisks denote genes that remain dysregulated in EoE remission. Top significantly enriched biological process GO terms are presented for the differentiation-induced genes.

Given the observation of the near-complete loss of the Differentiated^lo/hi^ subpopulations and the increased proportion of the transitioning subpopulations, we hypothesized that the EEU of the inflamed esophagus has a disrupted capacity to differentiate. To test this hypothesis, we compared gene expression of the individual epithelial subpopulations stratified by the disease status. Given that our samples are from humans and the numbers are limited, it was important to take into account sample-to-sample variability when examining the changes in EoE. Therefore, bulk RNA-Seq differential analysis was used to convert the subpopulation-stratified scRNA-Seq data into pseudo-bulk RNA-Seq data (16). Specifically, we summed UMI counts per gene for each epithelial subpopulation per sample, thereby generating a total of 50 samples (i.e., 10 samples × 5 cellular subpopulations, where Differentiated^lo/hi^ subpopulations were merged), and we then performed DESeq2 differential analysis (17). This process is analogous to FACS cell purification, followed by bulk RNA-Seq for comparative analysis of homogeneous cell populations, except that the cells are purified computationally. This analysis is independent of the increase or decrease of specific subpopulations because gene expression levels are normalized by the cell counts per sample; thus, it enables robust interrogation of transcriptomic changes in each subpopulation. First, principal component analysis (PCA) was performed to assess the similarity of the pseudo-bulk RNA-Seq samples and to examine the trajectory of each cellular compartment as a function of cellular differentiation (Figure 5A). Unlike other epithelial subpopulations, differentiated cells were widely spread along the developmental trajectory in a manner dependent upon the disease status. Differentiated cells from active EoE esophagi were located closer to the transitioning subpopulations than were differentiated cells from normal esophagi (Figure 5A), suggesting blockade of transitioning to differentiated cells (Figure 5A, line). Using keratin expression as a surrogate marker for epithelial differentiation, we first identified subpopulation-specific keratin genes (Figure 5B) and then examined their changes in EoE (Figure 5C). We observed a significant loss of keratins preferentially expressed in the normal differentiated cells, including *KRT16*, *KRT17*, *KRT80*, *KRT78*, *KRT23,* and *KRT19* (compare average expression heatmap [Figure 5B] to transcriptional change [Figure 5C] in the differentiated subpopulation in active EoE). In EoE remission, expression of keratins in the differentiated subpopulations was largely normalized (Figure 5C) except for a few that remain dysregulated as much as in active EoE. Similarly, in the PCA analysis, differentiated cells from individuals with EoE in remission clustered closer to those from healthy controls (Figure 5A). This suggests that epithelial differentiation in EoE is blocked at the terminal stage and that this blockade is partially reversed during disease remission.

We further assessed the dysregulation of the epithelial differentiation program in EoE by comparing transcriptional profiles of individual subpopulations of the EEU between active EoE and normal controls (Figure 5D). Differential analysis (FDR-adjusted *P* < 0.05, FC > 1.5) revealed that the largest number of dysregulated genes was in the Differentiated^lo/hi^ subpopulations. Differentially expressed genes (DEGs) in the differentiated cells were evenly divided between upregulated and downregulated genes (2078 genes upregulated, 2156 genes downregulated) compared with controls, suggesting that these subpopulations remain highly transcriptionally active despite their major loss in EoE. In contrast, the Trans2 subpopulation had the smallest contribution to the transcriptional response in EoE (66 genes upregulated, 76 genes downregulated). GO analysis revealed enrichment in epithelial differentiation and keratinization that were shared between quiescent, Trans2, and differentiated subpopulations for the downregulated genes. In the proliferating and Trans2 cells, downregulated genes were enriched in responses to metal ions, whereas in the differentiated cells, downregulated genes were associated with small GTPase–mediated signal transduction (Supplemental Figure 5A, downregulated). Similar analysis of the upregulated genes revealed enrichment in endopeptidase activity and type I IFN signaling that was shared between the epithelial subpopulations, and it revealed enrichment in mRNA metabolism and splicing that was specific to the differentiated subpopulations (Supplemental Figure 5A, upregulated). In EEU in EoE remission, approximately 100 genes remained dysregulated, and these were primarily in the differentiated subpopulations (Supplemental Figure 5B).

To better dissect the gene transcriptional changes in EEU differentiation programs, we performed unbiased hierarchical clustering focusing on the differential gene expression between quiescent and differentiated subpopulations (Figure 5E). We observed 2 large groups of genes that were either induced or repressed upon differentiation in the normal esophagus. Interestingly, each group of genes, induced or repressed, displayed 3 types of changes in EoE. Type 1 genes were severely dysregulated, Type 2 genes were moderately dysregulated by disease status, and Type 3 genes were normally expressed/repressed irrespective of disease status. Although some genes were normally expressed in active EoE (Type 3, compare normal and active EoE [Active]), the majority of the genes were dysregulated (Types 1 and 2), including severely dysregulated genes that failed to be either induced or repressed during differentiation compared with expression in the normal esophagus (Type 1). Focusing on the genes normally induced in differentiation, dysregulated genes (Types 1 and 2) were enriched for genes linked to endosomal transport, small GTPase signaling, and response to zinc ions. Notably, genes that were upregulated during differentiation independently of the disease status (Type 3) were functionally enriched for keratinocyte and epidermal differentiation, suggesting that these cells are actually differentiated but are functionally defective in the inflamed esophagus. In EoE remission, several clusters of genes in the differentiated cells remained dysregulated (Figure 5E, asterisks; compare EoE remission [Remiss] and normal in Differentiated cells), collectively suggesting that the cellular composition and transcriptional signature of the EEU in EoE remission represents a transition state between the healthy and inflamed esophagus that likely poises the epithelium to relapse.

### Transcription factor signature of altered epithelial differentiation.

**Figure 6:**
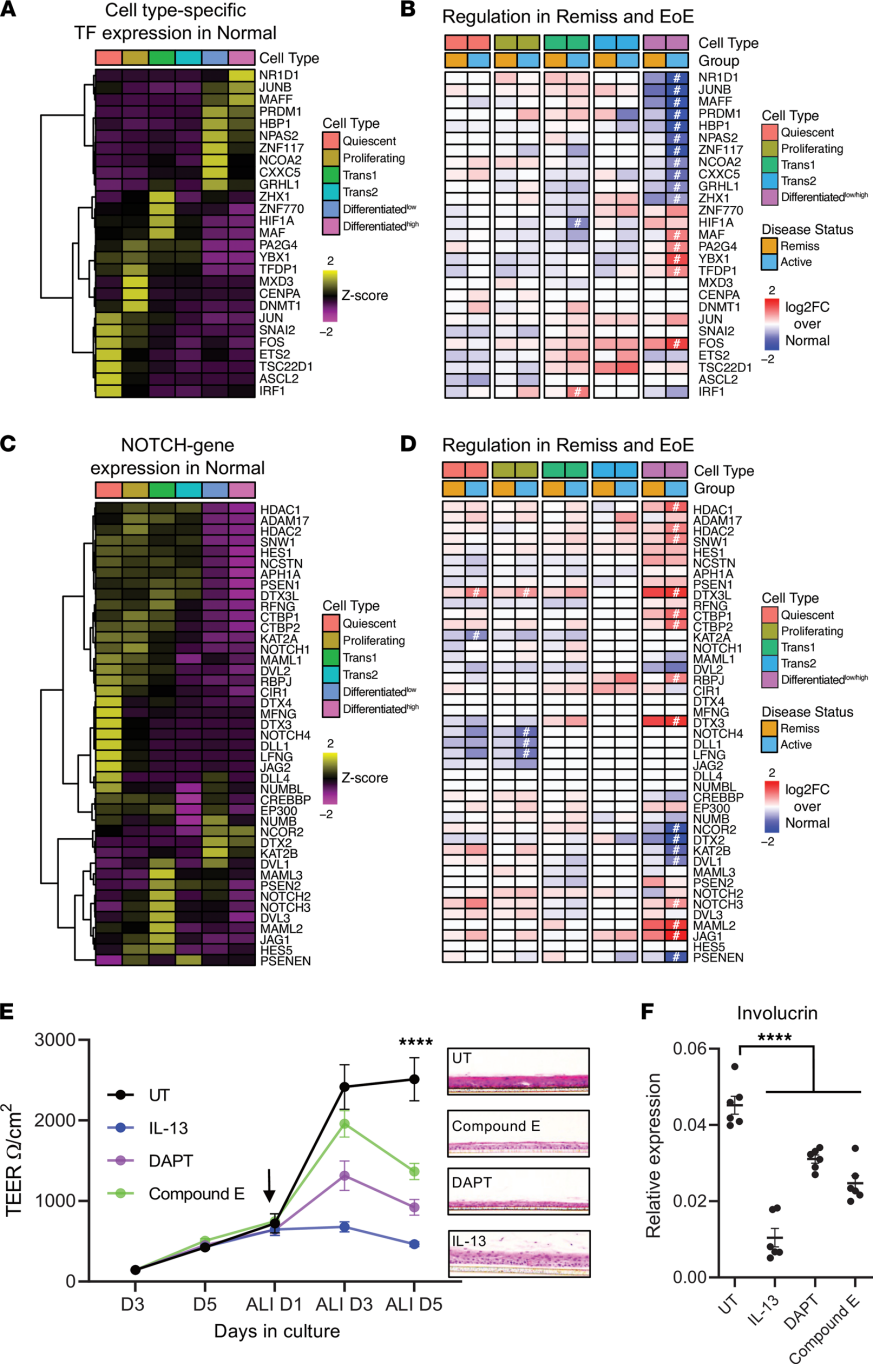
Expression of transcription factors and NOTCH-related genes in human esophageal epithelial cells. (**A**) Gene expression heatmap of subpopulation-specific transcription factors (TF) in the healthy control esophagus (Normal). (**B**) Log_2_FC of the subpopulation-specific TFs in the active EoE (Active) and remission EoE (Remiss) esophagus compared with the normal esophagus by pseudo-bulk RNA-Seq differential analysis (^#^FDR-adjusted *P* < 0.05). Genes are ordered the same as in **B**. (**C**) Gene expression heatmap of the NOTCH signaling pathway genes (KEGG) in the normal epithelial subpopulations. (**D**) Log_2_FC of the NOTCH gene expression in the active EoE (Active) and remission EoE (Remiss) esophagus compared with the normal esophagus by pseudo-bulk RNA-Seq differential analysis (^#^FDR-adjusted *P* < 0.05). Genes are ordered the same as in **C**. For **B** and **D**, Differentiated^hi^ and Differentiated^lo^ cell subpopulations are represented by 1 unified cell population. (**E**) Transepithelial electrical resistance (TEER) was measured in the EPC2 cells grown at the air-liquid interface (ALI) and treated as indicated. Data are from 3–5 independent experiments performed in triplicate. Data are shown as mean ± SEM. ****P < 0.0001 for ALI day 5 (D5) compared with untreated (UT), by 1-way ANOVA. Insets show representative H&E staining of the ALI cultures (×10 magnification). (**F**) Relative expression of involucrin in the EPC2 cells grown at the ALI for 5 days (ALI D5). Expression was normalized to *GAPDH*. Combined data for 3 independent cultures performed in duplicates is shown; data are shown as mean ± SEM. ****P < 0.0001 compared with UT, by 1-way ANOVA.

To explore the molecular basis of the altered differentiation program, we analyzed the expression of transcription factors (TFs) in EEU. First, we identified subpopulation-specific TFs in normal EEU (Figure 6A and Supplemental Figure 6A) and then compared their changes in EoE (Figure 6B and Supplemental Figure 6B). We found that TFs of the Differentiated^hi^ population were significantly downregulated and that TFs of less-differentiated populations were upregulated in active EoE compared with controls (compare average expression heatmap [Figure 6A] to transcriptional change [Figure 6B]). This dichotomic relationship persisted for 134 TFs that were expressed in a differentiation-dependent manner (Supplemental Figure 6A). Expression of most TFs was not statistically different in remission compared with control, with the exception of *ZNF430*, *BHLHE40*, and *XBP1;* the latter remained upregulated in the Differentiated population in active EoE and remission (Supplemental Figure 6A, asterisks). Focusing on the TFs downregulated in the Differentiated^hi^ cells, we generated a high confidence interactome (confidence, 0.9) using the Search Tool for the Retrieval of Interacting Genes/Proteins (STRING) database (18). We found that this interactome included several genes related to nuclear hormone receptor signaling, such as the members of the NR1 subfamily of nuclear hormone receptors *RORA* and *NR1D1*, the retinoic acid receptor *RXRA,* and the transcriptional coactivators for nuclear hormone receptors *NCOA2* and *A3* (Supplemental Figure 6B). Indeed, nuclear receptor signaling genes were significantly enriched in the TFs dysregulated in the most differentiated subpopulation in EoE (χ^2^ test, *P* < 0.0001). In contrast, the interactome of genes that were upregulated in the differentiated cells were enriched for *HIF1A,* which has been previously implicated in EoE pathogenesis (19); *STAT1;* and *TP53* (Supplemental Figure 6C).

We also found that the expression of the NOTCH pathway–related genes were dysregulated in active EoE in several epithelial subpopulations. For example, the NOTCH ligand *JAG1*, coactivator *MAML2,* and major transcriptional effector *RBPJ* were significantly increased in the differentiated cells, whereas the ligand *DLL1* and receptor *NOTCH4* were downregulated in the proliferating population (Figure 6, C and D). Indeed, compared with the untreated cells, cells treated with the NOTCH inhibitors DAPT and Compound E had decreased esophageal epithelial differentiation, including transesophageal electrical resistance (TEER) (Figure 6E, graph). Histologically, IL-13 caused thickening of the epithelium, likely due to increased proliferation, whereas NOTCH inhibitors impaired stratification (Figure 6E, insets). Altered differentiation was accompanied by decreased expression of involucrin (Figure 6F). These results are consistent with prior findings that the NOTCH signaling pathway is a critical regulator of the esophageal epithelium (20). Collectively, these findings suggest that dysregulated expression of TFs specific to the differentiated cells and altered cell-to-cell communications, especially involving the NOTCH family, inhibit the differentiation program of transition-stage epithelial cells in the EEU during allergic inflammation.

### Genetic etiology of food allergy/EoE at the single-cell level.

To assess the contribution of esophageal cell types to the genetic etiology of EoE, we examined the expression of genes previously associated with a broad spectrum of food allergic diseases, including EoE, by genome-wide association study (GWAS), Mendelian association, or candidate gene approaches in cell types identified in the biopsies (7, 8, 21). Remarkably, those genes were expressed in each esophageal cell type, indicating that each cellular subpopulation was likely manifesting part of the genetic risk (Supplemental Figure 7A). Epithelial cells were enriched in susceptibility genes that included the proteases and protease inhibitors *SPINK5*, *SERPINB2*, *SERPINB10*, and *CAPN14*; the junctional proteins *DSG1* and *DSP*; *TSLP;* and the eosinophilic chemoattractant *CCL26*. *TSLP* and *CCL26* were also expressed in fibroblasts. Fibroblasts showed specific expression of the phosphatase *PTEN* and shared expression of the transcriptional repressor *C11ORF30* (*EMSY*) with lymphocytes. *IL33* and *TGFBR2* were preferentially expressed in the endothelial cells, and expression of the leucine-rich membrane protein *LRRC32* was shared between endothelial cells and fibroblasts. The proallergic cytokines *IL5* and *IL13* were enriched in lymphocytes and mast cells.

**Figure 7:**
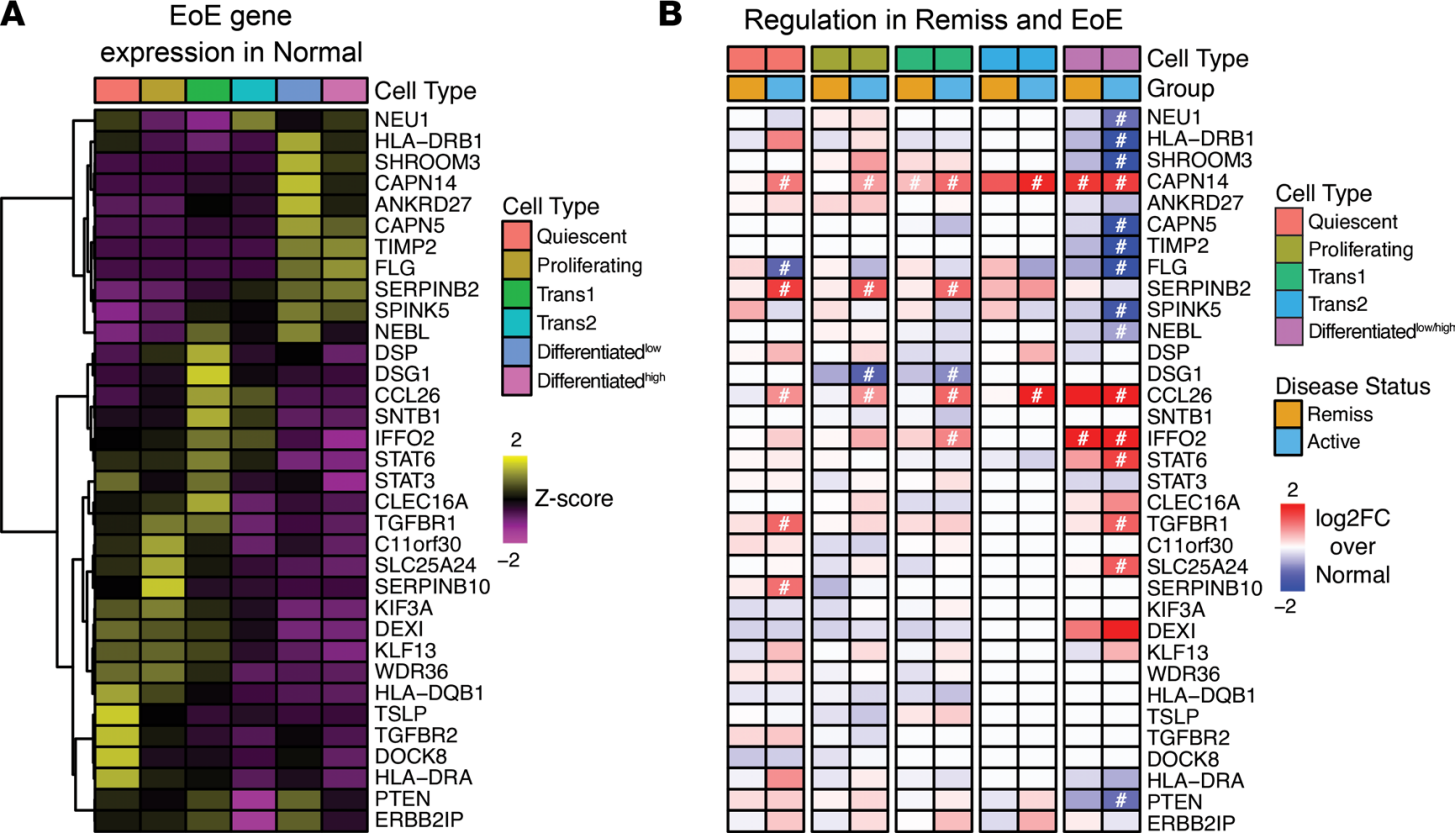
Expression of the epithelial genes genetically linked to EoE and food allergy. (**A**) Gene expression heatmap of 34 genes genetically linked to EoE and food allergy by genome-wide association (GWAS), Mendelian association, or candidate gene approaches delineated by the epithelial subpopulations in the healthy control esophagus (Normal). (**B**) Log_2_FC of the genetically linked genes in the active EoE (Active) and EoE remission (Remiss) esophagus compared with healthy control obtained by pseudo-bulk RNA-Seq differential analysis (^#^FDR-adjusted *P* < 0.05). Genes are ordered the same as in **A**. Note that the Differentiated^hi^ and Differentiated^lo^ cell subpopulations are represented by 1 unified cell population.

Focusing on the EEU, we stratified the expression of the genes genetically linked to EoE within the epithelial subpopulations: 34 genes were robustly detected in at least 1 epithelial subpopulation in the biopsies from healthy controls (Figure 7A), displaying subpopulation-specific expression patterns. Differential analysis by pseudo-bulk RNA-Seq revealed that genes enriched in the Differentiated^lo/hi^ subpopulations (e.g., *FLG*, *SPINK5*) were decreased, whereas genes enriched in less differentiated populations (e.g., *TGFBR1*, *STAT6*) were increased in active EoE (Figure 7B). Notably, expression of *CAPN14*, the protein product of the main EoE susceptibility locus (2p23), and expression of *CCL26*, the critical driver of the eosinophilic infiltration in EoE, paralleled each other, suggesting common regulation in response to IL-13; they were significantly upregulated throughout the EEU. Focusing on the genes significantly dysregulated in the epithelium of patients with active EoE compared with control individuals (Figure 7B), we assessed their expression in the larger cohort of the patients with EoE by bulk RNA-Seq (Supplemental Figure 7B). The majority of the genes showed a similar pattern of transcriptional change, suggesting that the epithelium is a major regulator of their expression. Most active EoE genes had normalized expression in EoE remission with the notable exceptions of *CAPN14* and *IFFO2* (encoding Intermediate Filament Family Orphan 2). These 2 genes remaining significantly upregulated in the Differentiated^lo/hi^ cells suggests a fixed defect. Notably, the EoE risk variant rs28530674 (risk allele frequency 0.04; *P* = 3 × 10^–7^; OR, 1.83) is located within *IFFO2* gene (22). Collectively, these findings demonstrate dynamic disease-dependent expression of genes genetically associated with food allergy/EoE, signify the interplay of the identified heterogeneous esophageal cell types, and delineate the full trajectory of the EEU to allergic inflammatory responses in the esophagus.

## Discussion

Overall, our study provides a global view of cellular and molecular machinery of the esophageal epithelium during homeostasis and an allergically inflamed state at the single-cell level. We report high-resolution transcriptional mapping of the human EEU under homeostatic and inflamed conditions, going beyond classical morphologic analysis of the epithelium. By applying bulk RNA-Seq and scRNA-Seq analyses and pseudotime analysis, we identify the molecular and functional properties and map the spatial localization of 6 major epithelial subpopulations that varied in a disease-dependent manner. We defined the developmental trajectory of the human EEU as the following progression: *KRT15^hi^* quiescent cells → *TOP2A*^+^ proliferating cells → 2 transitioning cell subpopulations defined by expression of *DSP* and *SERPINB3* → 2 *KRT6^hi^* differentiated cell subpopulations that expressed metallothioneins and antimicrobial proteins (e.g., *RNASE7*). By assessing cellular and transcriptional properties of the EEU in the esophageal tissue of individuals with allergic inflammation, we define the dynamic interplay of these epithelial subpopulations as a function of disease state and activity. We determine that allergic inflammation, as assessed in human EoE, is associated with a blockade of the last step of the differentiation program. This differentiation blockade leads to the loss of terminally differentiated cells and hyperproliferation of less mature epithelial subpopulations yet stability of the quiescent, stem cell–enriched basal epithelial compartment. This blockade is associated with and likely driven by a failed transcriptional mechanism involving nuclear receptors in the most differentiated epithelial cells and altered cell-to-cell communication involving the NOTCH signaling pathway. Notably, despite histologic remission of allergic inflammation in inactive EoE, the cellular composition and gene expression signatures of the epithelial subpopulations of EoE remission were distinguishable from those of healthy controls, indicating that remission is a transitory state between the homeostatic and inflamed esophagus and is poised to relapse. We present evidence that susceptibility genes for a range of allergic diseases (e.g., food allergy) are expressed by all 6 epithelial compartments, indicating the importance of each cell subpopulation in different aspects of disease pathogenesis and supporting cooperative pathogenesis at the single-cell level. Collectively, our data highlight the previously unappreciated heterogeneity of the homeostatic and inflamed esophageal epithelium and provide a framework to better understand esophageal health and disease. The collective findings highlight the need for more extensive analysis of various parts of the esophagus in a larger group of patients in order to better understand the contribution of genetics, sex, comorbidities, and intensity of eosinophilic infiltration and to develop clinically relevant molecular diagnostic standards for the disease.

A fundamental property of the EEU that differs between humans and mice is the presence of the quiescent basal layer cells. These human cells have unique morphologic characteristics (e.g., cylindrical cells in contact with lamina propria) and are characterized by the gene expression of structural components (e.g., *KRT15*), TGF-β signaling proteins, HSP70 family chaperones, and epigenome modifiers (*MECP2*, linker histone H1.0 and polycomb repressor complex genes) (3, 23). Under homeostatic conditions, only 2%–5% of the esophageal basal cells are actively dividing (1, 24), consistent with the basal layer being a prime source of the epithelial stem cells. In individuals who undergo chemotherapy and radiation therapy for cancer treatment, approximately 20% of the basal cells of the squamous epithelium of the oral cavity become actively proliferating (25). Remarkably, in the allergic inflamed esophagus, the proportion of basal cell proliferation remains unchanged; most of these cells remain quiescent despite the major increase in the proliferating compartment consistent with the histologically defined basal zone hyperplasia (Figure 4 and ref. 26). These findings suggest the existence of a cell-to-cell communication program that simultaneously increases the proliferation of the upper epithelial layers and preserves the quiescent status of the basal cells. Several signaling pathways have been previously implicated in the regulation of the esophageal epithelial proliferation and differentiation under homeostatic and allergically inflamed conditions, including NOTCH, WNT, and BMP (27–29). Though many of the NOTCH pathway–related genes were dysregulated in EoE, their expression was not limited to the quiescent basal subpopulation. In contrast, insulin-like growth factor binding protein 3 (*IGFBP3*) was increased in EoE and was the most differentially expressed genes in the epithelial basal layer compared with the suprabasal cells. IGFBP proteins are critical regulators of the insulin-like growth factor (IGF) signaling pathway that modulates cell survival, proliferation, and differentiation (30). IGFBPs bind with high affinity to IGF, thereby precluding it from binding to the receptor. Under physiologic and pathologic conditions (e.g., asthma), IGFBPs are subjected to proteolytic degradation by various serine and metalloproteases, thus facilitating IGF binding to the receptor (30). In the homeostatic esophagus, the Trans2 subpopulation is enriched in genes associated with proteolytic activity, whereas in the inflamed esophagus, dysregulated expression of proteases and protease inhibitors occurs throughout the EEU. For example, the cysteine protease *CAPN14* that is normally expressed in the differentiated cells is upregulated in all epithelial subpopulations. The serine peptidase inhibitors *SPINK5* and *SPINK7* are also enriched in the differentiated epithelium in the homeostatic esophagus, but they are depleted in the inflamed esophagus (31). However, *SERPIN*s, whose expression is confined to the Trans2 subpopulation in control biopsies, are highly upregulated in other epithelial subpopulations in active EoE, suggesting that compartmentalization of the proteolytic activity is an integral part of the EoE-altered innate antiinflammatory properties of the esophagus. Whether epithelial proteases of the homeostatic and inflamed esophagus regulate IGFBP processing and subsequent IGF signaling, and the functional consequences of these events, requires further investigation. IGFPB3 also exerts an IGF-independent effect on cell proliferation by regulating NF-κB signaling in a caspase-dependent manner (32); this pathway recently has been implicated in the maturation and release of the critical proallergic alarmin IL-33 that is uniquely expressed in the quiescent basal layer cells in active EoE as part of an allergen-sensing pathway (26, 33). Our results collectively highlight the potentially critical role of the basal layer cells in propagating allergic inflammatory responses in EoE via multiple mechanisms that do not require proliferation.

Functional characteristics of the differentiated epithelial subpopulations are consistent with their primary role in protecting the underlying tissue against environmental insults. Accordingly, the transcriptional signature of the differentiated cells is enriched in genes that regulate keratinization, cornification, cell-to-cell junction organization, and antibacterial responses. The Differentiated^hi^ subpopulation specifically expresses ribonuclease A family member 7 (*RNASE7*), a protein that has broad-spectrum antimicrobial activity against bacteria and fungi. Loss of *RNASE7* expression may be related to the dysbiosis observed in individuals with active EoE (34, 35). Moreover, differentiated cells express peptidyl arginine deiminase (PADI) and transglutaminase (TGM) genes that drive calcium-dependent protein deamination (citrullination) and structural protein crosslinking. Although these processes have been primarily implicated in keratinization of the skin epithelium and hair development (36, 37), esophagus-specific expression of *PADI1*, *PADI3*, and *TGM3* in the differentiated cells in the EEU and their loss in active EoE underscore that overlapping molecular mechanisms are involved in the formation of the esophageal epithelial barrier. This notion is further supported by a damaging mutation in *PADI3* being associated with uncombable hair syndrome, central centrifugal cicatricial alopecia, and active EoE (38, 39).

Another mechanism of the barrier formation by the differentiated esophageal epithelium may involve nuclear receptors, such as retinoid acid receptor RXRA and a member of the NR1 subfamily RORA. These are ligand-regulated TFs that have been implicated in epithelial differentiation and skin homeostasis (40, 41). Notably, TFs that were downregulated in the most differentiated epithelial cells in active EoE compared with control biopsies were highly enriched in the nuclear hormone receptors. Moreover, *RORA* recently has been identified as a genetic susceptibility risk locus in EoE by a GWAS (42), collectively signifying the potential role of this pathway in the protective function of the homeostatic esophageal epithelium.

Differentiated^hi^ epithelial cells are characterized by specific expression of cysteine-rich metal-binding proteins, called metallothioneins, that sequester zinc, copper, and heavy metal ions (43). These proteins emerge as modulators of innate and adaptive immunologic responses, primarily as regulators of zinc metabolism and protection of cells against oxidative stress (44). Increased production of reactive oxygen species has been linked to the imbalance between self-renewal and differentiation of the esophageal epithelial cells caused by altered BMP signaling in EoE (28). Metallothionein expression is dramatically decreased in active EoE, likely due to loss of the Differentiated^lo/hi^ subpopulations, and it is induced by proton pump inhibitors, which are widely used for treating EoE (45). These findings suggest involvement of zinc- and copper-mediated processes in EoE pathogenesis and warrant further investigation of the immunomodulatory properties of metallothioneins in the esophageal epithelium.

Chronicity is a leading feature of EoE, as clinical and histologic features of esophageal inflammation persist over time in most individuals (46). Despite initial improvement in symptoms, endoscopic characteristics, and histology of the esophageal biopsies in response to pharmacologic and dietary interventions, most individuals quickly relapse after cessation of medications, consistent with a fraction of the EoE transcriptome being refractory to treatment (47–49). The disease remission is primarily defined by the histologic assessment of the eosinophil density in the esophageal biopsies, which is not always sufficient to explain the symptoms. For example, pain is not well correlated with eosinophil counts (50). Our analysis reveals that the cellular composition of the esophageal biopsies in disease remission is marked by the decreased proportion of the differentiated compartment and increased proportion of the proliferating and transitioning compartments compared with those of healthy controls. Despite largely normalized gene expression and histologic remission in inactive disease, the single-cell epithelial differentiation program blocked at the differentiation stage in active EoE is only partially reversed in inactive disease, indicating that remission is a transitory state between the homeostatic and inflamed esophagus and is poised to relapse. It is tempting to speculate that esophageal epithelial cells maintain epithelial inflammatory memory similar to their skin counterparts (51, 52) and, thus, contribute to disease relapses. In the homeostatic esophageal epithelium, expression of *CAPN14*, which is genetically linked in EoE, is confined to the Differentiated^lo/hi^ subpopulations. In contrast, in active EoE *CAPN14* expression is detected in the transitioning and quiescent cells, where it remains highly expressed even in EoE remission. Notably, CAPN14-mediated cleavage of DSG1, PPL, and DSP can trigger the loss of epithelial barrier integrity (53, 54). As TSLP release is highly responsive to the loss of epithelial barrier integrity (55) and the quiescent subpopulation specifically expresses *TSLP* (Figure 2B), these findings provide a plausible mechanism for disease relapse and underscore the necessity for improved diagnostic criteria for disease remission based on a molecular, single-cell profiling platform.

## Methods

### Subject inclusion criteria and biopsy sample acquisition.

Biopsies and blood samples were acquired systemically from individuals who were having an endoscopy for EoE or related symptoms at CCHMC. EoE was defined by a histologic finding of ≥ 15 esophageal eosinophils per microscopic high-power field with clinical symptoms. EoE remission (inactive EoE control) was defined by a history of EoE and an esophageal tissue eosinophil count of < 2 eosinophils/HPF at the time of biopsy. Healthy controls were defined by no history of EoE and a negative endoscopy with 0 eosinophils/HPF at the time of biopsy. Individuals’ clinical and demographic information was obtained from the electronic medical record and research questionnaires.

### Endoscopic biopsy processing.

For each sample, 3 biopsies from the distal esophagus of a donor were collected into RPMI medium with 10% FBS, kept on ice, and transported to the research laboratory within 30 minutes. The biopsies were then transferred into EDTA buffer (5 mM EDTA, 10% FBS, 1 mM HEPES in PBS) for 15 minutes in a 37°C water bath, washed once with PBS, minced, and then subjected to collagenase A digestion (Roche, 10103578001, derived from Clostridium histolyticum, 2 mg/mL) in 10% FBS-RPMI at 37°C for 30 minutes. The resulting suspension was diluted with 10 mL ice-cold PBS, passed through a 19 gauge needle 5 times, filtered through 2 layers of gauze, washed twice with ice-cold PBS (10 mL each), and then centrifuged at 450*g* for 5 minutes at 4°C, resulting in a visible, clear-edged, translucent pellet. Isolated cells were subjected to 10X Genomics sequencing in the CCHMC Gene Expression Core according to the manufacturer instructions.

### FACS.

Single-cell suspensions were generated from biopsies, and FACS was performed on ice for 30 minutes with the following antibodies: Invitrogen Live/Dead Yellow (catalog L34959, Thermo Fisher Scientific, LIVE/DEAD™ Fixable Yellow Dead Cell Stain Kit for 405-nm excitation), human PDPN PE (catalog 337003), human CD45 APC-Cy7 (catalog 304014), and human E-cadherin (E-cad) APC (catalog 324108) (all purchased from BioLegend Inc.). The 2 populations of interest were sorted by MoFlo machine under 2-way sorting settings with representative sorting chart shown (Figure 3A). Live singlet events were preselected by a Live/Dead+ FSC-A/FSC-H diagonal gate. The PDPN^hi^ population was defined and sorted as live CD45-PDPN^hi^E-cad^hi^ events, whereas the PDPN^lo^ population was defined as live CD45-PDPN^lo^E-cad^hi^ events. Sorting typically lasted around 30–45 minutes, with frequent agitation to collect the droplets attaching to the collection tube wall. Three biopsies collected from the distal esophagus typically yielded approximately 2500–10,000 epithelial cells in each subpopulation; they were subsequently collected into RPMI-1640 media supplemented with 10% FBS and pelleted/centrifuged for immediate RNA isolation procedures using RNeasy Micro Kit (Qiagen).

### Bulk RNA-Seq of FACS-sorted cells.

Total RNA was amplified using the Ovation RNA-Seq System v2 assay (Tecan Genomics) according to the manufacturer’s protocol. The libraries were prepared with the Nextera XT DNA Sample Preparation kit (Illumina Technologies). In total, 1 ng of cDNA was resuspended in Tagment DNA Buffer, and tagmentation (fragmentation and tagging with the adaptors) was performed with the Nextera enzyme (Amplicon Tagment Mix), incubating at 55°C for 10 minutes. Neutralize Tagment (NT) buffer was then added to neutralize the samples (part of the Nextera XT DNA Sample Preparation kit, Illumina Technologies). Libraries were prepared by PCR with the random primer Nextera PCR Master Mix and 2 Nextera Indexes (N7XX and N5XX) according to the following program: 1 cycle of 72°C for 3 minutes; 1 cycle of 98°C for 30 seconds; 12 cycles of 95°C for 10 seconds, 55°C for 30 seconds, and 72°C for 1 minute; and 1 cycle of 72°C for 5 minutes. The size of the libraries for each sample was measured using the Agilent HS DNA chip (Agilent Technologies). The samples were placed in a pool. The concentration of the pool was optimized to acquire at least 25 million to 30 million reads per sample. Libraries were sequenced on the Illumina HiSeq2500 following the manufacturer protocol using Paired-End Reads with a read length of 75 bps. RNA-Seq analysis was performed by BioWardrobe, as previously described (56).

### Colony-forming ex vivo assay and immunofluorescence of the colonies.

PDPN^hi^ and PDPN^lo^ cells were sorted in 200 mL of the SCM-68 medium, counted, and seeded in a 96-well plate together with 30,000 irradiated mouse fibroblasts NIH 3T3 that served as a feeder layer. SCM-68 medium contained freshly prepared cFAD medium and supplements. cFAD medium components were DMEM + Ham’s F12 ratio 3:1 v/v; FBS (2% final; Atlanta Biologicals, S10295); human insulin (5 mg/mL) (MilliporeSigma, 91077C); adenine (24.3 mg/mL) (MilliporeSigma, A2786); 3,3,5-triiodo-L-thyronine (T3) (2 × 10^–9^ M) (MilliporeSigma, T6397); hydrocortisone (0.4 mg/mL) (MilliporeSigma, H0888); cholera toxin (1 × 10^–10^ M), (MilliporeSigma, 227036); and antibiotic-antimycotic solution (Thermo Fisher Scientific, 15-240-096). Supplements included recombinant human R-Spondin 1 protein, CF (125 ng/mL) (R&D, 4645-RS/CF); Jagged-1 (1 mM) (AnaSpec, AS-61298); human Noggin (100 ng/mL) (Peprotech, 120-10C); Rock-inhibitor (2.5 mM) (InSolution, Y-27632); SB431542 (2 mM) (Cayman Chemicals, SB-431542); and Nicotinamide (10 mM) (MilliporeSigma, N0636). Colonies were typically counted 3–5 days after seeding. For immunofluorescence, primary epithelial cells were isolated by FACS as above and seeded at 20,000–50,000 cells with 30,000 feeder layer fibroblasts per well on the 8-well ibiTreat chambers (Ibidi, 80826) for 3 days before staining and imaging following a standard protocol. Antibodies used for staining were ITGA6 (BD Pharmingen, 551129), E-cad (R&D, AF648), SOX2 (Cell Signaling Technology, 3579), and cytokeratin 5 (Abcam, ab24647), all at a 1/200 dilution in 2% donkey serum (The Jackson Laboratory). Affinity-purified polyclonal secondary antibodies from the donkey serum were purchased from The Jackson Laboratory (stock no. 017-000-121). DNA was stained with Hoechst 33342 Solution (Thermo Fisher Scientific). Imaging was performed with a Nikon A1 inverted confocal microscope.

### Air-liquid interphase cultures.

Cells from the esophageal epithelial TERT-immortalized cell line EPC2 (a gift from Anil Rustgi, University of Pennsylvania, Philadelphia, Pennsylvania, USA) were differentiated following an air-liquid interface (ALI) protocol on 0.4 μm pore polyester permeable supports (Corning) in keratinocyte serum–free media (KSFM) (Invitrogen) as previously described (45). Cultures were treated with the NOTCH inhibitors DAPT (final concentration 20 μM) and Compound E (final concentration 1 μM) and with IL-13 (final concentration 100 ng/mL) starting at day 1 of the exposure to air. Transepithelial electrical resistance (TEER) was measured using an EVOM (World Precision Instruments).

### scRNA-Seq library preparation.

Single-cell suspensions were prepared from biopsies as described above. Bulk population cells were directly subjected to the 10X mass genomics chip (10X Genomics Inc.), targeting 10,000 simultaneously captured live events. Each cell was uniquely barcoded during the cDNA library generation by Single Cell 3′ Reagent Kits v2 per the manufacturer instructions and subsequently sequenced on an Illumina HiSeq 2500 at the CCHMC DNA Sequencing and Genotyping Core.

### scRNA-Seq data analysis.

All scRNA-Seq data were processed using Cell Ranger version 3.0.2 and the hg19 reference. The UMI count matrix was imported to Seurat (10) for postprocessing and downstream analysis. For each sample, doublets were first filtered out using Scrublet (57). Then, cells either with high mitochondrial reads, a low number of detected genes, or excess UMI counts were discarded. The UMI count matrix was normalized and scaled following the standard Seurat pipeline by adjusting for mitochondrial read counts and UMI counts. After the post processing, samples were integrated in 2 different ways: (a) by the disease status (i.e., healthy control, EoE remission, and active EoE) and (b) by all 10 samples using Seurat to compare the cellular population repertoire in an unbiased manner. Preliminary clustering was performed using the top 20 principal components to detect major cell types first, including epithelial cells and others, and this information was projected onto UMAP (58). Also, marker genes were called for cell type confirmation, detecting genes exclusively expressed in distinct populations. To do so, we performed a series of pairwise differential expression analyses against each of all the other cell clusters using the “FindMarkers” function in Seurat with the parameters log_2_FC > 0.25 and minimal proportion of cells expressing a gene (min.pct) > 0.1, and we selected genes that were commonly highly expressed in each cluster. Epithelial cells were selected for the second-round clustering to identify subpopulations, and marker gene identification was performed the same way. This process was repeated for all 10-sample integrations and for each group-wise integration independently to confirm the similar cellular repertoire of epithelial subpopulations. The proportion of each cell type was measured for each sample and was compared between the different disease statuses using a 2-tailed Student’s *t* test to examine the change by the disease status. Detailed maps of quiescent and proliferating cells were also examined by combining PDPN^lo^ or PDPN^hi^ marker gene expression patterns and cell cycle mapping by Seurat in a UMAP. Pseudotime analysis was performed using Slingshot for all 10-sample integrations to map each cell from different groups onto a common pseudotime axis, which corresponded to the human esophageal epithelial differential trajectory. Cell densities along the epithelial differentiation pseudotime were visualized and compared across the different EoE statuses using a density plot, and a 2-tailed Student’s *t* test was performed to test the significance. Likewise, the same *t* test was performed to compare cell density changes in quiescent basal, proliferating basal, and proliferating suprabasal cells. Overall sample-to-sample similarity in the epithelial cellular repertoire was visualized by hierarchical clustering based on the epithelial subpopulation proportion vector using a Pearson correlation coefficient as a similarity measure under Ward’s criteria (59).

When comparing the expression profile of epithelial subpopulations between different EoE statuses, we sought to detect robust changes by utilizing the statistical power of the biological replicates. Therefore, rather than simply pooling all cells from biological replicates into single subpopulation clusters, we converted the UMI matrix into pseudo-bulk RNA-Seq data by summing UMI counts per gene for each sample per epithelial subpopulation, using a total of 50 pseudo-bulk RNA-Seq data (10 samples × 5 clusters). We subsequently performed differential gene expression analysis using DESseq2 (17) comparing healthy control versus EoE remission and healthy control versus active EoE. Thus, we were able to detect robust sets of EoE-dysregulated genes not biased to a specific sample. Genes with a FC > 1.5 and FDR-adjusted *P* < 0.05 were selected as differential genes. GO analysis was performed using Enrichr (60). We noticed that many of the EoE differentiation genes were dysregulated, especially in the differentiated cells. Thus, we performed another differential analysis comparing the extreme 2 ends of cell clusters, quiescent cells and differentiated cells, for each of the disease status (healthy control, EoE remission, active EoE) samples. Genes differentially expressed as a function of epithelial differentiation and by EoE status for each epithelial subpopulation were pooled and subjected to hierarchical clustering to examine the EoE-dependent dysregulation of gene expression required for epithelial differentiation. We identified 3 types of patterns: (a) Type 1 were severely dysregulated (induce or repressed) in active EoE versus healthy control; (b) Type 2 were moderately dysregulated (induced or repressed) in active EoE versus healthy control; and (c) Type 3 were normally expressed (i.e., a similar level of expression as that of healthy control). GO analysis was performed for these groups of genes using Enrichr (60). The list of all human TFs was obtained from Lambert et al. (61) for the visualization of their expression level in epithelial subpopulations and their changes by disease status. TF expression was visualized in 2 ways: a short list and an extended list. The short list of TFs was prepared by identifying exclusively expressed TFs in each subpopulation, and the extended list was prepared by identifying TFs that display altered expression between any subpopulations.

### Data availability.

Next-generation sequencing data have been uploaded to GEO under the accession nos. GSE201154 (bulk RNA-Seq) and GSE201153 (scRNA-Seq).

### Statistics.

Statistical results were performed with GraphPad Prism, version 9, software. Data are reported as mean ± SD. For comparison of 2 groups, statistical significance was determined by unpaired, 2-tailed Student’s *t* tests. For comparison of more than 2 groups, 1-way ANOVA was used. *P* < 0.05 was considered significant. The χ^2^ test with Yates correction with the 2-tailed *P* value was used.

### Study approval.

Samples were obtained following informed consent under the auspices of the IRB of CCHMC (no. 2008-0090).

## Author contributions

TW and MER designed the study; TW and MR performed experiments; MK, PJB, NBBM, and HWL analyzed the data; MR, JMC, HWL, and MER wrote the manuscript; and MER supervised the study. All authors reviewed and edited the manuscript.

## Supplementary Material

Supplemental data

Supplemental table 1

Supplemental table 2

Supplemental table 3

Supplemental table 4

## Version 1 04/26/2022

In-Press Preview

## Version 2 06/08/2022

Electronic publication

## Version 3 06/17/2022

GEO Accession Number Corrected
