# Supplementary material for: Single-cell RNA-Seq of human esophageal epithelium in homeostasis and allergic inflammation
Source: JCI Insight. 2022 Jun 8;7(11):e159093. doi: 10.1172/jci.insight.159093 (PMC9208762; doi:10.1172/jci.insight.159093)
Supplement: Supplemental data [file jciinsight-7-159093-s298.pdf]

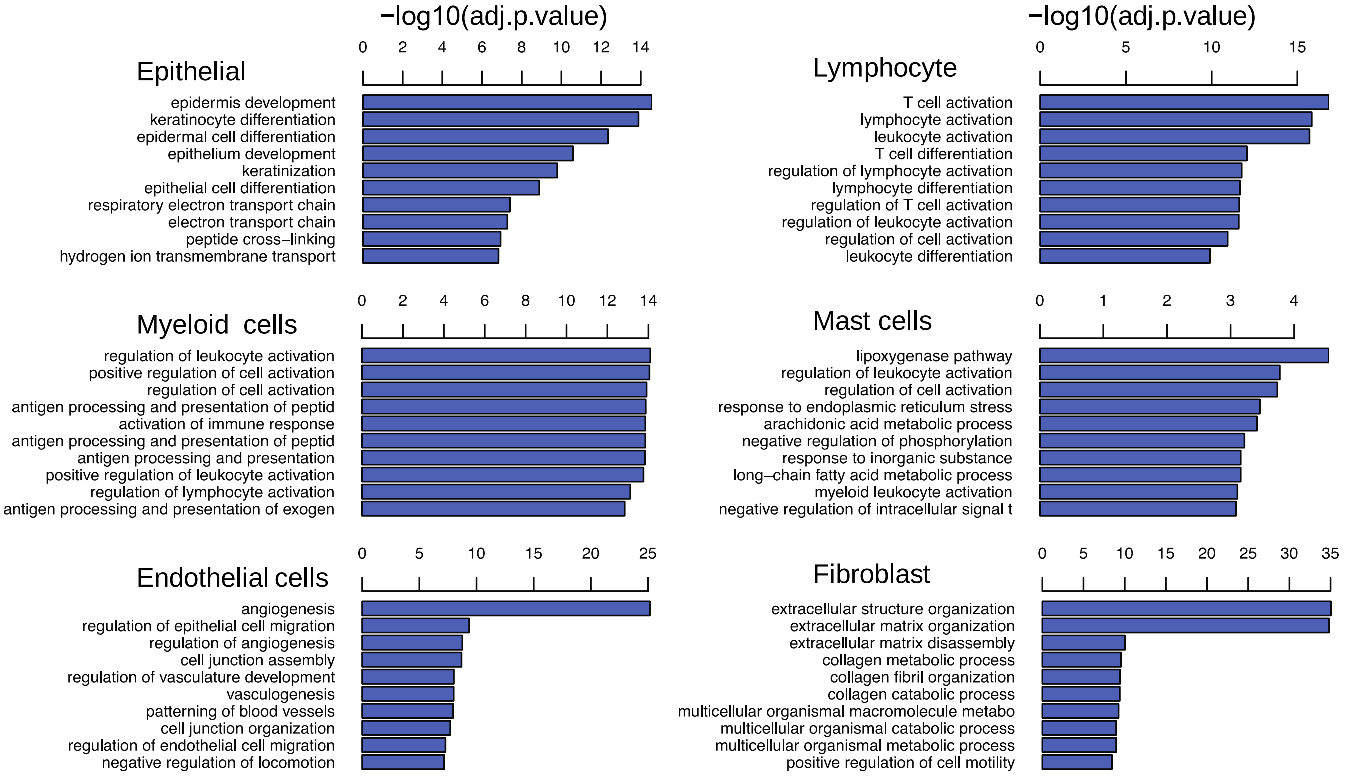

Supplemental Figure 1

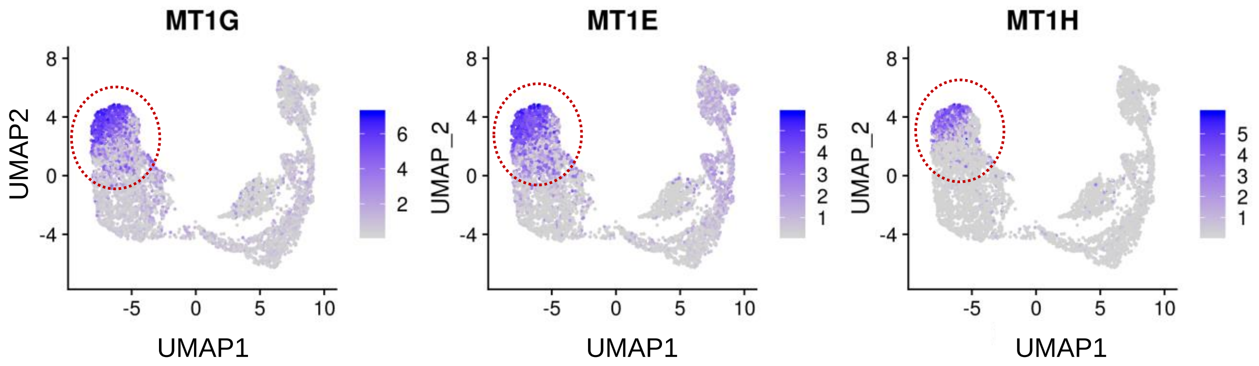

Supplemental Figure 2

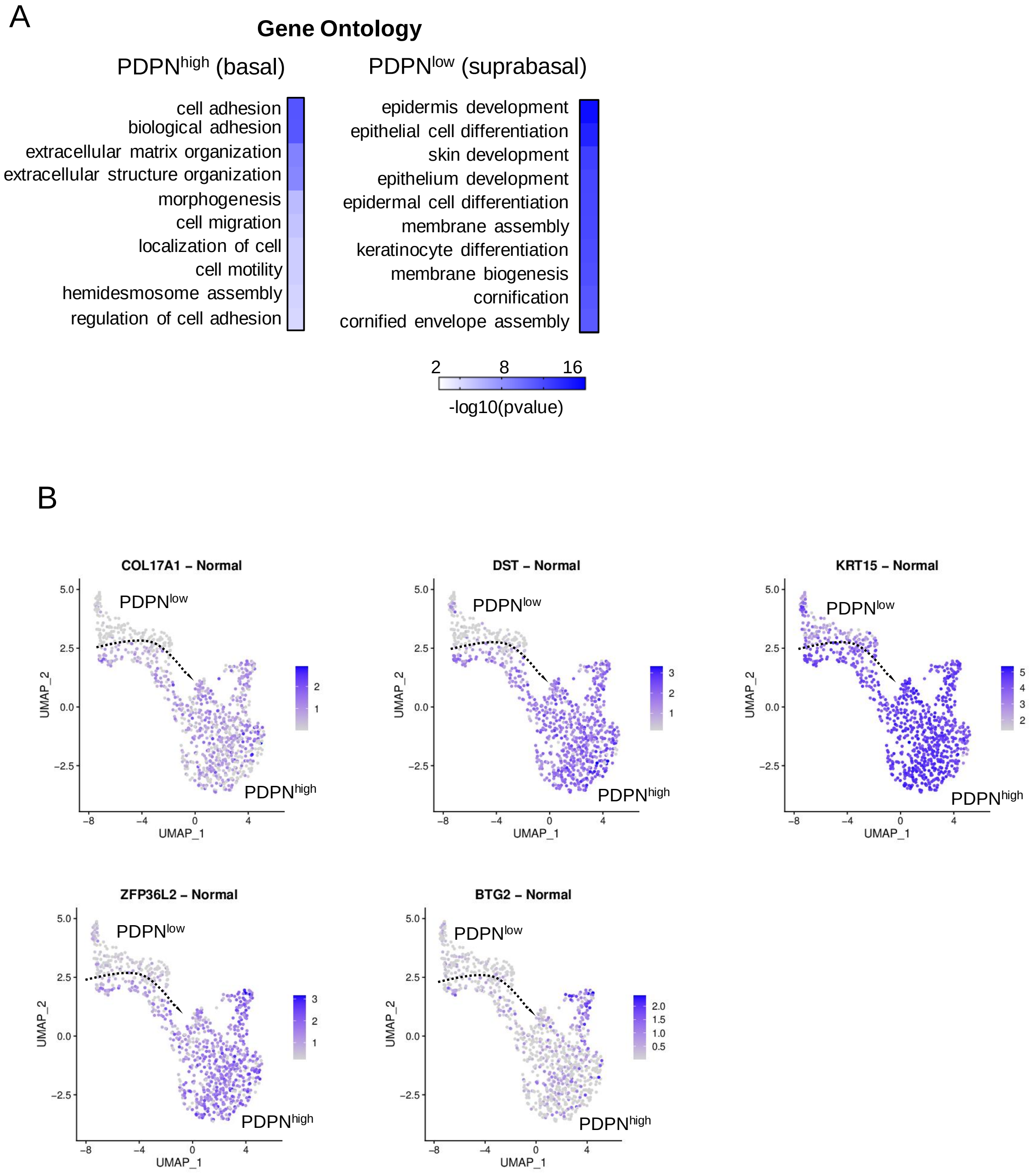

Supplemental Figure 3

A

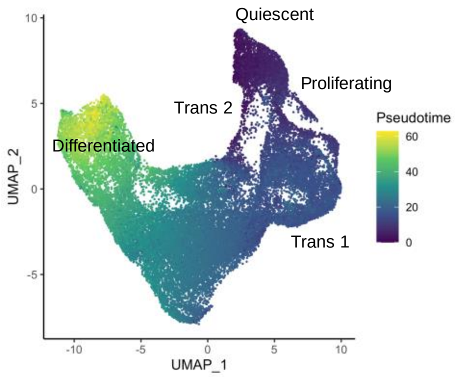

B

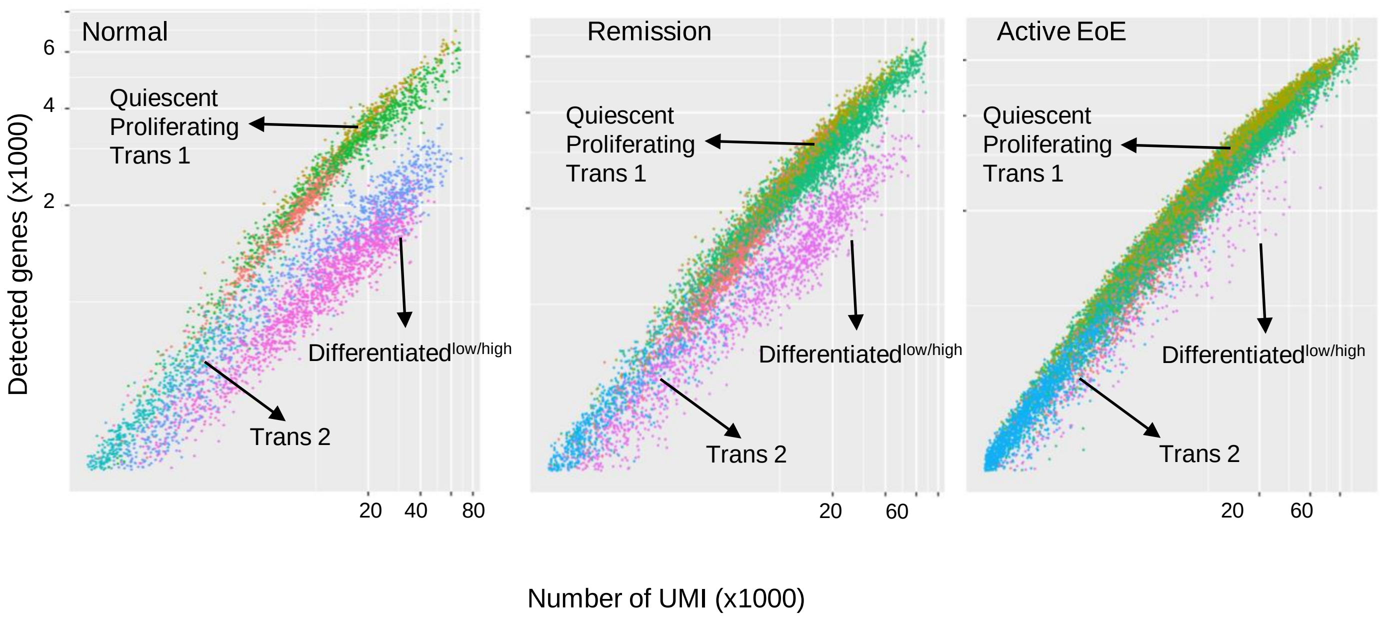

Supplemental Figure 4

A

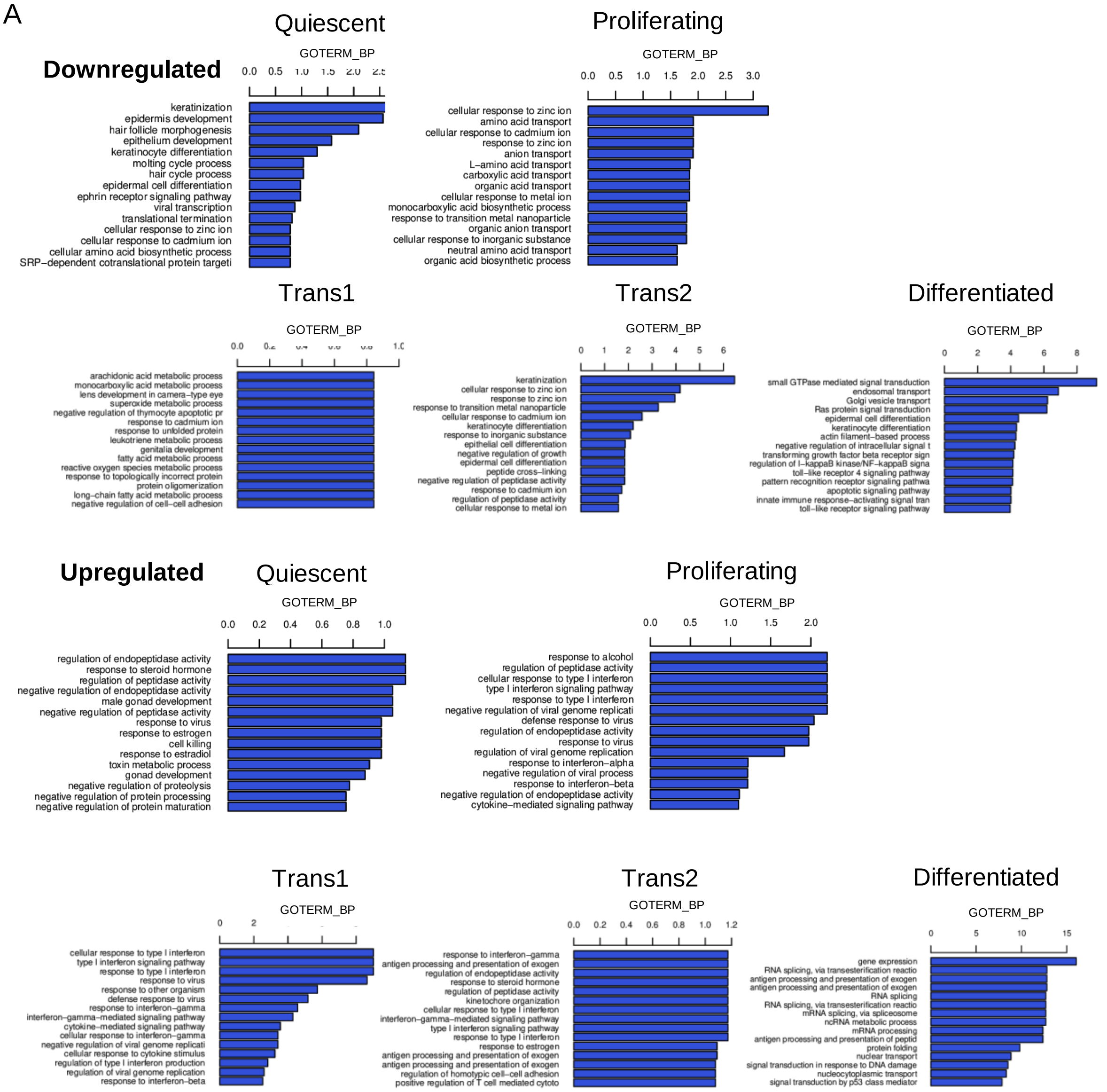

B

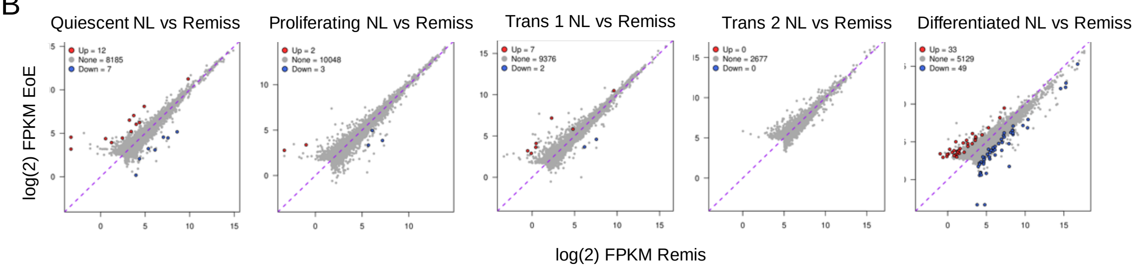

Supplemental Figure 5

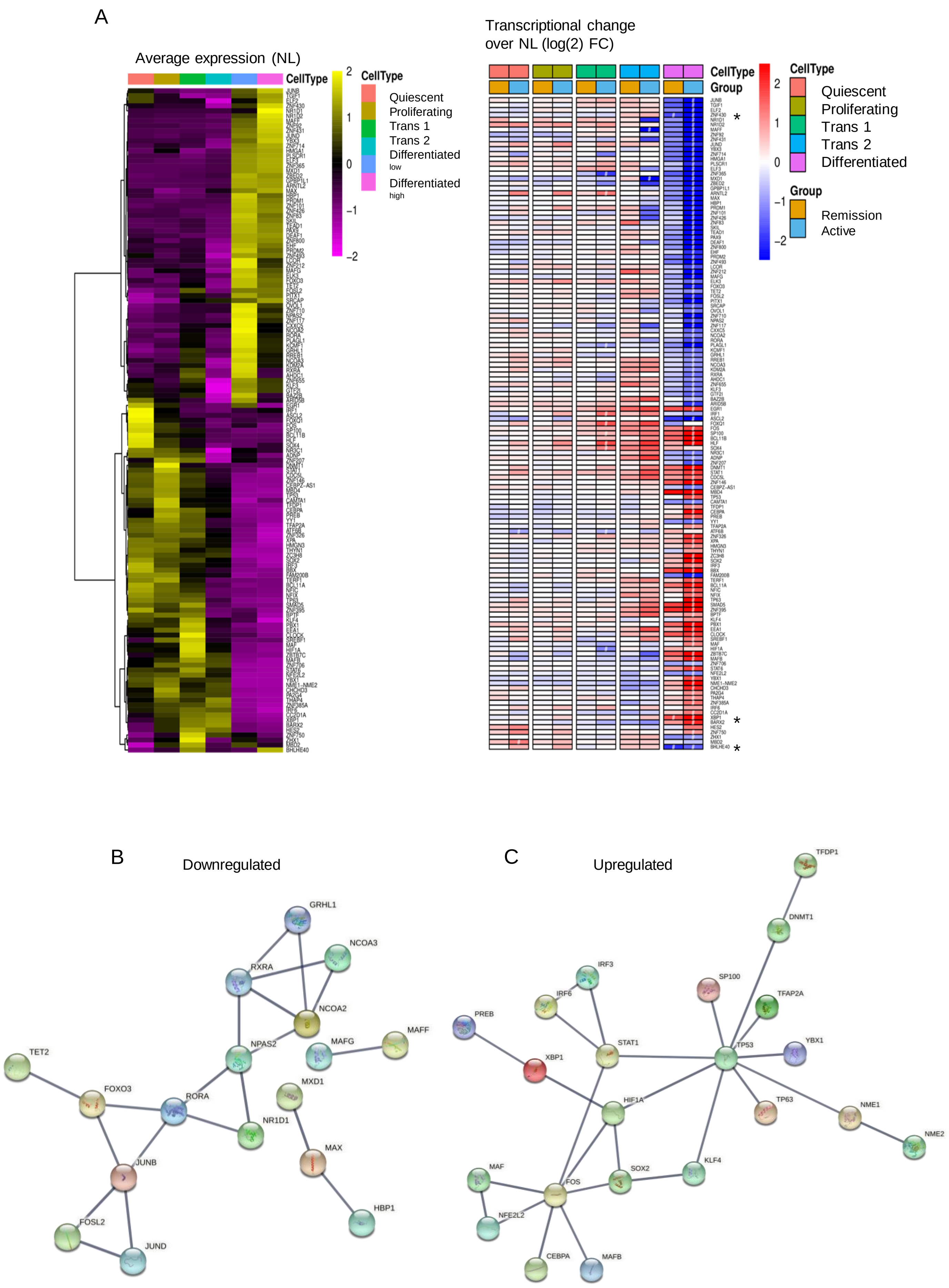

Supplemental Figure 6

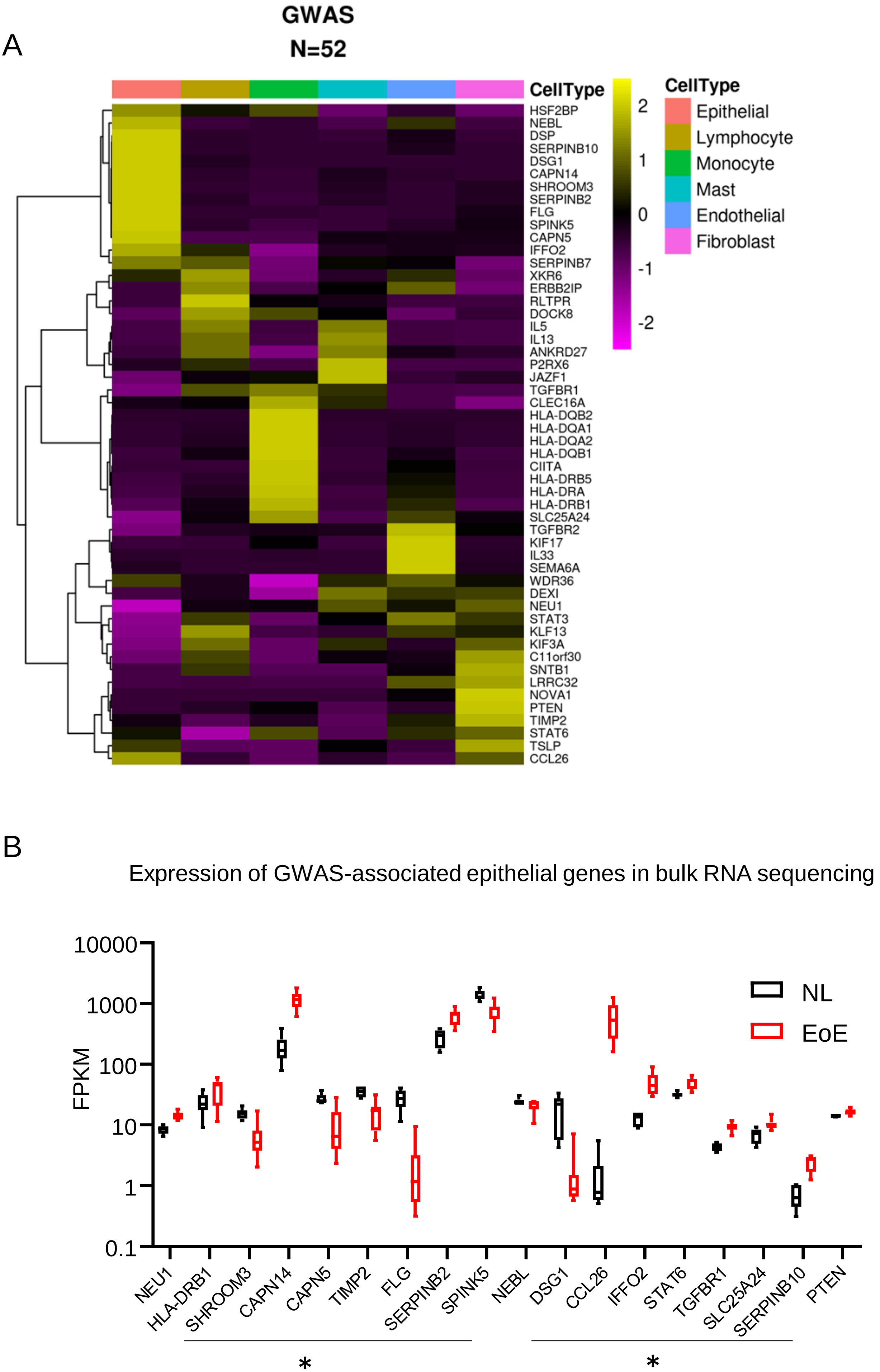

Supplemental Figure 7
