# Supplementary material for: Single-cell RNA-Seq of human esophageal epithelium in homeostasis and allergic inflammation
Source: JCI Insight. 2022 Jun 8;7(11):e159093. doi: 10.1172/jci.insight.159093 (PMC9208762; doi:10.1172/jci.insight.159093)
Supplement: Supplemental table 1 [file jciinsight-7-159093-s299.pdf]

Supplemental Table 1. Markers of cell types defined in human esophageal biopsies

gene	logfc_min	adjp_max	cell type
FAM25A	2.52	2.63711E-25	Epithelial
CRCT1	2.48	3.48371E-10	Epithelial
SPRR3	2.44	5.86954E-25	Epithelial
SPRR2D	2.41	6.91323E-24	Epithelial
SPRR2A	2.40	1.17387E-43	Epithelial
CSTA	2.37	1.91E-286	Epithelial
S100A2	2.35	0	Epithelial
S100A8	2.35	3.2851E-110	Epithelial
CSTB	2.28	2.0581E-170	Epithelial
KRT15	2.27	1.6021E-158	Epithelial
S100A14	2.27	0	Epithelial
S100A9	2.25	4.77E-134	Epithelial
MAL	2.23	1.80918E-53	Epithelial
KRT13	2.22	1.1053E-240	Epithelial
KRT4	2.22	5.5932E-111	Epithelial
CRNN	2.21	7.57608E-50	Epithelial
SPINK5	2.14	2.6257E-171	Epithelial
KRT19	2.12	3.2511E-237	Epithelial
SPRR1B	2.09	2.3192E-93	Epithelial
LY6D	2.04	3.3703E-206	Epithelial
RHCG	2.04	4.5585E-191	Epithelial
KRT5	1.99	4.0849E-187	Epithelial
SPRR1A	1.99	1.71331E-75	Epithelial
KRT78	1.97	2.75045E-29	Epithelial
SERPINB3	1.95	1.4186E-177	Epithelial
AQP3	1.93	1.1087E-245	Epithelial
MIR205HG	1.89	1.2417E-189	Epithelial
TMPRSS11B	1.87	1.82401E-24	Epithelial
SFN	1.85	3.3424E-245	Epithelial
KRT6A	1.82	1.532E-210	Epithelial
PERP	1.80	1.0708E-290	Epithelial
FXYD3	1.71	1.0602E-245	Epithelial
LYPD2	1.70	6.31601E-21	Epithelial
FABP5	1.70	3.2951E-121	Epithelial
EMP1	1.68	5.81865E-35	Epithelial
S100P	1.68	4.51351E-34	Epithelial
TGM3	1.66	2.25588E-52	Epithelial
SPRR2E	1.65	7.25342E-32	Epithelial
DSP	1.59	1.2432E-251	Epithelial

CRABP2	1.58	1.2519E-139	Epithelial
TXN	1.57	3.2772E-200	Epithelial
FLG	1.57	1.61785E-08	Epithelial
MT1G	1.54	7.39294E-17	Epithelial
SERPINB4	1.54	1.0903E-99	Epithelial
PI3	1.54	3.51393E-54	Epithelial
CALML3	1.52	2.4917E-174	Epithelial
KRT14	1.51	1.56334E-30	Epithelial
IL1RN	1.50	3.8333E-125	Epithelial
PPL	1.48	1.0465E-131	Epithelial
C19orf33	1.44	1.4077E-246	Epithelial
SPINK7	1.44	1.9309E-16	Epithelial
LYPD3	1.43	1.0904E-177	Epithelial
ERO1L	1.39	1.58565E-40	Epithelial
LCN2	1.39	4.88444E-63	Epithelial
SCEL	1.38	4.7283E-126	Epithelial
SLPI	1.37	2.4053E-123	Epithelial
MT1X	1.37	1.40819E-88	Epithelial
HSPB1	1.36	1.512E-193	Epithelial
TACSTD2	1.33	4.1431E-233	Epithelial
KRT16	1.32	2.11151E-78	Epithelial
PSCA	1.32	2.02122E-18	Epithelial
MUC21	1.31	1.04406E-28	Epithelial
ALDH3A1	1.27	9.5824E-172	Epithelial
PRSS27	1.26	7.45898E-35	Epithelial
GSTP1	1.20	1.5141E-184	Epithelial
C9orf169	1.20	1.51186E-67	Epithelial
GBP6	1.20	6.9692E-144	Epithelial
KRT6B	1.20	1.39208E-98	Epithelial
LYNX1	1.20	4.81483E-56	Epithelial
DAPL1	1.19	4.6895E-142	Epithelial
ECM1	1.18	1	Epithelial
SBSN	1.18	7.36337E-63	Epithelial
KLF5	1.14	1.6785E-197	Epithelial
DSG3	1.13	6.5922E-151	Epithelial
NCCRP1	1.11	8.7252E-52	Epithelial
A2ML1	1.11	2.84241E-92	Epithelial
MAL2	1.09	1.693E-163	Epithelial
CLDN7	1.08	5.7239E-184	Epithelial
LGALS7B	1.05	9.6335E-113	Epithelial
KLK11	1.04	3.547E-166	Epithelial
RAB25	1.04	1.4692E-170	Epithelial
CRISP3	1.03	1.64062E-17	Epithelial

TMEM40	1.01	3.8975E-136	Epithelial
S100A16	1.01	2.1839E-273	Epithelial
CAPN14	1.01	1.31681E-85	Epithelial
GJB2	1.00	8.1541E-108	Epithelial
PDZK1IP1	1.00	6.4428E-136	Epithelial
DSC3	0.99	2.5571E-150	Epithelial
FAM129B	0.98	4.5393E-102	Epithelial
EPS8L1	0.98	2.83361E-22	Epithelial
SDC1	0.98	1.5049E-164	Epithelial
PITX1	0.97	7.2247E-139	Epithelial
ELF3	0.96	7.4642E-102	Epithelial
SERPINB13	0.96	7.2959E-129	Epithelial
PKP3	0.96	8.7595E-149	Epithelial
DSC2	0.96	7.5028E-122	Epithelial
TMPRSS11E	0.96	3.17959E-41	Epithelial
TXNDC17	0.96	1.4899E-188	Epithelial
S100A11	0.94	1.3353E-204	Epithelial
CLCA4	0.94	9.49745E-73	Epithelial
TRIM29	0.94	6.9995E-160	Epithelial
MGST1	0.93	7.5246E-125	Epithelial
MUC1	0.93	2.73329E-30	Epithelial
SERPINB2	0.92	2.25276E-96	Epithelial
JUP	0.92	2.1293E-170	Epithelial
FGFBP1	0.92	3.171E-87	Epithelial
SERPINB5	0.91	7.2069E-133	Epithelial
ANXA1	0.91	6.505E-166	Epithelial
ADH7	0.89	2.6564E-137	Epithelial
SLURP1	0.89	4.19654E-34	Epithelial
LMO7	0.88	1.03798E-66	Epithelial
RHOV	0.87	9.2743E-121	Epithelial
PKP1	0.86	8.1012E-132	Epithelial
CLDN4	0.86	2.2897E-99	Epithelial
HILPDA	0.85	3.33448E-20	Epithelial
MXD1	0.83	1.16923E-07	Epithelial
CEACAM6	0.83	5.51263E-56	Epithelial
LGALS7	0.80	1.60954E-73	Epithelial
PGD	0.79	8.3469E-147	Epithelial
C6orf132	0.78	2.8189E-93	Epithelial
CLCA2	0.78	1.9951E-114	Epithelial
KRT6C	0.76	1.14486E-58	Epithelial
LAD1	0.76	2.024E-146	Epithelial
CAPNS2	0.75	4.0669E-113	Epithelial
C2orf54	0.75	1.50455E-97	Epithelial

CLIC3	0.74	2.17986E-81	Epithelial
PRDX1	0.73	1.8279E-99	Epithelial
GLTP	0.73	1.2013E-133	Epithelial
CLTB	0.73	1.3281E-108	Epithelial
EVPL	0.73	2.52113E-94	Epithelial
AIF1L	0.73	7.18316E-25	Epithelial
DEFB1	0.72	2.73636E-96	Epithelial
PRSS22	0.72	1.47446E-42	Epithelial
KLK10	0.72	1.55837E-86	Epithelial
NMU	0.71	1.8419E-107	Epithelial
SPINT1	0.71	3.1754E-107	Epithelial
ALDH3A2	0.70	4.1233E-134	Epithelial
BLVRB	0.70	3.7513E-105	Epithelial
VSNL1	0.70	2.045E-104	Epithelial
TSPO	0.69	2.2916E-175	Epithelial
CXCL17	0.68	9.5985E-100	Epithelial
DMKN	0.65	7.1744E-90	Epithelial
TMPRSS11D	0.65	4.7658E-59	Epithelial
PADI1	0.64	9.71455E-24	Epithelial
ATP5G3	0.64	6.92278E-77	Epithelial
SH3BGRL2	0.64	3.92781E-49	Epithelial
CES2	0.62	2.66635E-95	Epithelial
MPZL2	0.61	3.6748E-161	Epithelial
SCIN	0.61	1.56562E-68	Epithelial
EHF	0.60	3.2361E-116	Epithelial
BARX2	0.60	4.29629E-84	Epithelial
GABRP	0.59	3.85975E-63	Epithelial
TTC9	0.59	8.77226E-51	Epithelial
CAPN1	0.59	1.5098E-115	Epithelial
ATP1B1	0.59	7.85701E-83	Epithelial
PAX9	0.58	4.11143E-96	Epithelial
FAM3B	0.58	2.2519E-67	Epithelial
FAM3D	0.58	2.25723E-33	Epithelial
DUSP5	0.57	0.034264846	Epithelial
PRSS3	0.56	2.82533E-31	Epithelial
DHCR24	0.56	1.582E-103	Epithelial
GRPEL2	0.56	2.5807E-22	Epithelial
CKMT1B	0.55	1.21511E-89	Epithelial
CKMT1A	0.55	1.03803E-96	Epithelial
SDCBP2	0.55	1.28928E-64	Epithelial
PVRL4	0.54	4.85655E-91	Epithelial
TMPRSS11BNL	0.54	8.81017E-69	Epithelial
HMGA1	0.54	5.8604E-108	Epithelial

SULT2B1	0.53	1.61253E-73	Epithelial
SDR16C5	0.53	2.06034E-82	Epithelial
SOX2	0.53	3.12276E-86	Epithelial
SERPINB1	0.53	8.27267E-57	Epithelial
TMPRSS11A	0.53	1.72022E-74	Epithelial
GIPC1	0.53	6.08328E-89	Epithelial
PRSS8	0.52	1.23184E-75	Epithelial
PTPRF	0.52	4.9313E-91	Epithelial
LTA4H	0.52	3.0217E-100	Epithelial
IRF6	0.52	1.48348E-94	Epithelial
COMTD1	0.51	3.8983E-104	Epithelial
S100A7	0.51	1.76755E-14	Epithelial
CTC-276P9.1	0.50	2.60437E-54	Epithelial
PPDPF	0.50	2.44388E-14	Epithelial
STAP2	0.49	6.40612E-97	Epithelial
PTTG1	0.49	1.30204E-82	Epithelial
KLK13	0.49	9.92419E-61	Epithelial
IVL	0.49	2.0799E-56	Epithelial
CXADR	0.49	8.6985E-106	Epithelial
ENO1	0.48	3.47564E-60	Epithelial
TUBB4B	0.48	1.65647E-76	Epithelial
HEBP2	0.48	4.36933E-83	Epithelial
CTNNBIP1	0.48	3.86964E-82	Epithelial
MUC4	0.47	2.3325E-20	Epithelial
ERBB3	0.47	4.1641E-78	Epithelial
TUBA1C	0.47	3.69071E-66	Epithelial
PRRG4	0.47	1.44902E-98	Epithelial
GPT2	0.47	3.37411E-60	Epithelial
GRHL1	0.47	9.08442E-71	Epithelial
SDC4	0.46	2.22209E-79	Epithelial
S100A10	0.46	2.78966E-59	Epithelial
PLIN3	0.46	1.04603E-70	Epithelial
IL20RB	0.46	2.81222E-84	Epithelial
ESRP1	0.46	2.85536E-90	Epithelial
C1orf116	0.45	2.89469E-55	Epithelial
GCHFR	0.45	4.51447E-56	Epithelial
ABLIM1	0.45	3.14915E-79	Epithelial
RAB10	0.45	5.45993E-77	Epithelial
EIF5A	0.45	3.02206E-59	Epithelial
IL18	0.44	7.33146E-23	Epithelial
NAA20	0.44	6.27842E-91	Epithelial
COX6A1	0.44	3.07731E-54	Epithelial
DTX2	0.44	3.25699E-45	Epithelial

TKT	0.44	2.01265E-57	Epithelial
PRDX5	0.44	3.82396E-52	Epithelial
MDH2	0.43	5.50604E-68	Epithelial
C4orf3	0.43	6.75029E-06	Epithelial
DUOX1	0.43	5.28824E-65	Epithelial
EPS8L2	0.43	1.43692E-92	Epithelial
TALDO1	0.43	4.19509E-82	Epithelial
AKR1B10	0.43	8.64207E-40	Epithelial
GJB6	0.43	3.30349E-51	Epithelial
COA3	0.43	1.95261E-64	Epithelial
EPHA2	0.42	4.98956E-40	Epithelial
CDH1	0.42	3.62433E-69	Epithelial
CCDC64B	0.42	7.09568E-57	Epithelial
TGM1	0.42	1.20736E-34	Epithelial
RIOK3	0.42	2.08932E-79	Epithelial
USMG5	0.41	3.49715E-72	Epithelial
NTRK2	0.41	2.89432E-49	Epithelial
SH3YL1	0.41	4.03931E-83	Epithelial
TECR	0.41	7.0349E-67	Epithelial
YOD1	0.41	1.46686E-19	Epithelial
MRPL12	0.40	9.31834E-67	Epithelial
NTS	0.40	8.0887E-06	Epithelial
AHNAK	0.40	1.7839E-56	Epithelial
GGH	0.40	8.29197E-62	Epithelial
TMPRSS4	0.40	3.24495E-71	Epithelial
CAST	0.40	5.06739E-74	Epithelial
ATP5I	0.40	3.93654E-37	Epithelial
EIF6	0.40	4.31606E-65	Epithelial
COX7A2	0.39	4.9387E-66	Epithelial
ANXA2	0.39	2.48132E-80	Epithelial
KRT8	0.39	7.94497E-58	Epithelial
TP63	0.39	7.69886E-60	Epithelial
SLC7A1	0.39	1.39917E-59	Epithelial
PVRL1	0.39	2.06306E-67	Epithelial
ALDH9A1	0.39	5.21256E-65	Epithelial
ANXA3	0.38	1.13992E-51	Epithelial
SPINT2	0.38	1.73225E-80	Epithelial
CEACAM5	0.38	1.72037E-26	Epithelial
H2AFJ	0.38	1.32049E-40	Epithelial
NEAT1	0.38	1.65541E-09	Epithelial
ALDOA	0.37	1.22137E-41	Epithelial
DUOXA1	0.37	1.19147E-64	Epithelial
FUT3	0.37	4.62307E-36	Epithelial

VDAC2	0.37	2.68489E-64	Epithelial
AHCY	0.37	9.0698E-79	Epithelial
SCGB1A1	0.37	1.8286E-15	Epithelial
PTGR1	0.37	3.99418E-63	Epithelial
MPP7	0.37	5.86862E-65	Epithelial
MALL	0.37	1.61811E-18	Epithelial
RIPK4	0.36	3.04332E-55	Epithelial
TRNP1	0.36	3.08307E-32	Epithelial
KRT17	0.36	4.80502E-25	Epithelial
COX8A	0.36	1.2028E-63	Epithelial
KRT7	0.36	2.55241E-26	Epithelial
TMOD3	0.36	1.91431E-48	Epithelial
CD9	0.36	5.54401E-51	Epithelial
RP11-532F12.5	0.36	1.58331E-58	Epithelial
CDH26	0.36	3.03633E-20	Epithelial
TMEM79	0.35	2.52982E-57	Epithelial
RNF141	0.35	1.54573E-73	Epithelial
GPR110	0.35	1.0826E-23	Epithelial
UQCRQ	0.35	1.21689E-39	Epithelial
CBR1	0.35	6.15372E-58	Epithelial
ERBB2	0.35	6.72186E-58	Epithelial
KLK12	0.35	5.80852E-23	Epithelial
STK24	0.35	2.00465E-65	Epithelial
CYC1	0.34	1.55481E-49	Epithelial
VSIG10L	0.34	1.13841E-30	Epithelial
RHOD	0.34	9.77392E-59	Epithelial
CERS3	0.34	4.74216E-56	Epithelial
ZNF750	0.34	3.07612E-54	Epithelial
ATP5A1	0.34	6.13173E-39	Epithelial
ATP5B	0.34	7.73682E-47	Epithelial
SERINC2	0.34	6.11394E-56	Epithelial
GPR87	0.34	1.9587E-70	Epithelial
ALDH3B2	0.34	4.41042E-48	Epithelial
NUDT8	0.34	2.15426E-52	Epithelial
TMEM45B	0.34	2.44792E-39	Epithelial
DDR1	0.34	8.72232E-66	Epithelial
ASPG	0.33	1.65877E-45	Epithelial
IMPA2	0.33	2.23093E-67	Epithelial
CCNB1	0.33	1.98955E-19	Epithelial
CRNDE	0.33	2.88394E-68	Epithelial
COX5B	0.33	7.10409E-35	Epithelial
SLC25A3	0.33	5.03668E-41	Epithelial
TIMM13	0.33	1.40522E-47	Epithelial

PROM2	0.33	8.597E-55	Epithelial
RAPGEFL1	0.32	8.50597E-56	Epithelial
FGFR3	0.32	2.27205E-50	Epithelial
ETFB	0.32	6.80464E-59	Epithelial
SCNN1B	0.32	1.35029E-47	Epithelial
CCL26	0.32	1.02143E-14	Epithelial
ATP5H	0.32	1.39136E-55	Epithelial
DENND2C	0.32	2.18232E-45	Epithelial
ZNF812	0.32	2.64296E-17	Epithelial
MT-CO3	0.31	2.65291E-18	Epithelial
NAPRT1	0.31	1.69927E-70	Epithelial
TMEM54	0.31	7.27151E-63	Epithelial
FAM162A	0.31	3.1757E-50	Epithelial
TRIM16	0.31	2.51343E-64	Epithelial
DBI	0.31	6.0897E-52	Epithelial
FAM83D	0.31	1.50212E-51	Epithelial
SYTL1	0.31	2.3129E-112	Epithelial
MUC15	0.31	3.46274E-44	Epithelial
GJB5	0.31	1.38923E-61	Epithelial
EMP2	0.31	4.86952E-54	Epithelial
UQCRC1	0.31	3.87377E-52	Epithelial
MAPK13	0.31	6.83813E-67	Epithelial
PPIA	0.31	5.33517E-40	Epithelial
AP1M2	0.31	1.09025E-60	Epithelial
TMPRSS2	0.31	7.33634E-17	Epithelial
BNIPL	0.31	3.18451E-47	Epithelial
CYP4B1	0.30	9.68088E-47	Epithelial
TP53AIP1	0.30	4.23912E-39	Epithelial
TIMM8B	0.30	1.27938E-61	Epithelial
C10orf99	0.30	3.52411E-40	Epithelial
P4HB	0.30	6.28671E-40	Epithelial
KCTD1	0.30	9.75514E-60	Epithelial
MYH14	0.30	5.28876E-43	Epithelial
RRM2	0.29	6.26751E-21	Epithelial
LINC01133	0.29	1.88675E-48	Epithelial
HSBP1L1	0.29	5.23911E-66	Epithelial
TRIP10	0.29	5.85632E-30	Epithelial
TMEM184A	0.29	3.08854E-39	Epithelial
CENPW	0.29	1.38696E-31	Epithelial
TPD52L1	0.29	4.28897E-98	Epithelial
TMEM134	0.29	2.95676E-79	Epithelial
RALA	0.29	4.57894E-74	Epithelial
GJB3	0.29	5.52061E-53	Epithelial

SRD5A1	0.29	7.44365E-59	Epithelial
CRB3	0.29	3.26726E-44	Epithelial
EPHX3	0.29	2.81656E-51	Epithelial
ANXA9	0.28	1.50186E-14	Epithelial
COX6B1	0.28	1.37665E-41	Epithelial
SOWAHC	0.28	2.67438E-54	Epithelial
C1QBP	0.28	3.89326E-41	Epithelial
C18orf25	0.28	1.90849E-24	Epithelial
ATP5G1	0.28	4.438E-49	Epithelial
SH3RF2	0.28	1.44596E-45	Epithelial
IDH1	0.28	1.86672E-66	Epithelial
ATP5J2	0.28	1.28399E-48	Epithelial
SPNS2	0.28	1	Epithelial
MTCH2	0.28	3.80111E-55	Epithelial
KRTCAP3	0.28	1.03475E-46	Epithelial
SCNN1A	0.28	3.54433E-42	Epithelial
SMIM5	0.27	9.2811E-39	Epithelial
LAMB3	0.27	1.31939E-27	Epithelial
TTC22	0.27	1.49636E-40	Epithelial
PPP1R14B	0.27	1.72707E-56	Epithelial
GID8	0.27	6.16067E-69	Epithelial
C14orf2	0.27	2.73616E-30	Epithelial
F12	0.27	1.90635E-45	Epithelial
AC004562.1	0.27	6.25298E-20	Epithelial
AKR1C2	0.27	5.46611E-25	Epithelial
TPRG1	0.27	3.76813E-30	Epithelial
C20orf24	0.27	1.18168E-51	Epithelial
NDUFB9	0.26	1.79476E-30	Epithelial
HK1	0.26	1.69298E-59	Epithelial
FAM83H	0.26	1.92744E-48	Epithelial
RAN	0.26	2.88174E-23	Epithelial
COL17A1	0.26	3.13479E-16	Epithelial
GPX2	0.26	7.47746E-36	Epithelial
ARHGAP32	0.26	3.52553E-44	Epithelial
CA2	0.26	1.15407E-15	Epithelial
RHBDL2	0.26	3.16029E-49	Epithelial
SFTA2	0.25	5.64525E-12	Epithelial
KCNK7	0.25	1.73109E-35	Epithelial
SCD	0.25	2.60286E-41	Epithelial
RPLP0	0.25	1.60126E-43	Epithelial
YWHAZ	0.25	2.60411E-95	Epithelial
VPS4B	0.25	1.43143E-58	Epithelial
ATP6V1D	0.25	1.68676E-60	Epithelial

CCDC34	0.25	1.47383E-36	Epithelial
PHLDA3	0.25	7.03691E-47	Epithelial
ATP5O	0.25	3.32445E-29	Epithelial
CCL5	3.39	9.9358E-186	Lymphocyte
GNLY	2.62	5.5608E-43	Lymphocyte
CD3D	2.60	2.063E-195	Lymphocyte
GZMA	2.57	2.1884E-119	Lymphocyte
KLRB1	2.55	1.21724E-88	Lymphocyte
CD2	2.48	3.8599E-180	Lymphocyte
CD7	2.45	3.7341E-168	Lymphocyte
NKG7	1.99	1.7497E-108	Lymphocyte
CD3E	1.98	2.529E-131	Lymphocyte
IFNG	1.88	1.02765E-40	Lymphocyte
IL32	1.78	2.8085E-165	Lymphocyte
CD3G	1.74	1.49312E-84	Lymphocyte
GZMB	1.69	6.71542E-65	Lymphocyte
PTPRCAP	1.64	1.4877E-157	Lymphocyte
LCK	1.51	1.98955E-75	Lymphocyte
CD8A	1.51	4.95003E-57	Lymphocyte
XCL2	1.50	9.54459E-36	Lymphocyte
KLRC1	1.41	7.34028E-31	Lymphocyte
AC092580.4	1.41	2.73124E-49	Lymphocyte
GZMH	1.37	4.29311E-38	Lymphocyte
ZFP36L2	1.36	3.6036E-132	Lymphocyte
XCL1	1.35	2.11838E-23	Lymphocyte
GZMK	1.31	2.43914E-21	Lymphocyte
ZNF683	1.29	5.656E-37	Lymphocyte
CD8B	1.28	5.06444E-41	Lymphocyte
CXCR4	1.16	6.8608E-110	Lymphocyte
KLRD1	1.12	9.75733E-32	Lymphocyte
STK17A	1.11	9.06398E-38	Lymphocyte
BTG1	1.10	1.5919E-209	Lymphocyte
CD96	1.09	1.68993E-40	Lymphocyte
CD247	1.09	2.22506E-37	Lymphocyte
CCL4	1.08	9.04321E-50	Lymphocyte
PPP2R5C	1.08	9.72995E-38	Lymphocyte
GZMM	1.04	1.85929E-34	Lymphocyte
GPR171	1.04	2.86669E-33	Lymphocyte
CD27	1.03	4.53677E-36	Lymphocyte
PRF1	0.99	1.24289E-28	Lymphocyte
SIT1	0.98	1.2641E-33	Lymphocyte
RORA	0.98	3.02349E-24	Lymphocyte
ACAP1	0.96	6.90086E-34	Lymphocyte

PTPRC	0.95	1.44811E-57	Lymphocyte
CXCR6	0.94	8.84389E-28	Lymphocyte
EVL	0.93	1.37017E-69	Lymphocyte
LAG3	0.91	3.27568E-24	Lymphocyte
CITED2	0.89	3.45554E-21	Lymphocyte
TIGIT	0.89	1.16993E-21	Lymphocyte
CST7	0.89	1.90103E-27	Lymphocyte
IL2RB	0.84	3.47656E-23	Lymphocyte
TBC1D10C	0.83	1.11059E-29	Lymphocyte
RGL4	0.83	9.89889E-23	Lymphocyte
APOBEC3G	0.82	1.63403E-19	Lymphocyte
SEPT1	0.82	1.95826E-23	Lymphocyte
ICOS	0.81	7.15584E-20	Lymphocyte
ISG20	0.80	2.65733E-17	Lymphocyte
CLEC2D	0.80	1.57213E-18	Lymphocyte
PYHIN1	0.80	1.91717E-19	Lymphocyte
CD6	0.80	3.42244E-22	Lymphocyte
GATA3	0.79	2.4379E-19	Lymphocyte
RP11-138A9.2	0.75	1.7247E-08	Lymphocyte
PTGER4	0.75	2.14235E-09	Lymphocyte
RARRES3	0.70	1.67323E-14	Lymphocyte
CYTIP	0.68	2.0246E-13	Lymphocyte
BCL11B	0.68	1.25894E-07	Lymphocyte
KIAA1551	0.66	3.54538E-10	Lymphocyte
ZAP70	0.64	2.03094E-14	Lymphocyte
STK17B	0.63	1.83607E-32	Lymphocyte
DUSP2	0.63	2.52134E-23	Lymphocyte
FYB	0.61	1.04236E-10	Lymphocyte
TOB1	0.61	1	Lymphocyte
DUSP4	0.60	3.74268E-09	Lymphocyte
RP11-51J9.5	0.59	2.50021E-11	Lymphocyte
B2M	0.59	6.0142E-150	Lymphocyte
RAB27A	0.58	4.57636E-09	Lymphocyte
LINC00152	0.57	0.000801003	Lymphocyte
STK4	0.57	8.182E-12	Lymphocyte
FAM46C	0.57	2.19956E-12	Lymphocyte
EML4	0.56	0.000100225	Lymphocyte
CCDC107	0.54	0.000211593	Lymphocyte
RP11-138A9.1	0.54	0.000447314	Lymphocyte
FKBP11	0.54	0.044919722	Lymphocyte
SPOCK2	0.53	3.64875E-09	Lymphocyte
CORO1A	0.53	1.62392E-21	Lymphocyte
TTC39C	0.53	1	Lymphocyte

TAGAP	0.53	0.025030844	Lymphocyte
CD48	0.52	4.81873E-06	Lymphocyte
PTPN22	0.52	6.53217E-09	Lymphocyte
PIK3IP1	0.52	4.832E-07	Lymphocyte
RASGRP1	0.51	0.001250313	Lymphocyte
PTPN4	0.51	3.17195E-07	Lymphocyte
CDC42SE2	0.50	0.572892456	Lymphocyte
ANKRD32	0.50	2.74369E-05	Lymphocyte
FNBP1	0.50	0.313900344	Lymphocyte
GNG2	0.50	7.17797E-07	Lymphocyte
PPP1R2	0.49	1	Lymphocyte
RNF213	0.49	1	Lymphocyte
IL7R	0.49	5.0678E-13	Lymphocyte
LEPROTL1	0.49	6.31464E-15	Lymphocyte
RP11-94L15.2	0.47	7.67105E-15	Lymphocyte
SLA	0.47	1	Lymphocyte
SEPT9	0.45	1	Lymphocyte
GYPC	0.45	0.075105013	Lymphocyte
RHOF	0.45	1	Lymphocyte
EMB	0.45	0.765178946	Lymphocyte
LYAR	0.44	1	Lymphocyte
PFKFB3	0.44	1	Lymphocyte
RP11-640M9.1	0.44	0.001269711	Lymphocyte
ICAM3	0.44	0.464183929	Lymphocyte
PARP8	0.43	0.001650707	Lymphocyte
BIN2	0.43	1	Lymphocyte
LTB	0.43	1	Lymphocyte
SRSF7	0.43	1	Lymphocyte
AAK1	0.43	0.006472225	Lymphocyte
BIN1	0.42	0.135826236	Lymphocyte
GABARAPL1	0.42	1	Lymphocyte
PNRC1	0.42	2.64488E-08	Lymphocyte
TNFRSF18	0.42	1	Lymphocyte
BATF	0.41	0.002228536	Lymphocyte
TRAF3IP3	0.41	2.66459E-08	Lymphocyte
ARL4C	0.40	1	Lymphocyte
BUB3	0.40	1	Lymphocyte
LDLRAD4	0.40	1	Lymphocyte
CLDND1	0.39	1	Lymphocyte
SH2D2A	0.39	1.03784E-07	Lymphocyte
PPP1R18	0.39	1	Lymphocyte
PCSK7	0.38	1	Lymphocyte
SMCHD1	0.38	1	Lymphocyte

SUN2	0.37	1	Lymphocyte
TSC22D4	0.37	0.000556946	Lymphocyte
PRDM1	0.37	1	Lymphocyte
ANKRD12	0.37	1	Lymphocyte
DOK2	0.36	1	Lymphocyte
ARHGEF1	0.36	1	Lymphocyte
DENND2D	0.36	2.24656E-07	Lymphocyte
CTSW	0.36	1	Lymphocyte
ZC3HAV1	0.35	1	Lymphocyte
SYTL3	0.35	0.042742159	Lymphocyte
CDKN1B	0.34	1	Lymphocyte
RASAL3	0.34	0.131464571	Lymphocyte
LIMD2	0.34	1	Lymphocyte
AKNA	0.34	1	Lymphocyte
DEF6	0.34	1	Lymphocyte
C9orf78	0.33	1	Lymphocyte
TUBA4A	0.33	2.23596E-12	Lymphocyte
RNF167	0.33	1	Lymphocyte
FAM177A1	0.33	1	Lymphocyte
ARHGAP9	0.33	1	Lymphocyte
RPS29	0.33	4.13705E-30	Lymphocyte
WIPF1	0.32	1	Lymphocyte
TERF2IP	0.32	1	Lymphocyte
PIP4K2A	0.32	0.075687788	Lymphocyte
PRPF38B	0.32	1	Lymphocyte
TBCC	0.31	1	Lymphocyte
TAPSAR1	0.31	1	Lymphocyte
RPS27	0.31	4.23486E-58	Lymphocyte
LNPEP	0.31	1	Lymphocyte
TNFRSF1B	0.31	1	Lymphocyte
PPP1CA	0.31	1	Lymphocyte
MALAT1	0.30	3.1909E-85	Lymphocyte
ID2	0.30	8.1697E-13	Lymphocyte
PIK3R1	0.30	1	Lymphocyte
RBL2	0.30	1	Lymphocyte
APBB1IP	0.30	1	Lymphocyte
FGFR1OP2	0.29	1	Lymphocyte
FAM107B	0.29	1	Lymphocyte
HMHA1	0.29	1	Lymphocyte
TMC6	0.29	1	Lymphocyte
PBXIP1	0.28	1	Lymphocyte
TAPBP	0.28	1	Lymphocyte
ARHGAP30	0.28	1	Lymphocyte

CCDC167	0.27	1	Lymphocyte
ARF6	0.27	7.82932E-06	Lymphocyte
TLK1	0.27	1	Lymphocyte
APOBEC3C	0.27	1	Lymphocyte
SPN	0.27	1	Lymphocyte
IKZF1	0.26	1	Lymphocyte
G3BP2	0.26	1	Lymphocyte
CRIP1	0.26	1	Lymphocyte
MGAT4A	0.26	1	Lymphocyte
MBNL1	0.26	1	Lymphocyte
RP11-349A22.5	0.26	1	Lymphocyte
CCND3	0.25	1	Lymphocyte
IL16	0.25	1	Lymphocyte
CD52	0.25	1.9874E-20	Lymphocyte
HMOX2	0.25	1	Lymphocyte
PRRC2C	0.25	1	Lymphocyte
GMFG	0.25	1	Lymphocyte
LYZ	3.58	1.0398E-122	Monocyte
HLA-DPB1	3.36	1.9344E-226	Monocyte
HLA-DQA1	3.15	6.1355E-200	Monocyte
HLA-DRA	3.14	1.2143E-242	Monocyte
C1QB	2.81	4.26943E-83	Monocyte
C1QA	2.81	8.61835E-88	Monocyte
HLA-DPA1	2.75	1.083E-225	Monocyte
HLA-DQB1	2.62	4.6946E-194	Monocyte
C1QC	2.61	6.31682E-82	Monocyte
AIF1	2.40	1.7821E-152	Monocyte
MS4A6A	2.27	1.3725E-121	Monocyte
IL1B	2.26	8.56476E-69	Monocyte
CD74	2.09	7.1812E-237	Monocyte
HLA-DRB1	2.09	3.0129E-228	Monocyte
S100B	2.01	1.50211E-66	Monocyte
HLA-DRB5	1.87	8.13328E-53	Monocyte
IL8	1.83	6.03691E-40	Monocyte
LST1	1.81	2.5373E-128	Monocyte
CD1E	1.65	1.1789E-63	Monocyte
CLEC10A	1.63	4.22237E-71	Monocyte
RNASE6	1.52	3.11742E-80	Monocyte
HLA-DQA2	1.50	4.31776E-50	Monocyte
HLA-DQB2	1.47	8.65273E-42	Monocyte
FCGBP	1.44	1.05906E-28	Monocyte
HLA-DMA	1.42	1.3708E-139	Monocyte
CD1A	1.38	5.31579E-38	Monocyte

HLA-DMB	1.34	2.5702E-101	Monocyte
CTSS	1.32	1.29544E-73	Monocyte
MNDA	1.32	1.06182E-57	Monocyte
LGALS2	1.32	6.26563E-45	Monocyte
CST3	1.24	1.8339E-43	Monocyte
FGL2	1.24	7.60248E-77	Monocyte
G0S2	1.21	2.00494E-17	Monocyte
CPVL	1.20	2.75012E-56	Monocyte
CD14	1.17	3.61395E-31	Monocyte
DNASE1L3	1.16	4.10837E-24	Monocyte
NPC2	1.10	1.36518E-38	Monocyte
IGSF6	1.10	3.07892E-52	Monocyte
CCL3	1.07	3.88758E-21	Monocyte
LY86	1.06	8.81352E-55	Monocyte
MS4A7	1.06	2.91301E-40	Monocyte
CD86	1.05	2.21221E-51	Monocyte
CSF1R	1.02	1.27301E-49	Monocyte
CTSZ	1.02	7.88123E-43	Monocyte
YWHAH	1.01	6.57919E-37	Monocyte
CD1C	1.00	2.16445E-39	Monocyte
CLEC7A	0.94	1.2095E-46	Monocyte
SERPINA1	0.94	3.25614E-35	Monocyte
MARCH1	0.90	3.88809E-47	Monocyte
FCGR2A	0.88	2.66298E-41	Monocyte
SPI1	0.83	1.05033E-52	Monocyte
FCGR2B	0.83	4.91077E-36	Monocyte
FAM26F	0.83	4.31416E-48	Monocyte
PKIB	0.83	2.65203E-45	Monocyte
CD207	0.82	4.39704E-15	Monocyte
HERPUD1	0.82	4.7212E-47	Monocyte
IER5	0.81	1.56625E-05	Monocyte
HCK	0.80	2.03629E-45	Monocyte
IER3	0.80	1	Monocyte
FCER1A	0.80	0.000125777	Monocyte
MPEG1	0.79	4.5766E-34	Monocyte
ATF3	0.79	7.16543E-30	Monocyte
CLEC4A	0.78	1.94311E-35	Monocyte
FCGR3A	0.77	3.32632E-19	Monocyte
GPX1	0.75	2.1094E-50	Monocyte
CTSB	0.74	1	Monocyte
HLA-DOA	0.70	2.70148E-34	Monocyte
PLEK	0.70	3.95641E-51	Monocyte
IFI30	0.68	1.57391E-31	Monocyte

RB1	0.67	4.99006E-25	Monocyte
OLR1	0.64	7.84272E-20	Monocyte
TUBA1B	0.63	3.72133E-29	Monocyte
IRF8	0.63	5.41712E-19	Monocyte
VMO1	0.63	4.00705E-14	Monocyte
LITAF	0.61	3.39981E-16	Monocyte
TYROBP	0.60	8.54171E-35	Monocyte
ADAM28	0.59	4.15841E-29	Monocyte
CD68	0.58	7.71902E-25	Monocyte
STAB1	0.58	2.97201E-10	Monocyte
ITGB2	0.58	7.61215E-61	Monocyte
FTL	0.57	1.08164E-06	Monocyte
GDI2	0.57	8.85885E-13	Monocyte
PPT1	0.57	6.17944E-17	Monocyte
SPP1	0.57	4.33408E-11	Monocyte
FPR3	0.56	5.37749E-19	Monocyte
CD4	0.56	3.38593E-24	Monocyte
COTL1	0.56	3.60902E-38	Monocyte
CLEC5A	0.55	2.9837E-20	Monocyte
THEMIS2	0.54	5.87876E-22	Monocyte
PHACTR1	0.53	1.4783E-31	Monocyte
ARPC3	0.53	6.05353E-41	Monocyte
RNASET2	0.52	7.22009E-35	Monocyte
FAM49B	0.52	1.56998E-46	Monocyte
SAMHD1	0.52	7.01873E-23	Monocyte
FGD2	0.51	1.66572E-17	Monocyte
TYMP	0.51	5.49459E-08	Monocyte
SOD2	0.51	0.011136958	Monocyte
TGFBI	0.49	1.05704E-05	Monocyte
CECR1	0.49	1.5599E-20	Monocyte
TREM2	0.49	6.72062E-16	Monocyte
GPR183	0.48	1.21697E-11	Monocyte
P2RY13	0.47	1.06132E-20	Monocyte
FAM96A	0.47	1	Monocyte
PTPRE	0.46	3.24836E-19	Monocyte
PLD4	0.44	2.82895E-17	Monocyte
ALDH2	0.44	1	Monocyte
BASP1	0.44	6.55416E-26	Monocyte
CHMP1B	0.43	1	Monocyte
MFSD1	0.42	1	Monocyte
CXCL16	0.42	1.26699E-15	Monocyte
NCF2	0.41	1.54711E-18	Monocyte
GRN	0.40	1	Monocyte

VAMP8	0.40	2.44647E-12	Monocyte
SMCO4	0.40	1.58439E-07	Monocyte
LACC1	0.40	2.74328E-11	Monocyte
SLC7A7	0.39	2.49757E-11	Monocyte
PPIF	0.38	1	Monocyte
CD83	0.38	6.81234E-08	Monocyte
EFHD2	0.38	1	Monocyte
F13A1	0.38	0.355308425	Monocyte
SLC20A1	0.37	5.05783E-10	Monocyte
GCA	0.37	5.90195E-12	Monocyte
SSR1	0.37	1	Monocyte
LAMTOR1	0.37	1	Monocyte
ZNF385A	0.37	1	Monocyte
ATP2B1	0.36	0.004447105	Monocyte
RGS18	0.36	1.13223E-20	Monocyte
ACTR2	0.36	0.001916704	Monocyte
B3GNT5	0.36	1	Monocyte
GLRX	0.36	2.38828E-22	Monocyte
OGFRL1	0.36	1	Monocyte
ATP6V1B2	0.35	1	Monocyte
CYBA	0.35	2.87412E-10	Monocyte
SMIM14	0.35	1	Monocyte
ACTB	0.35	2.02199E-20	Monocyte
AXL	0.35	0.020563317	Monocyte
GATM	0.35	1	Monocyte
LGALS9	0.34	6.7338E-09	Monocyte
ARPC5	0.34	2.89137E-08	Monocyte
SERPINB9	0.34	4.4386E-29	Monocyte
SLC8A1	0.33	9.17722E-09	Monocyte
PYCARD	0.33	0.002439372	Monocyte
FGR	0.33	7.49957E-09	Monocyte
GLIPR1	0.33	1.25041E-40	Monocyte
AP2S1	0.32	1	Monocyte
SIRPA	0.32	3.93416E-13	Monocyte
PLEK2	0.32	1	Monocyte
ADAP2	0.32	5.38244E-15	Monocyte
TNFSF13B	0.32	2.97451E-07	Monocyte
POU2F2	0.32	4.15708E-16	Monocyte
NAIP	0.32	5.0374E-08	Monocyte
HSPA1A	0.31	0.00069813	Monocyte
SLC43A2	0.30	2.62414E-13	Monocyte
ETV5	0.30	5.84713E-08	Monocyte
TPP1	0.30	2.56081E-12	Monocyte

C12orf45	0.30	1	Monocyte
SYK	0.30	1	Monocyte
ADAM19	0.30	2.34271E-24	Monocyte
SYNGR2	0.30	1	Monocyte
PLEKHO1	0.29	3.34293E-11	Monocyte
NAAA	0.29	1	Monocyte
NAMPT	0.29	1	Monocyte
TNFAIP8L2	0.28	1.26701E-18	Monocyte
INSIG1	0.28	0.004101411	Monocyte
VOPP1	0.27	0.173804443	Monocyte
C15orf48	0.27	5.61354E-21	Monocyte
CORO1C	0.27	1	Monocyte
HSPA6	0.27	1.99703E-13	Monocyte
PTPLAD2	0.27	0.000206642	Monocyte
BID	0.26	0.013427587	Monocyte
RPS11	0.26	9.6225E-18	Monocyte
CREBL2	0.26	0.040929166	Monocyte
DSE	0.26	1	Monocyte
ATG3	0.26	1	Monocyte
TMEM97	0.26	1	Monocyte
NFKBIE	0.26	1.46757E-11	Monocyte
RPS3A	0.26	1.04229E-12	Monocyte
RPS24	0.25	5.07816E-14	Monocyte
SSR3	0.25	1	Monocyte
PLEKHB2	0.25	1	Monocyte
SLAMF8	0.25	3.85253E-11	Monocyte
C12orf5	0.25	4.93072E-10	Monocyte
CREG1	0.25	1	Monocyte
CCDC88A	0.25	3.04359E-14	Monocyte
TPSAB1	5.33	0	Mast
CPA3	3.88	2.5272E-296	Mast
CTSG	2.98	7.2441E-147	Mast
HPGDS	2.59	1.7994E-231	Mast
KRT1	2.48	4.8802E-169	Mast
VWA5A	2.18	4.8768E-174	Mast
SLC18A2	2.15	4.2214E-161	Mast
RP11-354E11.2	2.11	8.5675E-151	Mast
NSMCE1	2.08	1.4985E-163	Mast
LTC4S	1.96	7.9779E-163	Mast
RGS13	1.86	2.3528E-124	Mast
GATA2	1.86	2.8389E-143	Mast
HPGD	1.81	6.5726E-159	Mast
UTS2	1.80	1.11175E-47	Mast

KIT	1.70	1.48617E-96	Mast
IL1RL1	1.70	1.2284E-104	Mast
GLUL	1.63	2.6039E-157	Mast
CLU	1.54	5.8497E-182	Mast
SAMSN1	1.51	3.5448E-240	Mast
HDC	1.40	3.32528E-74	Mast
SVOPL	1.27	3.366E-63	Mast
BTG2	1.24	1.3431E-111	Mast
SELK	1.21	2.9848E-100	Mast
DUSP6	1.18	7.32917E-83	Mast
NFKBIA	1.16	6.7906E-123	Mast
LIF	1.15	1.48918E-49	Mast
SOCS1	1.15	2.88369E-92	Mast
C1orf186	1.13	2.21855E-55	Mast
GCSAML	1.13	2.81673E-51	Mast
NCOA4	1.11	8.32358E-58	Mast
SRGN	1.09	3.0042E-224	Mast
AC020571.3	1.08	7.90247E-62	Mast
NFKBIZ	1.07	9.19486E-56	Mast
LEO1	1.05	5.60731E-50	Mast
SLC45A3	1.04	1.23139E-48	Mast
CLNK	1.01	1.66378E-43	Mast
SMYD3	0.99	9.88267E-46	Mast
DTNBP1	0.96	4.97884E-42	Mast
GMPR	0.93	6.76422E-44	Mast
ACOT7	0.92	3.10712E-52	Mast
CNRIP1	0.91	4.98004E-40	Mast
EXD3	0.89	6.89093E-30	Mast
DUSP10	0.84	3.52546E-47	Mast
MS4A2	0.84	2.15833E-35	Mast
FOSB	0.84	2.04033E-65	Mast
CAPG	0.83	1.005E-71	Mast
ABCC4	0.82	3.18815E-30	Mast
PTGS1	0.81	2.18249E-29	Mast
C1orf228	0.80	2.34923E-33	Mast
GALC	0.79	1.49102E-31	Mast
CTD-3203P2.2	0.78	5.73341E-27	Mast
CD69	0.77	3.0214E-102	Mast
ITGA2B	0.76	2.43648E-28	Mast
CATSPER1	0.75	1.30579E-34	Mast
PLIN2	0.75	3.09049E-29	Mast
CD44	0.74	3.71353E-80	Mast
PPP1R15A	0.74	6.50531E-31	Mast

TESPA1	0.73	1.72146E-38	Mast
OSBPL8	0.71	3.64496E-24	Mast
HS3ST1	0.71	2.5233E-13	Mast
RAB27B	0.71	0.011844994	Mast
NFATC1	0.71	6.40441E-11	Mast
CALB2	0.71	6.42695E-30	Mast
ATP6V0A2	0.68	1.93609E-30	Mast
CSF2RB	0.68	1.82452E-22	Mast
ARG2	0.67	9.05657E-19	Mast
GALNT6	0.67	9.78412E-27	Mast
MITF	0.67	5.5115E-19	Mast
CNIH1	0.66	1.05283E-05	Mast
NTRK1	0.66	1.14852E-26	Mast
ELSPBP1	0.66	6.1806E-22	Mast
LMNA	0.65	4.49913E-24	Mast
STAP1	0.65	1.89321E-21	Mast
SLC2A3	0.65	3.66984E-30	Mast
GRAP2	0.65	4.46239E-76	Mast
MANF	0.65	1.79482E-07	Mast
ADAM12	0.64	1.75362E-22	Mast
FOXP1	0.64	7.4689E-28	Mast
CPM	0.63	1.95866E-16	Mast
CMA1	0.62	5.77802E-15	Mast
LMO4	0.61	1	Mast
TWISTNB	0.59	2.7051E-13	Mast
CTNNBL1	0.59	4.336E-18	Mast
KLRG1	0.59	3.2597E-29	Mast
ZC3H12A	0.59	1.60662E-17	Mast
KCNMB1	0.57	0.000114688	Mast
GADD45B	0.57	1.28954E-09	Mast
STXBP6	0.57	2.27097E-18	Mast
CTTNBP2	0.57	1.43911E-13	Mast
BHLHE40	0.57	0.02498901	Mast
CD84	0.56	9.31356E-12	Mast
BIRC3	0.56	1.56949E-22	Mast
PRPSAP2	0.56	4.33625E-09	Mast
IER2	0.56	3.73515E-06	Mast
PLEKHF2	0.55	0.035115078	Mast
KCNH2	0.55	5.26414E-17	Mast
DNAJB9	0.54	3.99479E-16	Mast
TDRD3	0.54	1.50018E-14	Mast
RP11-620J15.3	0.53	5.26618E-10	Mast
AKAP12	0.53	1.3537E-11	Mast

ELL2	0.53	1	Mast
MLPH	0.53	2.21794E-18	Mast
C4orf48	0.53	7.47232E-13	Mast
KDM6B	0.53	3.48691E-05	Mast
GFRA3	0.53	2.03668E-17	Mast
CDK15	0.52	1.35848E-17	Mast
LYL1	0.52	5.02653E-06	Mast
PRDX6	0.52	1.88772E-20	Mast
SLC44A1	0.51	1	Mast
NR4A1	0.51	4.58193E-12	Mast
C10orf128	0.51	3.25854E-08	Mast
ABCB8	0.51	1	Mast
HSPA5	0.51	3.2856E-06	Mast
NR4A2	0.50	9.01992E-18	Mast
NUDT14	0.50	3.17529E-07	Mast
C5orf30	0.50	1.92384E-16	Mast
ALOX5AP	0.49	2.82713E-50	Mast
ARL5B	0.49	1.5923E-05	Mast
RP11-76E17.3	0.49	7.46271E-12	Mast
BMP2K	0.48	1.60337E-06	Mast
MBOAT7	0.48	2.32757E-06	Mast
C7orf73	0.48	1	Mast
ATP6V1F	0.47	8.83649E-05	Mast
TPSG1	0.47	5.77802E-15	Mast
SDF2L1	0.47	0.000490896	Mast
CLCN3	0.47	0.382642031	Mast
THAP2	0.47	1	Mast
CYCS	0.46	1	Mast
RHBDD2	0.46	1	Mast
GPR35	0.45	4.88609E-14	Mast
MAML1	0.45	1.87133E-11	Mast
DNAJC3	0.45	2.01949E-06	Mast
EGR3	0.44	2.6106E-05	Mast
THBS1	0.44	1	Mast
SLC26A2	0.44	1	Mast
BACE2	0.44	2.84637E-05	Mast
SEC61G	0.43	9.00941E-11	Mast
HES1	0.43	1	Mast
FAIM	0.43	0.318161828	Mast
RGS1	0.43	8.02289E-75	Mast
CSF1	0.43	5.15182E-08	Mast
RP11-557H15.4	0.43	5.8642E-13	Mast
SKIL	0.42	0.006389547	Mast

CTSD	0.42	1	Mast
ADRB2	0.42	0.074823	Mast
NFKBID	0.42	0.103279329	Mast
DDIT4	0.42	2.05505E-29	Mast
STARD4	0.42	1.82456E-06	Mast
LAT	0.41	6.65696E-49	Mast
AURKA	0.41	0.000438135	Mast
GPR65	0.41	9.29758E-17	Mast
LPCAT2	0.41	6.21627E-05	Mast
TMEM154	0.41	1	Mast
NEDD9	0.41	0.147315504	Mast
HSD17B12	0.40	1	Mast
SFT2D1	0.40	6.70731E-05	Mast
PCMT1	0.40	1	Mast
IDI1	0.40	1.92405E-16	Mast
TNFAIP8	0.39	2.34128E-10	Mast
RSAD2	0.39	2.52516E-12	Mast
H3F3B	0.39	2.04957E-40	Mast
ALOX5	0.39	0.14342562	Mast
FER	0.39	1.03394E-07	Mast
LAX1	0.39	1.72277E-26	Mast
PLGRKT	0.39	0.269541955	Mast
DUSP1	0.38	1.72327E-08	Mast
P2RY14	0.38	3.97775E-12	Mast
TBPL1	0.37	1.43369E-06	Mast
METTL21A	0.37	0.147995709	Mast
FTH1	0.37	2.60724E-50	Mast
VMP1	0.37	1	Mast
TNFRSF9	0.37	1.00793E-17	Mast
GPX4	0.36	1.81284E-10	Mast
EMR2	0.36	0.000429006	Mast
SVIP	0.36	0.001801776	Mast
MIR24-2	0.36	1.4462E-35	Mast
SLC4A2	0.36	6.79158E-09	Mast
FAM46A	0.36	1	Mast
AHR	0.35	1	Mast
MAFF	0.35	1	Mast
EGR1	0.35	4.84999E-08	Mast
SNX5	0.35	1	Mast
NUTM2A-AS1	0.34	1	Mast
FBP1	0.34	0.115726365	Mast
MAST4	0.34	1	Mast
TNIK	0.34	1.85979E-08	Mast

SPNS1	0.34	1	Mast
LMNB1	0.33	1	Mast
NUCB2	0.33	1	Mast
PTGS2	0.33	1	Mast
MSRA	0.33	1	Mast
NDFIP2	0.33	8.37137E-07	Mast
SEPT2	0.33	0.000848911	Mast
STX3	0.33	0.777183367	Mast
CD37	0.33	9.82904E-27	Mast
FAM110A	0.33	1	Mast
CD22	0.32	2.36218E-08	Mast
STXBP2	0.32	1	Mast
CD33	0.32	0.130792353	Mast
LINC00672	0.32	0.000524172	Mast
CAMLG	0.32	1	Mast
SLC11A1	0.32	1.37482E-06	Mast
PRKAR1A	0.32	0.083629492	Mast
FDX1	0.32	0.019927164	Mast
GNPTAB	0.31	1	Mast
FHL3	0.31	0.001816984	Mast
HSP90B1	0.31	1	Mast
HLTF	0.31	0.004581924	Mast
QPCT	0.31	0.001292313	Mast
ZFP36	0.30	1.33471E-29	Mast
C21orf91	0.30	6.53057E-07	Mast
PHLDA2	0.30	1	Mast
JUNB	0.30	2.87241E-18	Mast
DCXR	0.30	3.5319E-07	Mast
MYADM	0.30	0.001580618	Mast
PLAUR	0.29	1	Mast
SPCS1	0.29	0.002841307	Mast
ZNF331	0.29	0.836508041	Mast
BNIP3L	0.29	1	Mast
POLD4	0.29	1	Mast
RPL36AL	0.29	2.30834E-31	Mast
ATXN10	0.29	1	Mast
XBP1	0.29	1	Mast
FCER1G	0.28	6.99922E-24	Mast
LAT2	0.28	1	Mast
IRS2	0.28	0.363584343	Mast
JUN	0.28	6.09593E-18	Mast
TAF9	0.28	1	Mast
PPP1R15B	0.27	1	Mast

ARHGAP18	0.27	1	Mast
TNFAIP3	0.27	1.0422E-25	Mast
USP48	0.27	1	Mast
PDIA3	0.27	1	Mast
COX17	0.26	1	Mast
FOSL2	0.26	1	Mast
DRAP1	0.26	3.5077E-21	Mast
JAZF1	0.26	0.584820999	Mast
CXXC5	0.26	1	Mast
UBA7	0.26	1	Mast
CCDC28A	0.26	1	Mast
SDCBP	0.26	0.008875505	Mast
S100A4	0.26	1.42106E-45	Mast
MAPK6	0.26	4.2199E-05	Mast
VEGFA	0.26	0.915312851	Mast
CLIC1	0.26	4.48473E-12	Mast
MT1F	0.25	3.40387E-09	Mast
NDUFB2	0.25	1	Mast
NDEL1	0.25	1	Mast
PLVAP	2.94	9.3501E-232	Endothelial
AQP1	2.72	1.4998E-229	Endothelial
RAMP2	2.55	1.0157E-224	Endothelial
DARC	2.20	1.54146E-72	Endothelial
CLDN5	2.14	3.0394E-137	Endothelial
INSR	2.09	1.44057E-82	Endothelial
EMCN	2.08	1.7019E-182	Endothelial
ECSCR	1.87	4.4313E-175	Endothelial
IFI27	1.79	4.4971E-194	Endothelial
ENG	1.75	3.4925E-153	Endothelial
ID3	1.71	7.058E-161	Endothelial
ID1	1.69	9.1114E-135	Endothelial
ELTD1	1.66	1.8323E-139	Endothelial
NPDC1	1.65	3.4315E-141	Endothelial
CRIP2	1.64	3.1786E-161	Endothelial
VWF	1.62	2.7355E-117	Endothelial
GNG11	1.56	1.0458E-198	Endothelial
RAMP3	1.55	1.7036E-125	Endothelial
PODXL	1.50	4.3146E-99	Endothelial
JAM2	1.46	5.5316E-121	Endothelial
AC011526.1	1.42	1.5102E-111	Endothelial
CD34	1.39	1.1113E-114	Endothelial
CYYR1	1.38	1.3362E-111	Endothelial
CLEC14A	1.30	2.7842E-102	Endothelial

TM4SF18	1.29	7.98159E-90	Endothelial
VWA1	1.27	1.05431E-78	Endothelial
SLCO2A1	1.27	2.37748E-85	Endothelial
SOX18	1.22	2.83317E-71	Endothelial
HSPG2	1.18	4.92809E-71	Endothelial
NOSTRIN	1.12	2.18671E-82	Endothelial
ESAM	1.12	6.32819E-93	Endothelial
CALCRL	1.11	8.24529E-73	Endothelial
ITGA6	1.11	1.6234E-103	Endothelial
CD320	1.09	4.23678E-82	Endothelial
KDR	1.09	2.43945E-67	Endothelial
EGFL7	1.08	2.417E-128	Endothelial
RDX	1.07	1.45031E-76	Endothelial
PRSS23	1.05	6.17552E-57	Endothelial
HEG1	1.04	2.52705E-61	Endothelial
FAM167B	1.03	9.29845E-61	Endothelial
ADM5	1.03	3.02654E-57	Endothelial
TXNIP	1.02	2.11404E-96	Endothelial
PCDH17	1.01	2.34743E-62	Endothelial
FLT1	1.00	1.97219E-62	Endothelial
MMRN2	1.00	8.4859E-72	Endothelial
BCAM	1.00	3.93366E-88	Endothelial
TM4SF1	0.99	5.7801E-113	Endothelial
EFNA1	0.98	1.05268E-84	Endothelial
PALMD	0.97	9.54873E-68	Endothelial
ADCY4	0.96	1.49857E-68	Endothelial
CDH5	0.96	2.77857E-65	Endothelial
FKBP1A	0.95	7.4955E-114	Endothelial
HYAL2	0.94	7.14267E-58	Endothelial
ETS2	0.92	1.69921E-67	Endothelial
DUSP23	0.92	4.35217E-49	Endothelial
LIFR	0.92	5.99779E-54	Endothelial
MYCT1	0.91	3.50934E-64	Endothelial
NOTCH4	0.90	6.05469E-54	Endothelial
NRN1	0.90	4.48618E-58	Endothelial
ICAM2	0.90	1.89409E-70	Endothelial
SLC9A3R2	0.89	1.16065E-45	Endothelial
LMO2	0.88	6.04053E-66	Endothelial
IGFBP3	0.86	3.61778E-10	Endothelial
CCDC85B	0.85	1.11276E-87	Endothelial
LMCD1	0.85	9.70914E-59	Endothelial
IL33	0.85	1.14369E-42	Endothelial
CD93	0.85	2.2913E-68	Endothelial

RAB13	0.83	1.37631E-62	Endothelial
CD200	0.82	6.87968E-52	Endothelial
RCAN1	0.82	2.85658E-41	Endothelial
RUNDC3B	0.81	1.91611E-50	Endothelial
ROBO4	0.81	1.21101E-52	Endothelial
COL15A1	0.80	8.31879E-45	Endothelial
TSPAN13	0.80	8.93742E-50	Endothelial
KLF2	0.80	8.51194E-60	Endothelial
PASK	0.79	2.18046E-29	Endothelial
RNASE1	0.79	3.1048E-115	Endothelial
TGFBR2	0.79	4.86301E-47	Endothelial
SOX17	0.78	2.71436E-44	Endothelial
HOXD9	0.77	1.85993E-48	Endothelial
PKP4	0.77	3.90335E-60	Endothelial
ELK3	0.76	2.52343E-50	Endothelial
SERTAD4-AS1	0.76	9.0073E-48	Endothelial
KANK3	0.76	1.38428E-48	Endothelial
SYNPO	0.76	2.92753E-42	Endothelial
APLNR	0.75	9.77934E-45	Endothelial
TSC22D1	0.74	4.13462E-94	Endothelial
THSD7A	0.74	1.08002E-45	Endothelial
ABCG2	0.73	1.61236E-39	Endothelial
SOCS3	0.72	1.50098E-35	Endothelial
CDC37	0.72	2.24558E-52	Endothelial
ADAM15	0.71	1.45237E-51	Endothelial
SRP14	0.71	1.23931E-50	Endothelial
MCTP1	0.70	2.10907E-38	Endothelial
LDB2	0.70	8.32882E-48	Endothelial
RASA4	0.70	1.04192E-39	Endothelial
FAM110D	0.70	4.69272E-46	Endothelial
VAMP5	0.69	4.45231E-46	Endothelial
TSHZ2	0.68	1.42624E-42	Endothelial
SELE	0.68	1.46626E-16	Endothelial
SNCG	0.67	2.25318E-41	Endothelial
LEPR	0.67	9.06401E-41	Endothelial
NFIB	0.66	2.55692E-44	Endothelial
GPR146	0.66	1.09672E-40	Endothelial
STC1	0.65	2.26067E-14	Endothelial
LIMS2	0.65	8.72551E-40	Endothelial
TIE1	0.65	2.64038E-51	Endothelial
ERG	0.65	1.35198E-44	Endothelial
SELP	0.64	9.91126E-25	Endothelial
ABCB1	0.63	1.35539E-34	Endothelial

HOXD8	0.63	8.32331E-39	Endothelial
CCL14	0.63	5.09896E-31	Endothelial
RASIP1	0.63	8.52682E-45	Endothelial
RND1	0.63	3.98496E-27	Endothelial
GIMAP7	0.62	3.8006E-123	Endothelial
PVRL2	0.62	5.01792E-38	Endothelial
SPTBN1	0.62	4.79959E-39	Endothelial
MEOX1	0.62	5.18613E-33	Endothelial
LUZP1	0.61	8.31298E-39	Endothelial
LINC01116	0.60	7.35079E-38	Endothelial
JAG2	0.60	2.72754E-29	Endothelial
PLXND1	0.60	2.22971E-33	Endothelial
EHD4	0.60	5.58878E-46	Endothelial
GIMAP4	0.60	4.35076E-98	Endothelial
ACVRL1	0.60	1.81601E-34	Endothelial
PRCP	0.59	2.27848E-15	Endothelial
IPO11	0.59	5.74274E-41	Endothelial
CTNNAL1	0.59	1.47911E-36	Endothelial
SEPW1	0.59	9.41916E-36	Endothelial
GRB10	0.59	3.4501E-29	Endothelial
RBP7	0.59	0.000610416	Endothelial
THBD	0.58	5.569E-36	Endothelial
FAM84A	0.57	0.043292936	Endothelial
EPAS1	0.57	1.30282E-44	Endothelial
FAM198B	0.57	7.22497E-30	Endothelial
ZNF385D	0.57	8.02669E-32	Endothelial
MAP3K11	0.56	3.70308E-33	Endothelial
TNFAIP1	0.56	1.44597E-34	Endothelial
S1PR1	0.56	6.48122E-33	Endothelial
SSFA2	0.56	6.90839E-33	Endothelial
ARHGEF15	0.56	3.81244E-31	Endothelial
APP	0.56	2.85657E-28	Endothelial
TMEM204	0.56	4.00088E-28	Endothelial
MKL2	0.55	2.12452E-31	Endothelial
SEC14L1	0.55	4.01117E-27	Endothelial
PIM3	0.54	3.43071E-31	Endothelial
SH3BP5	0.54	4.15071E-32	Endothelial
MAGI1	0.54	2.33661E-30	Endothelial
SMAD1	0.54	1.03113E-30	Endothelial
PTPRG	0.54	1.75707E-25	Endothelial
RAPGEF4	0.54	1.65225E-21	Endothelial
CSGALNACT1	0.54	9.82309E-30	Endothelial
PDLIM1	0.54	8.06897E-27	Endothelial

EDN1	0.53	5.09215E-21	Endothelial
PRMT1	0.53	8.30211E-31	Endothelial
BMPR2	0.53	1.03181E-27	Endothelial
C1orf115	0.53	5.9346E-34	Endothelial
PIK3R3	0.53	2.41553E-31	Endothelial
GPR116	0.53	1.47547E-35	Endothelial
TMEM88	0.53	3.42401E-32	Endothelial
UACA	0.53	7.37543E-21	Endothelial
SNHG7	0.52	1.30602E-23	Endothelial
LPAR6	0.51	1.32998E-29	Endothelial
ITGB4	0.51	1.90671E-38	Endothelial
HDAC7	0.51	4.82863E-32	Endothelial
TIMP3	0.51	9.76964E-35	Endothelial
GIMAP6	0.51	6.33098E-64	Endothelial
MCF2L	0.51	2.17334E-30	Endothelial
C16orf80	0.50	2.65481E-25	Endothelial
ITGA5	0.50	5.13375E-26	Endothelial
COL4A1	0.50	6.64201E-20	Endothelial
SORBS2	0.50	2.0046E-26	Endothelial
SPARCL1	0.50	4.9316E-45	Endothelial
LSMD1	0.49	5.75425E-28	Endothelial
TEK	0.49	4.6186E-26	Endothelial
GJA1	0.49	5.40794E-19	Endothelial
KIAA0355	0.49	8.912E-28	Endothelial
PLCB1	0.49	1.20095E-23	Endothelial
CTNNB1	0.48	6.62435E-27	Endothelial
PXN	0.48	1.89434E-27	Endothelial
SHE	0.48	1.44759E-25	Endothelial
NUAK1	0.47	3.60953E-22	Endothelial
GSN	0.47	2.73745E-20	Endothelial
NRP1	0.47	2.26232E-28	Endothelial
RAI14	0.47	1.61181E-21	Endothelial
FAM13C	0.47	2.12608E-25	Endothelial
C4orf32	0.47	1.34915E-27	Endothelial
CPLX1	0.47	1.30775E-24	Endothelial
IGFBP4	0.46	1.30276E-23	Endothelial
APLP2	0.45	3.89357E-23	Endothelial
POLE4	0.45	7.31887E-41	Endothelial
CDA	0.45	1.3129E-38	Endothelial
GNAS	0.45	6.12481E-18	Endothelial
TSPAN12	0.45	1.19157E-24	Endothelial
PTPN14	0.45	2.22663E-24	Endothelial
MATN2	0.45	1.08635E-19	Endothelial

SCARB1	0.45	2.85788E-24	Endothelial
PDE2A	0.44	3.04182E-22	Endothelial
CMTM8	0.44	9.92675E-22	Endothelial
MAPK3	0.43	1.42912E-08	Endothelial
RAPGEF3	0.43	1.29116E-21	Endothelial
VIM	0.43	1.13921E-57	Endothelial
RGCC	0.43	1.10311E-07	Endothelial
IFITM1	0.43	3.01629E-19	Endothelial
FRY	0.43	2.72158E-25	Endothelial
HSPA12B	0.42	1.74229E-21	Endothelial
COL4A2	0.42	5.21949E-17	Endothelial
AKR1C3	0.42	1.51822E-18	Endothelial
MGST2	0.41	1.18412E-36	Endothelial
GIMAP1	0.41	5.82878E-62	Endothelial
GUK1	0.41	9.25754E-36	Endothelial
ENPP2	0.41	3.04955E-09	Endothelial
PINK1	0.41	4.95965E-06	Endothelial
ACE	0.41	3.01932E-20	Endothelial
LAPTM4B	0.41	2.38933E-18	Endothelial
RP11-536O18.1	0.41	1.15431E-18	Endothelial
EDNRB	0.41	1.22433E-08	Endothelial
EFCAB4A	0.41	5.23331E-25	Endothelial
NOV	0.41	4.47351E-14	Endothelial
CEP68	0.41	1.35671E-21	Endothelial
THSD1	0.40	1.84072E-22	Endothelial
GIMAP8	0.40	1.87237E-23	Endothelial
CYB5A	0.40	3.78363E-18	Endothelial
ARRDC3	0.40	1.15802E-10	Endothelial
FKBP9	0.40	2.40031E-20	Endothelial
TMEM173	0.39	2.0975E-22	Endothelial
PTPRB	0.39	3.49126E-48	Endothelial
FGD5	0.39	2.72024E-21	Endothelial
RGL2	0.39	4.15714E-20	Endothelial
PTTG1IP	0.39	7.26545E-22	Endothelial
SLC29A1	0.39	1.23968E-21	Endothelial
CPXM2	0.39	4.08722E-20	Endothelial
KTN1	0.38	5.77773E-23	Endothelial
CDH13	0.38	4.42253E-13	Endothelial
KIAA1462	0.38	6.98484E-20	Endothelial
ICA1	0.38	2.26069E-30	Endothelial
FAM43A	0.37	1.72616E-19	Endothelial
NDST1	0.37	6.02531E-12	Endothelial
MOB2	0.37	3.80928E-06	Endothelial

ADAMTS9	0.37	7.67913E-17	Endothelial
CLDN15	0.37	1.07179E-18	Endothelial
LAMA5	0.37	1.57701E-18	Endothelial
BCL6B	0.37	1.08139E-17	Endothelial
GIMAP5	0.36	2.05529E-65	Endothelial
AFAP1L1	0.36	1.29419E-17	Endothelial
MTUS1	0.36	5.80989E-15	Endothelial
CPNE2	0.36	2.46099E-19	Endothelial
IFITM2	0.35	6.01358E-30	Endothelial
OLFML2A	0.35	2.49363E-15	Endothelial
CMIP	0.35	2.10696E-15	Endothelial
RASGRP3	0.35	1.03859E-10	Endothelial
PLK2	0.35	7.86666E-15	Endothelial
EFNB2	0.35	1.17017E-11	Endothelial
SHC1	0.35	1.51741E-16	Endothelial
RALGAPA2	0.35	6.52452E-22	Endothelial
HSD17B11	0.34	3.61562E-21	Endothelial
ARL15	0.34	2.90472E-12	Endothelial
SLC44A2	0.34	1	Endothelial
GLTSCR2	0.34	4.62008E-11	Endothelial
CFI	0.34	1.18406E-21	Endothelial
FSCN1	0.34	4.08828E-16	Endothelial
IFI44L	0.34	3.32918E-15	Endothelial
PLA2G16	0.34	4.11249E-32	Endothelial
CYTL1	0.34	2.10667E-25	Endothelial
CLIC4	0.33	2.84508E-16	Endothelial
MESDC1	0.33	2.08977E-13	Endothelial
GIPC2	0.33	2.97111E-19	Endothelial
PLSCR1	0.33	1.5073E-13	Endothelial
EPHB4	0.33	2.44498E-15	Endothelial
TBC1D15	0.33	1.56295E-17	Endothelial
LAYN	0.33	1.11392E-13	Endothelial
HECW2	0.33	1.0244E-13	Endothelial
NOVA2	0.33	3.97622E-17	Endothelial
MLEC	0.33	3.66844E-08	Endothelial
LSR	0.33	1	Endothelial
RPS6KA2	0.32	2.50162E-16	Endothelial
MECOM	0.32	1	Endothelial
FAM107A	0.32	1.71939E-16	Endothelial
RHOC	0.32	3.65088E-15	Endothelial
FLI1	0.32	2.54256E-36	Endothelial
SLC3A2	0.32	0.001228368	Endothelial
DYSF	0.32	2.46722E-16	Endothelial

HTRA1	0.32	7.71849E-12	Endothelial
SLC35G2	0.32	3.68159E-15	Endothelial
NUCB1	0.31	2.09881E-17	Endothelial
PTRF	0.31	2.2885E-14	Endothelial
VAMP3	0.31	9.64812E-07	Endothelial
ADAMTSL2	0.31	1.59663E-10	Endothelial
TRIOBP	0.31	1	Endothelial
URM1	0.31	1	Endothelial
MMP15	0.31	2.64694E-19	Endothelial
SLC30A1	0.31	6.1993E-11	Endothelial
ATOX1	0.31	6.05319E-15	Endothelial
FAM101B	0.31	4.03254E-24	Endothelial
C1orf54	0.31	4.52902E-17	Endothelial
DLL4	0.31	9.18159E-14	Endothelial
HLA-E	0.30	8.31271E-43	Endothelial
ATOH8	0.30	8.05432E-14	Endothelial
SCARF1	0.30	3.90869E-10	Endothelial
GRAMD1A	0.30	9.3368E-24	Endothelial
FZD4	0.30	1.47567E-14	Endothelial
GFOD2	0.30	2.96125E-07	Endothelial
COMMD2	0.30	1.0633E-10	Endothelial
MSX1	0.30	1.3067E-13	Endothelial
MRPL17	0.30	1	Endothelial
CFLAR	0.30	2.47476E-11	Endothelial
SLC39A10	0.30	1.5749E-16	Endothelial
CPNE8	0.30	1.0339E-09	Endothelial
CLEC1A	0.30	7.81884E-17	Endothelial
TACR1	0.30	8.24248E-18	Endothelial
LCN6.1	0.30	7.10476E-15	Endothelial
CD151	0.29	5.21392E-18	Endothelial
RILPL2	0.29	1.95716E-12	Endothelial
SEMA6A	0.29	2.87428E-12	Endothelial
PPM1F	0.29	1.40379E-11	Endothelial
UBE2J1	0.29	2.65988E-12	Endothelial
MAPK11	0.28	3.24185E-15	Endothelial
RNF7	0.28	0.143253007	Endothelial
LEF1	0.28	2.73726E-13	Endothelial
RPL3	0.28	1.40897E-50	Endothelial
FAM65A	0.28	4.98168E-13	Endothelial
KIF9	0.28	4.13246E-13	Endothelial
A2M	0.28	2.0308E-21	Endothelial
MYBBP1A	0.28	8.13507E-17	Endothelial
PPFIBP1	0.28	6.08767E-11	Endothelial

CASKIN2	0.28	1.36204E-13	Endothelial
TAOK2	0.28	2.80798E-13	Endothelial
HRH1	0.28	5.54227E-15	Endothelial
ZNF467	0.28	8.13103E-14	Endothelial
ABCD4	0.28	1.10676E-16	Endothelial
NES	0.27	2.85721E-12	Endothelial
SPTAN1	0.27	1	Endothelial
EXOC3L2	0.27	3.30769E-14	Endothelial
EBF3	0.27	4.27088E-14	Endothelial
CRIM1	0.27	6.38617E-12	Endothelial
PCGF2	0.27	6.55672E-14	Endothelial
SLC16A14	0.27	1.17937E-09	Endothelial
TTC28	0.27	2.45987E-08	Endothelial
NOS3	0.27	5.75218E-16	Endothelial
GALNT18	0.27	6.16986E-17	Endothelial
ARHGAP29	0.26	7.49418E-19	Endothelial
TRIM47	0.26	1.28318E-11	Endothelial
HIPK3	0.26	7.64411E-07	Endothelial
HLX	0.26	3.03424E-11	Endothelial
DOCK6	0.26	3.22228E-10	Endothelial
DOCK9	0.26	1	Endothelial
CAV1	0.26	1.70243E-16	Endothelial
PIK3C2A	0.26	1.73764E-15	Endothelial
MIER2	0.26	1.79621E-11	Endothelial
CCM2L	0.26	9.76428E-13	Endothelial
PSMB4	0.26	1	Endothelial
ITIH5	0.26	7.90855E-06	Endothelial
ANGPT2	0.26	0.452929812	Endothelial
ANKRD29	0.26	7.99517E-12	Endothelial
MTIF3	0.26	1	Endothelial
CPD	0.25	1.09245E-10	Endothelial
PLEC	0.25	0.303589401	Endothelial
FIS1	0.25	6.60329E-14	Endothelial
PKN3	0.25	5.43489E-13	Endothelial
LUM	3.84	1.2744E-144	Fibroblast
DCN	3.74	2.1532E-222	Fibroblast
COL3A1	3.50	1.8558E-177	Fibroblast
RGS5	3.46	2.3745E-99	Fibroblast
PTGDS	3.33	7.13566E-82	Fibroblast
APOD	3.23	4.2657E-112	Fibroblast
COL1A2	3.16	2.1359E-186	Fibroblast
FBLN1	2.87	1.5339E-115	Fibroblast
MFAP4	2.79	1.2463E-115	Fibroblast

C1S	2.77	6.394E-182	Fibroblast
COL1A1	2.68	2.3297E-134	Fibroblast
COL6A1	2.64	3.574E-158	Fibroblast
COL6A2	2.61	1.0333E-168	Fibroblast
CPE	2.59	1.4631E-117	Fibroblast
CFD	2.47	0.001779623	Fibroblast
SFRP2	2.43	2.41796E-91	Fibroblast
SOD3	2.32	1.191E-136	Fibroblast
C1R	2.29	4.6013E-147	Fibroblast
FAM162B	2.25	3.68982E-79	Fibroblast
HIGD1B	2.23	1.0125E-57	Fibroblast
RARRES2	2.21	5.8032E-133	Fibroblast
ACTA2	2.20	1.08342E-66	Fibroblast
COL6A3	2.18	1.4185E-104	Fibroblast
CCL11	2.17	3.22321E-52	Fibroblast
PCOLCE	2.13	2.3544E-132	Fibroblast
ABCA8	2.08	2.50084E-88	Fibroblast
TAGLN	1.99	3.71465E-62	Fibroblast
MYL9	1.98	2.27897E-78	Fibroblast
ASPN	1.90	2.97883E-69	Fibroblast
TPM2	1.89	2.3049E-101	Fibroblast
NDUFA4L2	1.81	4.52596E-16	Fibroblast
CTSK	1.78	1.49316E-92	Fibroblast
CCDC80	1.76	1.46551E-93	Fibroblast
CALD1	1.76	3.3402E-184	Fibroblast
COL6A5	1.72	1.66131E-64	Fibroblast
FRZB	1.68	3.8219E-52	Fibroblast
PDGFRB	1.68	2.65259E-71	Fibroblast
COX4I2	1.63	5.25073E-44	Fibroblast
CXCL14	1.60	1.31689E-67	Fibroblast
SERPING1	1.57	4.9003E-110	Fibroblast
FN1	1.54	2.83585E-59	Fibroblast
LHFP	1.54	1.15888E-97	Fibroblast
IGFBP5	1.50	1.397E-37	Fibroblast
DPT	1.45	3.20173E-54	Fibroblast
CYR61	1.42	8.14641E-16	Fibroblast
FXYD1	1.41	1.81517E-75	Fibroblast
SERPINF1	1.37	2.23558E-55	Fibroblast
MFGE8	1.36	3.57422E-64	Fibroblast
PLAC9	1.32	1.62124E-68	Fibroblast
ADH1B	1.31	5.6715E-32	Fibroblast
CLDN11	1.29	1.32172E-53	Fibroblast
LTBP4	1.29	4.38112E-54	Fibroblast

POSTN	1.27	9.59078E-24	Fibroblast
NBL1	1.25	1.87714E-45	Fibroblast
NR2F1	1.21	7.62154E-60	Fibroblast
COL5A1	1.20	8.82926E-59	Fibroblast
WFDC1	1.20	4.69808E-39	Fibroblast
MMP2	1.20	4.19629E-42	Fibroblast
MEG3	1.19	3.51547E-53	Fibroblast
TPM1	1.16	5.77575E-14	Fibroblast
THY1	1.16	3.23825E-72	Fibroblast
COL5A2	1.15	1.22372E-58	Fibroblast
TFPI	1.14	2.30725E-64	Fibroblast
PLEKHH2	1.14	2.30169E-47	Fibroblast
RERG	1.13	3.07115E-59	Fibroblast
PTN	1.12	1.7222E-10	Fibroblast
IGFBP6	1.11	1.68461E-48	Fibroblast
SELM	1.11	7.29621E-66	Fibroblast
MEST	1.11	7.40119E-38	Fibroblast
SMOC2	1.09	8.2632E-36	Fibroblast
AEBP1	1.08	3.24714E-66	Fibroblast
PLXDC1	1.07	1.36427E-60	Fibroblast
PDGFRA	1.07	2.4203E-51	Fibroblast
CSRP2	1.07	1	Fibroblast
NOTCH3	1.06	1	Fibroblast
TDO2	1.06	1.98712E-37	Fibroblast
PPP1R14A	1.05	2.22471E-23	Fibroblast
LGALS3BP	1.02	4.58484E-31	Fibroblast
CLEC11A	1.00	1.91403E-32	Fibroblast
NDN	0.99	1.61323E-42	Fibroblast
NR2F2	0.99	8.14324E-31	Fibroblast
COL12A1	0.99	4.21072E-37	Fibroblast
SEPP1	0.96	5.34889E-34	Fibroblast
SDC2	0.93	1.49343E-40	Fibroblast
TPPP3	0.93	4.28969E-33	Fibroblast
F10	0.92	5.25073E-44	Fibroblast
SGCE	0.91	2.21288E-39	Fibroblast
EMILIN1	0.91	1.63006E-41	Fibroblast
ISYNA1	0.91	7.72072E-19	Fibroblast
COL14A1	0.90	4.21073E-37	Fibroblast
TNC	0.90	1.75239E-24	Fibroblast
OGN	0.89	8.63576E-25	Fibroblast
SPON2	0.89	3.30512E-23	Fibroblast
PPAP2A	0.89	6.89498E-28	Fibroblast
PLEKHA5	0.89	1.23482E-14	Fibroblast

CDH6	0.88	8.63576E-25	Fibroblast
ISLR	0.88	5.16547E-42	Fibroblast
APCDD1	0.88	1.26123E-22	Fibroblast
PLTP	0.88	2.66783E-25	Fibroblast
TNFRSF21	0.88	3.33496E-31	Fibroblast
TCF21	0.87	2.21288E-39	Fibroblast
FMO1	0.87	1.19994E-22	Fibroblast
EFEMP2	0.86	8.0882E-47	Fibroblast
FXYD6	0.86	4.61929E-40	Fibroblast
MXRA8	0.86	8.87595E-37	Fibroblast
TIMP2	0.85	4.70846E-23	Fibroblast
NKD2	0.85	2.11109E-38	Fibroblast
CRISPLD2	0.83	3.06332E-39	Fibroblast
CCDC102B	0.83	9.07486E-22	Fibroblast
ANTXR1	0.82	8.52317E-36	Fibroblast
MOXD1	0.82	2.49181E-31	Fibroblast
CHN1	0.81	1.01622E-20	Fibroblast
EDNRA	0.80	3.09457E-33	Fibroblast
THBS2	0.80	3.3365E-34	Fibroblast
DKK3	0.79	8.30797E-40	Fibroblast
STEAP4	0.79	6.39301E-20	Fibroblast
ABI3BP	0.78	1.04421E-30	Fibroblast
SEPT4	0.78	3.08029E-28	Fibroblast
CYGB	0.78	3.02939E-16	Fibroblast
FMOD	0.77	1.49319E-33	Fibroblast
C1QTNF1	0.77	4.25172E-25	Fibroblast
PMP22	0.76	1.7254E-15	Fibroblast
NUPR1	0.75	1.40967E-11	Fibroblast
PRRX1	0.74	1.21775E-31	Fibroblast
OSR2	0.74	2.90379E-27	Fibroblast
RP11-14N7.2	0.73	1.05375E-11	Fibroblast
MMP23B	0.73	6.52692E-33	Fibroblast
ABCA6	0.73	1.71279E-22	Fibroblast
C3	0.72	5.05026E-06	Fibroblast
CYBRD1	0.72	3.06832E-18	Fibroblast
BMP4	0.72	6.96972E-34	Fibroblast
PCDH18	0.72	6.92649E-28	Fibroblast
VCAM1	0.71	2.08914E-26	Fibroblast
IL34	0.71	1.09533E-11	Fibroblast
SYNPO2	0.70	2.04637E-24	Fibroblast
LPPR4	0.70	5.93705E-27	Fibroblast
DDR2	0.69	1.21334E-31	Fibroblast
RASD1	0.69	2.30977E-20	Fibroblast

LRP1	0.69	4.75407E-15	Fibroblast
ADIRF	0.69	6.00429E-05	Fibroblast
RBP1	0.68	1	Fibroblast
LAMA4	0.68	3.12164E-13	Fibroblast
SELENBP1	0.67	1.18088E-05	Fibroblast
MT1M	0.66	8.44819E-23	Fibroblast
SEMA5A	0.66	3.67163E-13	Fibroblast
DIO2	0.65	4.99106E-07	Fibroblast
LGALS1	0.65	1.16352E-52	Fibroblast
COL18A1	0.65	7.56703E-12	Fibroblast
SERPINE2	0.64	0.000656116	Fibroblast
CRYAB	0.64	1	Fibroblast
MLTK	0.63	1.0644E-08	Fibroblast
LAPTM4A	0.63	6.49401E-44	Fibroblast
KANK2	0.62	2.61565E-24	Fibroblast
F2R	0.62	4.32511E-13	Fibroblast
LBH	0.61	1.63795E-26	Fibroblast
ZNF503	0.61	6.04227E-06	Fibroblast
GUCY1A3	0.61	6.45572E-16	Fibroblast
SERPINI1	0.61	1	Fibroblast
FGFR1	0.60	3.364E-14	Fibroblast
NFATC4	0.60	9.80714E-22	Fibroblast
TMEM98	0.60	1	Fibroblast
GUCY1B3	0.59	7.11733E-13	Fibroblast
TMEM176B	0.59	4.05956E-09	Fibroblast
GSTM3	0.58	1	Fibroblast
CDH11	0.58	1.21272E-26	Fibroblast
FBN1	0.58	1.70408E-29	Fibroblast
GEM	0.57	8.05975E-08	Fibroblast
CBX6	0.56	0.009344946	Fibroblast
IGFBP2	0.56	1	Fibroblast
MDK	0.56	1	Fibroblast
VCAN	0.56	1.15787E-21	Fibroblast
FGF7	0.56	1.47569E-19	Fibroblast
ID4	0.56	1	Fibroblast
MRVI1	0.56	1.54039E-18	Fibroblast
FMO3	0.56	2.47E-16	Fibroblast
GPNMB	0.55	0.047006953	Fibroblast
SPATS2L	0.55	1	Fibroblast
SEPT7	0.55	1.11241E-18	Fibroblast
ECM2	0.55	2.55461E-19	Fibroblast
TGFB1I1	0.54	5.62752E-06	Fibroblast
PRSS12	0.54	5.53916E-22	Fibroblast

MYLK	0.54	0.529893419	Fibroblast
GPR124	0.53	1.02282E-24	Fibroblast
PTK7	0.53	0.001047708	Fibroblast
VSTM4	0.53	2.55461E-19	Fibroblast
FBLN2	0.53	3.76346E-20	Fibroblast
MRGPRF	0.53	6.39301E-20	Fibroblast
FBLN5	0.53	4.47027E-25	Fibroblast
MAP1B	0.52	0.250531544	Fibroblast
PTPRS	0.52	1	Fibroblast
PDE5A	0.52	3.19388E-20	Fibroblast
LOXL1	0.52	2.41927E-22	Fibroblast
SLITRK6	0.52	1.27852E-19	Fibroblast
RP11-572C15.6	0.51	1.24692E-16	Fibroblast
FBLIM1	0.51	7.9319E-08	Fibroblast
GAS6	0.50	1	Fibroblast
WLS	0.50	1	Fibroblast
RCN3	0.50	4.18003E-23	Fibroblast
RUNX1T1	0.50	0.193568503	Fibroblast
TMEM45A	0.50	1.75717E-22	Fibroblast
RNASE4	0.50	2.84578E-13	Fibroblast
APOE	0.49	1	Fibroblast
NID1	0.49	2.43333E-10	Fibroblast
SSPN	0.49	1.76892E-09	Fibroblast
PHPT1	0.49	1	Fibroblast
NENF	0.49	0.060062207	Fibroblast
LURAP1L	0.48	3.66651E-20	Fibroblast
FHL2	0.48	1	Fibroblast
NOVA1	0.48	2.96026E-14	Fibroblast
PGF	0.48	1.60429E-13	Fibroblast
PALLD	0.48	1	Fibroblast
TINAGL1	0.48	1	Fibroblast
CCL2	0.48	0.076142343	Fibroblast
PODN	0.48	6.2894E-17	Fibroblast
PTEN	0.48	1	Fibroblast
PKIG	0.47	1	Fibroblast
EDIL3	0.47	0.005165082	Fibroblast
ADH5	0.47	1	Fibroblast
ZCCHC24	0.47	3.71957E-10	Fibroblast
C2	0.47	2.05937E-11	Fibroblast
GSTM5	0.47	3.16965E-17	Fibroblast
TGM2	0.47	1.04138E-09	Fibroblast
ADRA2A	0.46	7.24133E-09	Fibroblast
PAG1	0.46	2.59502E-10	Fibroblast

FOXF2	0.46	1.24692E-16	Fibroblast
PTK2	0.46	1	Fibroblast
MRC2	0.46	1	Fibroblast
C1orf21	0.46	1	Fibroblast
RBMS3	0.46	7.86588E-05	Fibroblast
LSAMP	0.45	3.16965E-17	Fibroblast
IFI27L2	0.45	1	Fibroblast
GFRA1	0.45	2.02715E-18	Fibroblast
HCFC1R1	0.45	1	Fibroblast
SSBP2	0.44	1	Fibroblast
EPHX1	0.44	1	Fibroblast
PAM	0.44	0.055541946	Fibroblast
IL15RA	0.44	2.97977E-11	Fibroblast
P4HA2	0.44	1	Fibroblast
EPB41L2	0.44	6.5276E-07	Fibroblast
RCAN2	0.43	1	Fibroblast
ADCY3	0.43	9.76664E-09	Fibroblast
TMEM176A	0.43	0.000442301	Fibroblast
PFN2	0.43	0.987308615	Fibroblast
FILIP1L	0.43	5.82169E-06	Fibroblast
GREM2	0.43	8.02969E-18	Fibroblast
SCPEP1	0.42	1	Fibroblast
RRBP1	0.42	1	Fibroblast
FSTL1	0.42	0.955434237	Fibroblast
SH3D19	0.42	1	Fibroblast
RNF150	0.42	9.66764E-16	Fibroblast
REV3L	0.42	0.005656279	Fibroblast
CPQ	0.41	0.00047134	Fibroblast
OLFML1	0.41	1.59603E-17	Fibroblast
LTBP1	0.41	1.01554E-13	Fibroblast
NTM	0.41	1.5942E-20	Fibroblast
TNXB	0.41	1	Fibroblast
FKBP7	0.41	1	Fibroblast
PDLIM3	0.41	7.94357E-05	Fibroblast
SKP1	0.41	1.25423E-11	Fibroblast
WNT5A	0.41	1	Fibroblast
CTSF	0.40	0.113936918	Fibroblast
PID1	0.40	1.19225E-09	Fibroblast
C12orf57	0.40	0.005853205	Fibroblast
MYO1B	0.40	1	Fibroblast
PMEPA1	0.40	1.79601E-11	Fibroblast
C10orf10	0.39	1	Fibroblast
CALM2	0.39	3.43374E-16	Fibroblast

LOXL2	0.39	0.134214576	Fibroblast
HOXA3	0.39	1.0709E-10	Fibroblast
ST5	0.39	1	Fibroblast
ARPC1A	0.38	1	Fibroblast
TWSG1	0.38	1.70047E-07	Fibroblast
PTOV1	0.38	1	Fibroblast
UBA2	0.38	1	Fibroblast
GPX3	0.37	1	Fibroblast
ITGB1	0.37	0.014020394	Fibroblast
KITLG	0.37	1	Fibroblast
RAB34	0.37	1	Fibroblast
TPBG	0.37	0.103893521	Fibroblast
RP11-553L6.5	0.37	1	Fibroblast
RBP5	0.37	1	Fibroblast
PAMR1	0.37	6.2894E-17	Fibroblast
C1QTNF2	0.37	4.6118E-06	Fibroblast
PRR24	0.37	1.37537E-06	Fibroblast
SNHG18	0.37	1	Fibroblast
SMTN	0.37	0.000106304	Fibroblast
GULP1	0.37	1	Fibroblast
RP11-834C11.4	0.36	0.000324851	Fibroblast
LAMP1	0.36	1	Fibroblast
TTC3	0.36	1	Fibroblast
ARHGAP12	0.36	1	Fibroblast
EHD2	0.36	1	Fibroblast
SAT2	0.36	1	Fibroblast
SGCB	0.36	1.27127E-05	Fibroblast
HNMT	0.36	0.108736213	Fibroblast
CERCAM	0.35	1.16649E-10	Fibroblast
SPRY1	0.35	1	Fibroblast
TUSC3	0.35	1	Fibroblast
SNAI2	0.35	1	Fibroblast
RHOB	0.34	1	Fibroblast
SGIP1	0.34	0.41015116	Fibroblast
SEC31A	0.34	1	Fibroblast
GLT8D2	0.34	0.001546911	Fibroblast
KCNQ1OT1	0.34	1	Fibroblast
C20orf27	0.33	1	Fibroblast
PPIB	0.33	1	Fibroblast
IRF2BPL	0.33	1	Fibroblast
EBF1	0.33	1	Fibroblast
S100A6	0.33	1.46161E-09	Fibroblast
C5orf15	0.33	1	Fibroblast

PTMS	0.33	1	Fibroblast
KLHL42	0.33	1	Fibroblast
CHD9	0.33	1	Fibroblast
RRAS	0.32	1	Fibroblast
ARID5A	0.32	1	Fibroblast
PPP1R12A	0.32	1	Fibroblast
PIGT	0.32	1	Fibroblast
FOXF1	0.32	0.000106285	Fibroblast
MAP1LC3A	0.32	1	Fibroblast
ZBTB20	0.32	1	Fibroblast
HOXB-AS1	0.32	0.600778722	Fibroblast
LTBP3	0.31	1	Fibroblast
FKBP10	0.31	1	Fibroblast
TNS1	0.31	1	Fibroblast
GNAI1	0.31	1	Fibroblast
OLFML3	0.31	0.027246456	Fibroblast
FAM114A1	0.31	1	Fibroblast
GSTM4	0.31	0.561000802	Fibroblast
SPARC	0.31	2.85682E-06	Fibroblast
FARP1	0.31	1	Fibroblast
PLD3	0.31	0.128702054	Fibroblast
SOD1	0.31	0.468686523	Fibroblast
GPX8	0.31	1	Fibroblast
BST2	0.31	0.50736153	Fibroblast
ESD	0.31	1	Fibroblast
CD59	0.30	1	Fibroblast
FAM92A1	0.30	0.181987421	Fibroblast
NUCKS1	0.30	1	Fibroblast
TPST1	0.30	0.22171985	Fibroblast
COX7A1	0.30	1	Fibroblast
ARF4	0.30	1	Fibroblast
CXCL12	0.30	1	Fibroblast
PARVA	0.29	1	Fibroblast
PPIC	0.29	1	Fibroblast
TMEM59	0.29	0.068857555	Fibroblast
IL1R1	0.29	1	Fibroblast
LAMB2	0.29	1	Fibroblast
COPS6	0.29	1	Fibroblast
CTSL	0.29	1	Fibroblast
PEBP1	0.29	1	Fibroblast
FERMT2	0.29	1	Fibroblast
C1orf122	0.28	1	Fibroblast
CISD1	0.28	1	Fibroblast

REEP3	0.28	1	Fibroblast
PRNP	0.28	1	Fibroblast
ADD3	0.28	1	Fibroblast
MGP	0.27	1	Fibroblast
ROBO1	0.27	1	Fibroblast
PTP4A3	0.27	1	Fibroblast
LRPAP1	0.27	1	Fibroblast
LY6E	0.27	1	Fibroblast
FAP	0.27	1	Fibroblast
OAF	0.27	1	Fibroblast
GADD45A	0.27	1	Fibroblast
CNBP	0.27	1	Fibroblast
MANBAL	0.27	1	Fibroblast
NFIC	0.26	1	Fibroblast
CASC4	0.26	1	Fibroblast
EMC2	0.26	1	Fibroblast
CFH	0.26	1	Fibroblast
EID1	0.26	0.685160755	Fibroblast
MAN1A1	0.26	1	Fibroblast
SEC63	0.26	1	Fibroblast
EFHC1	0.26	1	Fibroblast
AK3	0.26	1	Fibroblast
C1orf86	0.25	1	Fibroblast
PCYOX1	0.25	1	Fibroblast
