# Supplementary material for: Single-cell RNA-Seq of human esophageal epithelium in homeostasis and allergic inflammation
Source: JCI Insight. 2022 Jun 8;7(11):e159093. doi: 10.1172/jci.insight.159093 (PMC9208762; doi:10.1172/jci.insight.159093)
Supplement: Supplemental table 2 [file jciinsight-7-159093-s300.pdf]

Supplemental Table 2. Markers of the human esophageal epithelial subpopulations

gene	logfc_min	adjp_max	epithelial subpopulation
DST	0.92	7.48997E-49	Quiescent
DLK2	0.85	3.47075E-39	Quiescent
KRT15	0.84	1.32928E-65	Quiescent
IL1R2	0.75	1.33512E-25	Quiescent
TXNIP	0.75	1.87494E-28	Quiescent
GPNMB	0.74	2.77492E-33	Quiescent
WNT10A	0.71	1.4448E-28	Quiescent
TSLP	0.70	3.16207E-22	Quiescent
ZFP36L2	0.70	9.44526E-44	Quiescent
GLTSCR2	0.69	1.16263E-51	Quiescent
MIR205HG	0.68	9.93853E-47	Quiescent
ZFAS1	0.68	3.59398E-53	Quiescent
CXCL14	0.67	4.85406E-06	Quiescent
FOS	0.66	1	Quiescent
IFITM3	0.64	3.04676E-35	Quiescent
RPL34	0.63	1.70876E-94	Quiescent
SNHG8	0.62	7.59164E-35	Quiescent
EIF3E	0.59	2.96091E-55	Quiescent
LAMB3	0.59	1.22126E-16	Quiescent
CIRBP	0.59	5.07074E-31	Quiescent
IGFBP5	0.58	0.000491458	Quiescent
APP	0.57	4.57556E-22	Quiescent
EEF2	0.57	1.62148E-52	Quiescent
RPL12	0.57	6.66966E-82	Quiescent
BCAM	0.57	1.3959E-19	Quiescent
PNRC1	0.57	4.02995E-17	Quiescent
EPB41L4A-AS1	0.56	1.81417E-17	Quiescent
SNHG7	0.56	1.52609E-15	Quiescent
RPL13	0.55	9.29588E-94	Quiescent
EFEMP1	0.55	3.21601E-13	Quiescent
RPS27	0.54	1.57924E-80	Quiescent
ASS1	0.53	5.18492E-17	Quiescent
RPL17	0.53	1.1738E-26	Quiescent
FST	0.52	6.06752E-08	Quiescent
COL17A1	0.51	1.24156E-14	Quiescent
CAV1	0.51	8.19134E-17	Quiescent
SLC3A2	0.51	2.09646E-16	Quiescent

TSC22D1	0.50	3.7541E-05	Quiescent
DDIT4	0.50	2.15327E-06	Quiescent
CH25H	0.50	9.94118E-11	Quiescent
GNB2L1	0.49	1.46291E-71	Quiescent
PPP1R16A	0.49	1.14133E-12	Quiescent
RPL31	0.48	3.35905E-74	Quiescent
ETS2	0.48	1.05861E-09	Quiescent
RPL35A	0.48	1.15094E-85	Quiescent
EIF4A2	0.47	3.12024E-25	Quiescent
PFDN5	0.47	5.17922E-38	Quiescent
RPL30	0.46	1.96175E-69	Quiescent
DLL1	0.45	1.48027E-14	Quiescent
FHL2	0.45	1.47008E-10	Quiescent
SLC7A5	0.45	3.13913E-10	Quiescent
RPL13A	0.44	2.70841E-80	Quiescent
PHY^{HIGH}P	0.44	1.79519E-12	Quiescent
RPS9	0.43	3.36622E-71	Quiescent
SAT2	0.43	5.42766E-10	Quiescent
RPL3	0.43	2.96982E-68	Quiescent
RPS19	0.43	7.64202E-76	Quiescent
RPL5	0.42	6.26793E-62	Quiescent
BTF3	0.42	2.96688E-56	Quiescent
RSL24D1	0.42	1.67186E-16	Quiescent
COMMD6	0.42	9.00622E-28	Quiescent
EIF2A	0.41	1.34121E-11	Quiescent
NOB1	0.41	5.28707E-08	Quiescent
C1R	0.41	6.6278E-07	Quiescent
KRT32	0.41	3.88995E-06	Quiescent
PTRF	0.41	1.26335E-08	Quiescent
SLC1A3	0.41	0.008117268	Quiescent
RPL10A	0.40	8.55871E-72	Quiescent
ST13	0.39	5.05953E-17	Quiescent
EEF1D	0.39	1.32929E-32	Quiescent
C6orf48	0.39	3.1084E-08	Quiescent
ATP1B3	0.39	2.51318E-19	Quiescent
WNT5B	0.39	5.99296E-07	Quiescent
CCDC3	0.39	6.32005E-07	Quiescent
Z83851.1	0.39	1.3287E-05	Quiescent
RSL1D1	0.39	5.93653E-12	Quiescent
PRNP	0.39	1.66805E-07	Quiescent
DHRS3	0.39	4.6796E-08	Quiescent
HSPA2	0.38	2.35567E-06	Quiescent
BLCAP	0.38	0.001426013	Quiescent

THSD4	0.38	0.00775339	Quiescent
TAX1BP3	0.38	5.5822E-17	Quiescent
LIMA1	0.37	0.009687125	Quiescent
JUN	0.37	6.17868E-08	Quiescent
TP53AIP1	0.37	4.29868E-10	Quiescent
KLK5	0.37	5.40929E-05	Quiescent
RPL32	0.37	2.33896E-72	Quiescent
RPL36	0.37	5.59493E-66	Quiescent
RPLP1	0.36	5.24859E-67	Quiescent
RPL11	0.36	1.03682E-69	Quiescent
RPL21	0.36	7.81747E-70	Quiescent
EEF1A1	0.36	2.74736E-49	Quiescent
ARPC1B	0.36	1.81962E-06	Quiescent
GAMT	0.36	0.000485216	Quiescent
NME3	0.36	6.04173E-06	Quiescent
CDH13	0.36	4.81752E-05	Quiescent
RPL37	0.35	4.60744E-40	Quiescent
IP6K2	0.35	0.005519206	Quiescent
RIN2	0.35	1	Quiescent
RPS5	0.35	3.79718E-53	Quiescent
RPL15	0.35	2.94012E-61	Quiescent
RPS18	0.35	6.62346E-76	Quiescent
DKK3	0.35	0.000477722	Quiescent
RPL7	0.35	6.97894E-58	Quiescent
RPS28	0.34	6.43676E-56	Quiescent
RPS20	0.34	1.06148E-44	Quiescent
FAM132A	0.34	0.004682244	Quiescent
RPS15A	0.34	3.02566E-61	Quiescent
RPL18A	0.34	1.61277E-61	Quiescent
LMO4	0.34	0.013369467	Quiescent
COL18A1	0.34	0.000303439	Quiescent
N4BP2L2	0.33	4.72305E-08	Quiescent
GPX4	0.33	2.76469E-16	Quiescent
PTPRS	0.33	1	Quiescent
RPS6	0.33	8.48526E-60	Quiescent
RPS8	0.33	9.91581E-56	Quiescent
BTG1	0.33	0.149421146	Quiescent
RPL9	0.32	2.47731E-49	Quiescent
NBEAL1	0.32	0.037573443	Quiescent
EIF3L	0.32	3.03949E-14	Quiescent
RPL38	0.32	2.9322E-34	Quiescent
RPS15	0.32	1.55097E-66	Quiescent
WNT7B	0.31	0.001243749	Quiescent

CCDC80	0.31	1.33174E-06	Quiescent
NACA	0.31	5.40818E-40	Quiescent
MOXD1	0.31	0.013818295	Quiescent
IRF1	0.31	1	Quiescent
SLC40A1	0.31	0.472414631	Quiescent
RPS12	0.30	2.81877E-47	Quiescent
RPS25	0.30	2.0571E-52	Quiescent
AKAP9	0.30	0.315277852	Quiescent
RPL18	0.30	5.42634E-52	Quiescent
PDLIM1	0.30	2.4791E-07	Quiescent
KMT2E	0.29	1	Quiescent
MUM1	0.29	1	Quiescent
RPL41	0.29	1.46028E-59	Quiescent
RARRES3	0.29	0.118962006	Quiescent
EIF3D	0.29	6.69354E-07	Quiescent
RPL14	0.29	2.21657E-42	Quiescent
RPS27A	0.29	8.52334E-54	Quiescent
CD74	0.29	1	Quiescent
RAMP1	0.29	1.38389E-05	Quiescent
RPL23A	0.29	9.51275E-44	Quiescent
KTN1	0.29	5.60723E-06	Quiescent
GPX1	0.29	1.66762E-06	Quiescent
RPS13	0.28	1.32047E-45	Quiescent
EEF1G	0.28	1	Quiescent
RPS7	0.28	2.90589E-42	Quiescent
RPL23	0.28	4.45955E-36	Quiescent
NUDT14	0.28	1	Quiescent
TOMM7	0.28	3.5231E-19	Quiescent
RPS14	0.28	3.0011E-49	Quiescent
SNAI2	0.28	1	Quiescent
CYB5R1	0.28	1	Quiescent
RPL6	0.28	2.07339E-41	Quiescent
NAP1L1	0.27	3.31482E-09	Quiescent
NPR3	0.27	1	Quiescent
BTG2	0.27	1	Quiescent
RPL29	0.27	6.67842E-43	Quiescent
CTSH	0.27	1	Quiescent
ABCC3	0.27	1	Quiescent
NSRP1	0.27	1	Quiescent
VAMP5	0.27	1	Quiescent
RPLP0	0.27	1.53877E-34	Quiescent
HCFC1R1	0.26	1	Quiescent
ORAI3	0.26	1	Quiescent

RPL24	0.26	2.21919E-33	Quiescent
EIF3H	0.26	1.415E-08	Quiescent
BOC	0.26	0.03879264	Quiescent
ASCL2	0.26	0.065813538	Quiescent
NTF4	0.26	1	Quiescent
C1S	0.25	0.00077026	Quiescent
HOOK2	0.25	1	Quiescent
RIN1	0.25	1	Quiescent
HMGB2	1.78	2.4695E-116	Proliferating
H2AFZ	1.69	8.31E-102	Proliferating
TUBA1B	1.52	5.32919E-85	Proliferating
ST1H4C	1.45	1.84075E-39	Proliferating
STMN1	1.43	3.83469E-94	Proliferating
UBE2C	1.32	2.78788E-94	Proliferating
TOP2A	1.22	7.93203E-95	Proliferating
TUBB	1.21	2.73343E-78	Proliferating
RRM2	1.15	2.18721E-93	Proliferating
CCNB1	1.13	7.85883E-74	Proliferating
HMGN2	1.13	1.07776E-63	Proliferating
CKS2	1.12	1.36871E-85	Proliferating
BIRC5	1.12	2.1405E-113	Proliferating
KIAA0101	1.11	4.72969E-97	Proliferating
KPNA2	1.11	2.57027E-63	Proliferating
CDK1	1.07	6.1104E-99	Proliferating
TK1	1.07	4.9636E-111	Proliferating
HMGB1	1.02	2.41707E-77	Proliferating
ARL6IP1	1.01	7.81013E-33	Proliferating
CENPF	1.00	7.77879E-88	Proliferating
PTTG1	0.98	1.98482E-56	Proliferating
NUSAP1	0.95	1.1848E-103	Proliferating
CENPW	0.88	1.289E-109	Proliferating
MKI67	0.87	2.6852E-102	Proliferating
CDC20	0.85	8.98004E-75	Proliferating
MAD2L1	0.84	5.5293E-104	Proliferating
CCNB2	0.82	9.98237E-88	Proliferating
H2AFV	0.79	5.93649E-72	Proliferating
CDKN3	0.78	5.43421E-91	Proliferating
TYMS	0.77	9.6321E-89	Proliferating
CALM2	0.76	4.68346E-37	Proliferating
KIF20B	0.76	5.03256E-80	Proliferating
RANBP1	0.75	3.51975E-58	Proliferating
PBK	0.74	3.5004E-101	Proliferating
ASPM	0.73	1.77697E-85	Proliferating

PCNA	0.71	5.34923E-25	Proliferating
ZWINT	0.70	1.93143E-87	Proliferating
HNRNPA2B1	0.69	3.92441E-51	Proliferating
CCNA2	0.68	1.08486E-87	Proliferating
CKS1B	0.68	2.52711E-44	Proliferating
PTMA	0.67	1.78757E-75	Proliferating
DUT	0.66	1.23244E-30	Proliferating
PRC1	0.65	5.4032E-94	Proliferating
SMC2	0.64	4.578E-67	Proliferating
AKR1C1	0.63	5.94575E-33	Proliferating
AURKB	0.63	2.54332E-77	Proliferating
TPX2	0.62	2.51659E-85	Proliferating
HSP90AA1	0.61	1.37882E-42	Proliferating
KRT5	0.59	1.00298E-30	Proliferating
NUDT1	0.58	2.22623E-57	Proliferating
DTYMK	0.58	8.82219E-73	Proliferating
PLK1	0.58	7.44939E-58	Proliferating
H2AFX	0.58	1.01156E-89	Proliferating
UBE2T	0.58	3.49937E-90	Proliferating
KRT14	0.58	1	Proliferating
TMEM106C	0.57	4.37693E-42	Proliferating
LMNB1	0.56	4.59228E-90	Proliferating
AKR1C2	0.56	2.44877E-08	Proliferating
BUB3	0.55	3.08376E-38	Proliferating
CCDC34	0.55	8.94707E-54	Proliferating
SRSF3	0.55	3.60336E-39	Proliferating
CENPM	0.55	2.77003E-83	Proliferating
CENPA	0.54	1.71648E-57	Proliferating
CENPE	0.53	1.9246E-64	Proliferating
RAN	0.52	7.82124E-25	Proliferating
SPC25	0.52	2.01643E-79	Proliferating
AKR1B10	0.52	1	Proliferating
STRA13	0.52	2.27428E-34	Proliferating
CENPN	0.52	1.46408E-93	Proliferating
ANP32E	0.51	1.01264E-54	Proliferating
RPA3	0.51	2.23865E-42	Proliferating
HMMR	0.51	1.60349E-68	Proliferating
LSM5	0.51	3.45358E-37	Proliferating
SRSF2	0.50	1.25584E-37	Proliferating
PTGES3	0.50	2.91115E-28	Proliferating
GMNN	0.49	1.27214E-49	Proliferating
SIVA1	0.49	2.87537E-30	Proliferating
TMPO	0.49	1.62369E-54	Proliferating

DDX39A	0.48	5.82698E-49	Proliferating
SGOL1	0.48	7.3066E-81	Proliferating
ASF1B	0.48	1.51618E-77	Proliferating
RNASEH2A	0.46	2.23664E-53	Proliferating
HNRNPA3	0.46	1.91125E-36	Proliferating
PHGDH	0.46	6.63525E-22	Proliferating
FEN1	0.46	8.23995E-56	Proliferating
CDCA3	0.46	4.14886E-64	Proliferating
RRM1	0.46	3.63091E-29	Proliferating
RHEB	0.46	2.52656E-34	Proliferating
LSM4	0.45	1.9905E-49	Proliferating
DEK	0.45	1.44817E-27	Proliferating
YWHAH	0.45	2.48006E-44	Proliferating
SNRPB	0.44	1.28694E-28	Proliferating
MCM7	0.44	5.59056E-21	Proliferating
SNRPD1	0.44	3.62688E-37	Proliferating
SNRPG	0.44	4.93593E-30	Proliferating
CDCA8	0.44	8.48539E-69	Proliferating
BCL7C	0.44	3.52054E-34	Proliferating
SGOL2	0.44	1.31526E-64	Proliferating
RAD21	0.43	4.52237E-30	Proliferating
CDCA4	0.43	2.25052E-35	Proliferating
MZT1	0.43	9.84046E-48	Proliferating
RAD51AP1	0.43	9.22348E-67	Proliferating
DNAJC9	0.42	1.64285E-35	Proliferating
RACGAP1	0.42	3.96012E-65	Proliferating
TUBB4B	0.42	1.55705E-15	Proliferating
SFPQ	0.41	7.28943E-23	Proliferating
MZT2B	0.41	4.78974E-34	Proliferating
CBX3	0.41	1.66332E-26	Proliferating
DHFR	0.41	3.41162E-53	Proliferating
CACYBP	0.40	4.81356E-22	Proliferating
LSM2	0.40	8.95973E-24	Proliferating
HNRNPR	0.40	3.14733E-23	Proliferating
HMGN1	0.39	3.6711E-28	Proliferating
KIFC1	0.39	1.09228E-66	Proliferating
TMEM14A	0.39	1.24163E-27	Proliferating
UBE2S	0.39	2.01181E-46	Proliferating
CKAP2L	0.38	4.18294E-64	Proliferating
ANLN	0.38	4.18789E-67	Proliferating
CKAP2	0.37	3.68784E-29	Proliferating
DEPDC1	0.37	9.31405E-65	Proliferating
CENPU	0.37	5.26041E-44	Proliferating

HNRNPAB	0.37	5.14848E-22	Proliferating
NDC80	0.36	7.00341E-57	Proliferating
NUF2	0.36	3.59691E-68	Proliferating
TAGLN2	0.36	4.3027E-23	Proliferating
CENPH	0.36	3.96245E-30	Proliferating
SLBP	0.36	3.31041E-22	Proliferating
PPIH	0.35	2.58127E-33	Proliferating
ILF2	0.35	2.85998E-31	Proliferating
HADH	0.35	6.22676E-23	Proliferating
CSE1L	0.35	1.32408E-23	Proliferating
CENPK	0.35	3.84603E-55	Proliferating
BOLA3	0.35	1.61676E-26	Proliferating
SKA2	0.35	2.1481E-22	Proliferating
LSMD1	0.34	1.6727E-23	Proliferating
CALM3	0.34	1.26299E-16	Proliferating
POLE3	0.34	8.10415E-27	Proliferating
AURKA	0.34	3.54241E-51	Proliferating
LMNB2	0.34	3.72237E-41	Proliferating
ANP32B	0.34	1.13424E-18	Proliferating
CRNDE	0.34	3.02274E-20	Proliferating
KIF22	0.34	1.35006E-27	Proliferating
GINS2	0.34	2.99044E-19	Proliferating
BANF1	0.34	1.4458E-17	Proliferating
ACTL6A	0.34	3.67081E-27	Proliferating
DBF4	0.34	2.54685E-48	Proliferating
KIF23	0.34	4.78926E-53	Proliferating
TEX30	0.34	6.70087E-43	Proliferating
BSG	0.34	1.82332E-15	Proliferating
CCT5	0.33	6.45661E-17	Proliferating
TACC3	0.33	1.4266E-68	Proliferating
PA2G4	0.33	9.80168E-18	Proliferating
FAM96A	0.33	1.64704E-23	Proliferating
NASP	0.33	1.74864E-08	Proliferating
KNSTRN	0.33	5.22341E-51	Proliferating
NCAPG	0.33	6.36041E-61	Proliferating
ENO1	0.33	5.37822E-16	Proliferating
CDCA5	0.33	1.22621E-61	Proliferating
TCP1	0.33	1.27113E-18	Proliferating
YBX1	0.33	1.29417E-25	Proliferating
CEP55	0.32	1.89115E-55	Proliferating
ERH	0.32	1.34156E-19	Proliferating
ARHGAP11A	0.32	4.50146E-60	Proliferating
PIF1	0.32	8.4387E-32	Proliferating

PSMA4	0.32	1.41904E-27	Proliferating
CSRP2	0.32	2.5727E-16	Proliferating
NUDC	0.32	2.3872E-22	Proliferating
TPM3	0.32	2.67936E-15	Proliferating
SNRPC	0.32	8.59175E-20	Proliferating
KIF2C	0.32	1.10726E-49	Proliferating
XRCC5	0.31	2.49143E-16	Proliferating
DLGAP5	0.31	5.07359E-48	Proliferating
TTK	0.31	1.29564E-55	Proliferating
LSM3	0.31	3.24605E-16	Proliferating
GCSH	0.31	3.32689E-16	Proliferating
ARHGAP11B	0.31	1.52424E-51	Proliferating
USP1	0.31	4.89453E-24	Proliferating
KIF11	0.31	1.03544E-56	Proliferating
VRK1	0.31	6.64127E-43	Proliferating
CYBA	0.31	3.35532E-28	Proliferating
HNRNPF	0.31	5.61549E-14	Proliferating
SRSF7	0.31	5.03983E-14	Proliferating
BCL2L12	0.31	2.12323E-53	Proliferating
ID3	0.31	7.66292E-35	Proliferating
DNMT1	0.30	7.11928E-23	Proliferating
SNRPE	0.30	2.26942E-16	Proliferating
SPC24	0.30	3.24349E-46	Proliferating
SAC3D1	0.30	2.84855E-36	Proliferating
TUBG1	0.30	4.02186E-30	Proliferating
POLD2	0.30	4.83974E-14	Proliferating
HP1BP3	0.30	1.6638E-14	Proliferating
MIS18A	0.30	2.16568E-43	Proliferating
SRP9	0.30	5.20423E-17	Proliferating
GGH	0.30	3.80001E-36	Proliferating
PAFAH1B3	0.29	5.49494E-17	Proliferating
NUCKS1	0.29	1.01878E-10	Proliferating
CKLF	0.29	6.4576E-35	Proliferating
ORC6	0.29	4.70333E-53	Proliferating
TFDP1	0.29	1.27818E-17	Proliferating
CDT1	0.29	1.54137E-23	Proliferating
PSRC1	0.29	4.62564E-40	Proliferating
FAM111B	0.29	5.53959E-34	Proliferating
RUVBL2	0.29	2.43361E-14	Proliferating
UQCRC1	0.28	1.38227E-14	Proliferating
EXOSC8	0.28	2.37744E-26	Proliferating
GTSE1	0.28	8.34438E-51	Proliferating
LYAR	0.28	1.67912E-27	Proliferating

TROAP	0.28	8.64907E-47	Proliferating
NME1	0.28	9.34276E-15	Proliferating
PSMA3	0.28	3.24653E-17	Proliferating
ECT2	0.28	2.17159E-54	Proliferating
HNRNPD	0.28	8.46034E-18	Proliferating
SAE1	0.28	7.90512E-25	Proliferating
HJURP	0.28	7.02847E-53	Proliferating
SRM	0.28	2.06357E-16	Proliferating
WDR34	0.28	9.94818E-18	Proliferating
PXMP2	0.28	2.27492E-21	Proliferating
GSTO1	0.28	1.6431E-12	Proliferating
HAT1	0.28	7.03242E-18	Proliferating
TRA2B	0.28	2.58729E-12	Proliferating
SNRPA1	0.27	2.68681E-18	Proliferating
MND1	0.27	9.60542E-51	Proliferating
CHCHD2	0.27	3.85876E-09	Proliferating
H3F3A	0.27	5.07465E-24	Proliferating
ISOC2	0.27	1.14041E-13	Proliferating
RPL22L1	0.27	1.41366E-12	Proliferating
U2AF1	0.27	1.41952E-14	Proliferating
MAGOH	0.27	7.93847E-14	Proliferating
TUFM	0.27	1.61166E-12	Proliferating
OIP5	0.27	3.26453E-56	Proliferating
GPAA1	0.27	6.45895E-13	Proliferating
MXD3	0.27	2.56345E-42	Proliferating
NCAPD2	0.27	3.52008E-46	Proliferating
KPNB1	0.27	1.75035E-16	Proliferating
MELK	0.27	4.15472E-56	Proliferating
HNRNPM	0.27	4.04699E-12	Proliferating
HSPA8	0.26	3.17449E-13	Proliferating
CNIH4	0.26	1.5272E-14	Proliferating
NCL	0.26	3.53418E-05	Proliferating
RFC2	0.26	4.06538E-21	Proliferating
CDC25B	0.26	1.67578E-44	Proliferating
PSMA2.1	0.26	2.30249E-15	Proliferating
MRPL11	0.26	1.50117E-13	Proliferating
PSMG1	0.26	1.51682E-16	Proliferating
JTB	0.26	6.99943E-11	Proliferating
ANXA5	0.26	4.51867E-20	Proliferating
ZDHHC12	0.26	1.82107E-18	Proliferating
NEK2	0.25	4.22482E-49	Proliferating
MTHFD2	0.25	6.15613E-31	Proliferating
ZWILCH	0.25	1.92442E-35	Proliferating

COMMD4	0.25	5.1198E-17	Proliferating
HSPE1	0.25	6.80518E-20	Proliferating
NBL1	0.25	3.63088E-12	Proliferating
SMC3	0.25	3.11469E-15	Proliferating
LSM6	0.25	1.4999E-16	Proliferating
DSC2	1.06	1.05178E-77	Trans1
DSP	0.79	1.53007E-50	Trans1
DSG3	0.70	6.99232E-40	Trans1
CDH1	0.47	1.17001E-17	Trans1
PKP1	0.47	5.94721E-44	Trans1
TRIM29	0.42	1.26406E-12	Trans1
MAST4	0.42	1.57409E-13	Trans1
ELL2	0.39	5.64738E-24	Trans1
RP11-408H20.1	0.37	3.18558E-21	Trans1
DHCR24	0.36	2.95446E-06	Trans1
GJA1	0.36	7.92891E-20	Trans1
PPM1K	0.36	4.09013E-14	Trans1
MAF	0.35	6.12177E-07	Trans1
CTTNBP2	0.35	4.71444E-16	Trans1
DSG1	0.34	2.13705E-17	Trans1
RAPGEFL1	0.34	6.07697E-08	Trans1
KANK1	0.34	2.94728E-16	Trans1
ZNF770	0.34	6.81825E-10	Trans1
^{HIGH}F1A	0.31	1.45412E-08	Trans1
MTMR10	0.31	6.98599E-14	Trans1
ADH7	0.30	7.16747E-10	Trans1
PLEKHN1	0.30	2.98314E-17	Trans1
EIF4G1	0.30	5.98345E-05	Trans1
MT-CO2	0.30	1	Trans1
JAG1	0.29	8.14319E-07	Trans1
NBEAL2	0.29	4.7587E-08	Trans1
CLCA2	0.29	5.4925E-22	Trans1
^{HIGH}PK2	0.28	5.12921E-05	Trans1
NOTCH3	0.28	0.000291883	Trans1
PVRL1	0.28	2.02868E-07	Trans1
DLG1	0.28	7.40998E-08	Trans1
SLC26A2	0.27	2.43219E-07	Trans1
ATP2A2	0.27	0.00018642	Trans1
SAMD5	0.27	3.41606E-10	Trans1
LINC00657	0.27	1.02031E-06	Trans1
PROM2	0.26	6.62674E-06	Trans1
DUOX1	0.26	2.37656E-18	Trans1

ZHX1	0.26	1.9529E-06	Trans1
FGFR3	0.25	0.003061579	Trans1
WNT5A	0.25	2.42432E-13	Trans1
MYH14	0.25	1.60835E-20	Trans1
SERPINB3	0.57	1.04147E-07	Trans2
ACTB	0.49	1.3611E-25	Trans2
CSTA	0.47	1.77326E-30	Trans2
HSPB1	0.46	4.8733E-62	Trans2
MSMO1	0.41	1	Trans2
PRDX5	0.39	1	Trans2
SH3BGRL3	0.38	1	Trans2
CST3	0.38	1	Trans2
SERPINB4	0.35	0.111596147	Trans2
CRABP2	0.35	0.004516629	Trans2
CTNNBIP1	0.34	1	Trans2
NUCB2	0.33	1	Trans2
RAB25	0.33	1	Trans2
S100A16	0.32	9.60046E-17	Trans2
S100A11	0.32	8.58917E-38	Trans2
ORMDL2	0.31	1	Trans2
ARPC3	0.31	1	Trans2
GNAI3	0.30	1	Trans2
SLPI	0.30	1	Trans2
TUBA4A	0.29	0.031452933	Trans2
LYPD3	0.28	1	Trans2
ANXA2	0.28	2.2072E-07	Trans2
FAM162A	0.27	1	Trans2
PI3	0.27	1	Trans2
AP2S1	0.27	1	Trans2
CXCL17	0.27	1	Trans2
TMED2	0.27	1	Trans2
S100A2	0.26	1.88365E-12	Trans2
FAM3B	0.25	1	Trans2
ENSA	0.25	1	Trans2
SULT2B1	0.25	1	Trans2
PIM1	1.14	3.71391E-86	Differentiated^{low}
AIM1	1.04	4.27103E-60	Differentiated^{low}
FAM3D	0.92	1.15966E-93	Differentiated^{low}
CLCA4	0.91	2.35425E-74	Differentiated^{low}
DIO2	0.91	8.52733E-65	Differentiated^{low}
ATP1B1	0.79	6.59538E-70	Differentiated^{low}

RP11-7K24.3	0.77	2.51701E-65	Differentiated^{low}
WDR26	0.71	8.50069E-85	Differentiated^{low}
WNK1	0.70	2.9824E-31	Differentiated^{low}
RP11-351J23.1	0.69	4.96673E-32	Differentiated^{low}
MLLT4	0.68	4.11017E-86	Differentiated^{low}
CTSV	0.65	3.90909E-31	Differentiated^{low}
PADI1	0.65	1.28083E-31	Differentiated^{low}
TGM3	0.64	3.0029E-115	Differentiated^{low}
EVPL	0.63	2.20309E-28	Differentiated^{low}
TACSTD2	0.63	5.77961E-57	Differentiated^{low}
LL22NC03-2H8.5	0.63	1.68602E-11	Differentiated^{low}
CRNN	0.61	4.9504E-123	Differentiated^{low}
GRHL1	0.60	1.3496E-33	Differentiated^{low}
TJP1	0.60	1.32298E-48	Differentiated^{low}
DOCK9	0.59	9.37259E-59	Differentiated^{low}
TMPRSS11B	0.59	6.60168E-61	Differentiated^{low}
NPEPPS	0.58	7.91988E-38	Differentiated^{low}
EPS8L2	0.57	2.295E-63	Differentiated^{low}
GRB7	0.57	6.514E-16	Differentiated^{low}
H1F0	0.56	2.01831E-58	Differentiated^{low}
MPZL3	0.55	4.07675E-54	Differentiated^{low}
CLDN10	0.55	7.13639E-18	Differentiated^{low}
ATG9B	0.55	1.80075E-15	Differentiated^{low}
CD46	0.55	1.17146E-22	Differentiated^{low}
CCDC64B	0.54	1.00987E-45	Differentiated^{low}
HECTD1	0.54	2.62926E-43	Differentiated^{low}
KLK13	0.54	8.8706E-52	Differentiated^{low}
AL627309.1	0.53	2.37389E-13	Differentiated^{low}
UACA	0.53	1.11175E-15	Differentiated^{low}
CEACAM6	0.52	2.30033E-33	Differentiated^{low}
PPL	0.52	1.34749E-43	Differentiated^{low}
ST3GAL4	0.52	2.01672E-44	Differentiated^{low}
SPINK5	0.51	7.7957E-92	Differentiated^{low}
ARRDC3	0.51	5.13454E-37	Differentiated^{low}
MUC1	0.51	0.078125016	Differentiated^{low}
ERBB3	0.50	1.03113E-23	Differentiated^{low}

PLEKHM1	0.50	3.99258E-20	Differentiated^{low}
EPS8L1	0.50	1.5691E-30	Differentiated^{low}
GPX3	0.49	2.65715E-55	Differentiated^{low}
DUSP1	0.48	2.53063E-28	Differentiated^{low}
VSIG10L	0.47	8.49657E-34	Differentiated^{low}
ARHGAP27	0.46	1.02355E-19	Differentiated^{low}
ABLIM3	0.46	3.04214E-15	Differentiated^{low}
RBM47	0.46	2.03497E-32	Differentiated^{low}
NAPRT1	0.46	1.16303E-54	Differentiated^{low}
NRBP1	0.45	6.53605E-09	Differentiated^{low}
EMP1	0.45	1.0178E-114	Differentiated^{low}
ITPRIPL2	0.45	1.50581E-17	Differentiated^{low}
VPS4B	0.45	1.37607E-46	Differentiated^{low}
SFT2D2	0.45	1.10461E-24	Differentiated^{low}
CRYAB	0.45	3.95604E-27	Differentiated^{low}
GMDS	0.45	5.69423E-33	Differentiated^{low}
LPIN1	0.44	0.000227592	Differentiated^{low}
ABLIM1	0.44	5.56916E-43	Differentiated^{low}
FAM129B	0.44	7.64142E-32	Differentiated^{low}
ZNF117	0.44	6.57707E-30	Differentiated^{low}
CAPN14	0.44	3.23936E-20	Differentiated^{low}
SLK	0.43	2.79346E-41	Differentiated^{low}
NDUFA4L2	0.43	1.60071E-40	Differentiated^{low}
SHROOM3	0.42	2.61493E-28	Differentiated^{low}
SLC16A3	0.42	3.89557E-41	Differentiated^{low}
CLIP1	0.42	1.13881E-25	Differentiated^{low}
NABP1	0.41	5.06094E-16	Differentiated^{low}
CEACAM1	0.41	1.64916E-31	Differentiated^{low}
JMJD1C	0.41	6.11933E-23	Differentiated^{low}
ATP6V1C2	0.41	4.16364E-17	Differentiated^{low}
RANBP9	0.41	4.23437E-31	Differentiated^{low}
RP11-575H3.1	0.40	1.47681E-12	Differentiated^{low}
MALL	0.40	1.1152E-60	Differentiated^{low}
FAM83A	0.39	3.94753E-39	Differentiated^{low}
ARHGAP5	0.39	1.6199E-11	Differentiated^{low}
LY6G6C	0.39	8.47691E-18	Differentiated^{low}
AFF4	0.39	1.36615E-18	Differentiated^{low}
RNF222	0.39	4.13585E-38	Differentiated^{low}

LRP10	0.39	2.73329E-34	Differentiated^{low}
DTX2	0.39	1.76824E-12	Differentiated^{low}
KLK10	0.39	2.26262E-15	Differentiated^{low}
NCOA2	0.38	5.58231E-16	Differentiated^{low}
VAT1	0.38	3.16942E-20	Differentiated^{low}
MARCKS	0.38	3.51552E-18	Differentiated^{low}
MROH6	0.38	3.41472E-31	Differentiated^{low}
CAST	0.38	2.91517E-44	Differentiated^{low}
C2orf54	0.38	2.56431E-26	Differentiated^{low}
ULK3	0.38	1.24452E-20	Differentiated^{low}
DENND2C	0.37	1.32618E-24	Differentiated^{low}
CPEB4	0.37	3.01937E-21	Differentiated^{low}
ZDHHC13	0.37	2.10048E-06	Differentiated^{low}
PRKCI	0.37	4.20553E-08	Differentiated^{low}
PKN2	0.37	4.3275E-05	Differentiated^{low}
PADI3	0.36	5.4888E-07	Differentiated^{low}
PPP4R1	0.36	2.49557E-24	Differentiated^{low}
ANXA9	0.36	8.99583E-15	Differentiated^{low}
CSTB	0.36	1.14729E-84	Differentiated^{low}
MKNK2	0.36	1	Differentiated^{low}
NUP214	0.36	0.00400625	Differentiated^{low}
SLURP1	0.35	3.08587E-05	Differentiated^{low}
MUC4	0.35	0.14969306	Differentiated^{low}
PRDM1	0.35	1.30239E-11	Differentiated^{low}
LMO7	0.35	1.10079E-39	Differentiated^{low}
ANGPTL4	0.35	4.3316E-12	Differentiated^{low}
CXXC5	0.35	1.02069E-19	Differentiated^{low}
SORBS2	0.35	8.66183E-12	Differentiated^{low}
PDCD6	0.35	2.00005E-13	Differentiated^{low}
KIF21A	0.35	1	Differentiated^{low}
CLDND1	0.34	2.27093E-07	Differentiated^{low}
GPT2	0.34	8.11925E-15	Differentiated^{low}
RAB11A	0.34	3.14782E-23	Differentiated^{low}
FCHO2	0.34	4.19246E-11	Differentiated^{low}
EIF4G2	0.34	6.51801E-22	Differentiated^{low}
KIAA0247	0.34	1.1621E-12	Differentiated^{low}
TSC22D4	0.33	2.81845E-08	Differentiated^{low}
BNIPL	0.33	1.26293E-23	Differentiated^{low}

ARHGEF10L	0.33	5.59722E-13	Differentiated^{low}
ARHGAP40	0.33	8.36316E-15	Differentiated^{low}
TMEM45B	0.33	1.37623E-21	Differentiated^{low}
RAB5B	0.33	6.69505E-08	Differentiated^{low}
SERPINB6	0.33	1.16779E-20	Differentiated^{low}
TPRG1	0.33	3.88243E-21	Differentiated^{low}
KIF1C	0.33	1.08286E-10	Differentiated^{low}
ASPG	0.33	4.11526E-21	Differentiated^{low}
C1orf116	0.33	1.77821E-25	Differentiated^{low}
RERE	0.32	9.15348E-12	Differentiated^{low}
INPPL1	0.32	1.43885E-06	Differentiated^{low}
PMM1	0.32	1.16197E-09	Differentiated^{low}
TJP2	0.32	3.93541E-09	Differentiated^{low}
ANXA1	0.32	1.64292E-21	Differentiated^{low}
NCCRP1	0.32	3.59165E-47	Differentiated^{low}
KAZN	0.32	5.9939E-10	Differentiated^{low}
CCNG2	0.32	9.25839E-30	Differentiated^{low}
SAMD4B	0.32	5.92667E-08	Differentiated^{low}
DAPP1	0.31	1.79959E-11	Differentiated^{low}
TOM1L2	0.31	0.000256163	Differentiated^{low}
KIAA0232	0.31	3.05554E-09	Differentiated^{low}
MCL1	0.31	5.42829E-15	Differentiated^{low}
PLEKHA6	0.31	7.43158E-26	Differentiated^{low}
CNOT1	0.31	6.61897E-10	Differentiated^{low}
SCNN1B	0.31	4.59246E-14	Differentiated^{low}
EPB41L3	0.31	7.61157E-34	Differentiated^{low}
TRNP1	0.31	2.16512E-14	Differentiated^{low}
SH3PXD2A	0.30	3.4902E-05	Differentiated^{low}
ARHGAP32	0.30	0.318569094	Differentiated^{low}
NCKAP1	0.30	5.65326E-07	Differentiated^{low}
NUMA1	0.30	1	Differentiated^{low}
CYTH1	0.30	7.55777E-08	Differentiated^{low}
ABHD17C	0.30	5.22026E-13	Differentiated^{low}
AMFR	0.30	2.65433E-10	Differentiated^{low}
EPHA2	0.30	0.000604687	Differentiated^{low}
CEACAM5	0.29	1.35401E-17	Differentiated^{low}
ADD3	0.29	1	Differentiated^{low}
CAMSAP3	0.29	2.51739E-13	Differentiated^{low}

CTNNA1	0.29	1.06202E-09	Differentiated^{low}
FNDC4	0.29	4.48239E-31	Differentiated^{low}
CLCN3	0.29	8.67392E-05	Differentiated^{low}
CANT1	0.29	4.60768E-17	Differentiated^{low}
SPINK7	0.29	0.068676449	Differentiated^{low}
CALM1	0.29	2.65019E-19	Differentiated^{low}
AGFG2	0.28	6.37418E-20	Differentiated^{low}
TUBB6	0.28	1	Differentiated^{low}
ATP13A4	0.28	3.97232E-17	Differentiated^{low}
HBP1	0.28	1.33203E-18	Differentiated^{low}
GALE	0.28	0.002099859	Differentiated^{low}
CPNE3	0.28	1.91481E-06	Differentiated^{low}
PLXNB2	0.28	1	Differentiated^{low}
SH3GL1	0.28	2.95449E-16	Differentiated^{low}
KCTD11	0.28	2.36223E-11	Differentiated^{low}
CCNYL1	0.28	5.94757E-24	Differentiated^{low}
UGCG	0.28	1.94603E-10	Differentiated^{low}
UPK1A	0.28	2.26985E-21	Differentiated^{low}
MYO6	0.27	0.042130871	Differentiated^{low}
GLTPD1	0.27	0.019923133	Differentiated^{low}
YPEL3	0.27	0.009928825	Differentiated^{low}
KAT2B	0.27	1.46067E-28	Differentiated^{low}
SLC11A2	0.27	1.65931E-13	Differentiated^{low}
ACADM	0.27	1.3789E-08	Differentiated^{low}
SNX21	0.27	8.76865E-08	Differentiated^{low}
DGKA	0.27	1	Differentiated^{low}
SGPL1	0.27	0.66637086	Differentiated^{low}
AIM1L	0.27	1.08546E-06	Differentiated^{low}
CLIC3	0.26	2.54119E-13	Differentiated^{low}
IST1	0.26	1	Differentiated^{low}
SLC8A1-AS1	0.26	6.54852E-11	Differentiated^{low}
HCAR2	0.26	1.03068E-13	Differentiated^{low}
DGAT2	0.26	1.96477E-10	Differentiated^{low}
INO80C	0.26	4.67924E-06	Differentiated^{low}
FRMD4B	0.26	0.227635752	Differentiated^{low}
FBXO34	0.26	4.6945E-10	Differentiated^{low}
KLHL2	0.26	8.71428E-08	Differentiated^{low}
KATNBL1	0.26	3.75333E-08	Differentiated^{low}

AIFM2	0.26	0.001273961	Differentiated^{low}
C9orf169	0.25	2.77737E-19	Differentiated^{low}
ITSN2	0.25	1.60796E-11	Differentiated^{low}
MPRIP	0.25	8.16542E-05	Differentiated^{low}
ESPL1	0.25	0.009032713	Differentiated^{low}
NPAS2	0.25	6.76628E-12	Differentiated^{low}
B3GNT3	0.25	1.6332E-13	Differentiated^{high}
MT1G	3.18	2.53104E-28	Differentiated^{high}
MT1E	2.16	1.47469E-10	Differentiated^{high}
MT1H	2.02	5.8043E-11	Differentiated^{high}
RNASE7	1.84	6.3068E-103	Differentiated^{high}
MT2A	1.81	4.23694E-05	Differentiated^{high}
KRT16	1.47	1.72756E-66	Differentiated^{high}
^{HIGH}LPDA	1.46	1.9407E-99	Differentiated^{high}
SPRR2D	1.45	3.2634E-129	Differentiated^{high}
DKK1	1.18	4.105E-15	Differentiated^{high}
KRTAP3-2	1.16	0.042555029	Differentiated^{high}
ACTG1	1.06	1.51505E-87	Differentiated^{high}
MT1M	1.01	0.289291817	Differentiated^{high}
KPRP	0.99	2.4605E-38	Differentiated^{high}
NFKBIA	0.96	6.89534E-32	Differentiated^{high}
RND3	0.92	1.9069E-123	Differentiated^{high}
BPGM	0.91	4.59498E-10	Differentiated^{high}
SPRR2E	0.90	1.48452E-46	Differentiated^{high}
DUSP5	0.90	2.0004E-135	Differentiated^{high}
GLUL	0.89	6.07694E-51	Differentiated^{high}
FTH1	0.86	1.0511E-136	Differentiated^{high}
HOTAIRM1	0.86	1.97495E-22	Differentiated^{high}
GADD45B	0.85	1.33179E-52	Differentiated^{high}
MT1F	0.84	1	Differentiated^{high}
NR1D1	0.81	1.19702E-32	Differentiated^{high}
SPRR2A	0.81	1.9337E-136	Differentiated^{high}
ANKRD37	0.81	2.62073E-18	Differentiated^{high}
CHAC1	0.75	7.30852E-66	Differentiated^{high}
C15orf48	0.74	1.9277E-101	Differentiated^{high}
CITED2	0.68	5.16325E-53	Differentiated^{high}
IL23A	0.66	1.66613E-13	Differentiated^{high}
PMAIP1	0.65	1.54453E-34	Differentiated^{high}

SPRR2F	0.64	1.16406E-09	Differentiated^{high}
ADM	0.64	1.256E-36	Differentiated^{high}
S100P	0.63	5.6071E-56	Differentiated^{high}
GABARAPL2	0.63	2.30301E-75	Differentiated^{high}
FLG	0.61	6.60747E-27	Differentiated^{high}
UBC	0.59	4.37389E-80	Differentiated^{high}
PCBP1	0.58	7.18424E-66	Differentiated^{high}
PPDPF	0.57	2.8362E-127	Differentiated^{high}
FAM25A	0.56	8.49985E-94	Differentiated^{high}
SQSTM1	0.56	2.70482E-76	Differentiated^{high}
IER3	0.55	9.03366E-21	Differentiated^{high}
GADD45A	0.55	0.011069541	Differentiated^{high}
SPRR2B	0.54	6.14324E-11	Differentiated^{high}
KRT17	0.53	1	Differentiated^{high}
PHLDA1	0.52	6.50015E-54	Differentiated^{high}
UPP1	0.51	1.90523E-25	Differentiated^{high}
CRCT1	0.51	3.50242E-91	Differentiated^{high}
ADIRF	0.51	1.33511E-66	Differentiated^{high}
MT1X	0.49	1	Differentiated^{high}
MAFF	0.47	3.87193E-54	Differentiated^{high}
PPIF	0.47	1	Differentiated^{high}
FAM45A	0.46	2.00669E-41	Differentiated^{high}
MAP1LC3B	0.46	1.12909E-68	Differentiated^{high}
ALDH1A3	0.46	4.32153E-45	Differentiated^{high}
ZFP36	0.44	3.58478E-20	Differentiated^{high}
RFK	0.44	1.73549E-56	Differentiated^{high}
RASGEF1B	0.43	6.92539E-49	Differentiated^{high}
MGAT1	0.42	2.66911E-47	Differentiated^{high}
JUNB	0.41	1.06847E-15	Differentiated^{high}
LGALS3	0.40	1.9962E-21	Differentiated^{high}
TUFT1	0.40	1.23162E-48	Differentiated^{high}
YOD1	0.40	5.34224E-38	Differentiated^{high}
VMP1	0.40	3.52077E-28	Differentiated^{high}
SPRR1B	0.38	1.73537E-30	Differentiated^{high}
CXCL2	0.38	0.610362532	Differentiated^{high}
DEFB4A	0.37	1.54524E-05	Differentiated^{high}
LCN2	0.36	2.62347E-27	Differentiated^{high}
NDFIP2	0.36	1.6065E-42	Differentiated^{high}

CXCL3	0.35	1	Differentiated^{high}
TM9SF2	0.34	1	Differentiated^{high}
ISG15	0.34	1	Differentiated^{high}
UBE2V1	0.33	2.13951E-34	Differentiated^{high}
DHRS9	0.33	1.30631E-42	Differentiated^{high}
ATP8B1	0.33	2.46045E-07	Differentiated^{high}
PRSS22	0.33	4.23968E-16	Differentiated^{high}
TPM4	0.32	2.50065E-29	Differentiated^{high}
MAPK8	0.32	2.23438E-33	Differentiated^{high}
RAP2B	0.32	1.98361E-30	Differentiated^{high}
DUOXA2	0.32	3.81058E-21	Differentiated^{high}
EIF1	0.32	1.09893E-21	Differentiated^{high}
TTC9	0.32	1.05084E-30	Differentiated^{high}
IL1A	0.32	9.06695E-17	Differentiated^{high}
HSPB8	0.31	1.15077E-24	Differentiated^{high}
UBE2D3	0.31	4.24245E-10	Differentiated^{high}
HLA-E	0.31	1.94936E-07	Differentiated^{high}
RMND5A	0.31	4.9753E-35	Differentiated^{high}
ZNF812	0.30	1.0142E-12	Differentiated^{high}
HLCS	0.30	2.748E-21	Differentiated^{high}
RP11-532F12.5	0.30	1.45457E-32	Differentiated^{high}
TMEM159	0.29	1.6899E-10	Differentiated^{high}
FEM1C	0.27	4.64417E-06	Differentiated^{high}
DUSP14	0.27	3.99427E-22	Differentiated^{high}
GRPEL2	0.27	6.48333E-09	Differentiated^{high}
RP11-390E23.6	0.27	3.86342E-22	Differentiated^{high}
AVPI1	0.27	2.0135E-11	Differentiated^{high}
NFKBIZ	0.27	2.44796E-38	Differentiated^{high}
RNF145	0.27	4.95091E-23	Differentiated^{high}
POLR1D	0.27	2.48118E-14	Differentiated^{high}
SERTAD2	0.26	1.20923E-34	Differentiated^{high}
ARL4C	0.26	3.68277E-18	Differentiated^{high}
