# Supplementary material for: Single-cell RNA-Seq of human esophageal epithelium in homeostasis and allergic inflammation
Source: JCI Insight. 2022 Jun 8;7(11):e159093. doi: 10.1172/jci.insight.159093 (PMC9208762; doi:10.1172/jci.insight.159093)
Supplement: Supplemental table 3 [file jciinsight-7-159093-s301.pdf]

Supplemental Table 3. Markers of the human esophageal basal epithelial layers

**basal layer markers ***	**RPKM basal**	**RPKM suprabasal**	**ratio basal/suprabasal**
IGFBP3	6.21	0.01	620.58
VIT	9.13	0.18	51.93
CH25H	7.29	0.16	44.44
ABI3BP	5.15	0.15	35.25
DKK3	7.03	0.20	34.40
COL17A1	81.54	2.39	34.10
BGN	9.80	0.33	29.26
CDH13	9.63	0.34	28.38
TNS3	6.86	0.34	20.09
CAV1	11.40	0.61	18.80
EFEMP1	13.35	0.75	17.70
CYR61	9.36	0.58	16.08
MXRA5	27.39	1.72	15.96
TPM2	6.63	0.42	15.64
HOPX	5.42	0.36	14.91
BASP1	8.45	0.58	14.58
DST	125.92	8.68	14.51
CYP2S1	12.03	0.86	13.94
ASS1	18.44	1.33	13.82
CCDC3	17.63	1.30	13.53
TNS1	8.47	0.63	13.42
ADH1C	6.04	0.46	13.03
DLK2	5.76	0.45	12.91
SLC7A5	21.60	1.89	11.43
FHL2	5.84	0.51	11.37
NTRK2	5.68	0.51	11.20
LAMB3	18.93	1.76	10.76
ABCC3	8.43	0.80	10.55
MOXD1	17.34	1.89	9.18
SLC40A1	6.58	0.76	8.66
CFB	5.33	0.64	8.31
BOC	7.38	0.90	8.16
GPNMB	103.75	13.00	7.98
FOXQ1	7.57	0.95	7.97
IGFBP5	20.10	2.72	7.40
ZFP36L2	8.98	1.23	7.29
ABCA1	9.40	1.29	7.28
TP53I11	7.24	0.99	7.28

WNT4	15.05	2.08	7.25
IFITM1	6.80	0.98	6.94
TIMP1	7.94	1.18	6.72
NPR3	17.53	2.63	6.67
SLC1A3	20.29	3.12	6.50
PCDH7	6.88	1.12	6.16
TFAP2B	5.34	0.87	6.11
ANKH	6.29	1.04	6.05
LEPREL1	9.42	1.56	6.04
CXCL14	52.82	8.95	5.90
GABRE	5.09	0.88	5.76
KRT14	14.60	2.56	5.71
HIST1H3I	7.45	1.34	5.58
TNS4	33.36	5.99	5.56
SLC1A4	11.75	2.12	5.53
ITGB4	11.45	2.16	5.30
CYBRD1	7.49	1.42	5.27
AP2B1	11.20	2.19	5.11
NPNT	6.26	1.23	5.07
ANTXR1	7.40	1.47	5.04
ZMYM6NB	15.10	3.02	5.01
BEX2	8.03	1.65	4.88
GLUL	12.82	2.63	4.87
SOX6	5.05	1.05	4.83
FAT1	7.85	1.64	4.78
PINLYP	6.79	1.48	4.59
MEGF6	8.09	1.78	4.53
SLC3A2	10.22	2.28	4.47
PTRF	12.02	2.86	4.21
FLNA	6.82	1.65	4.13
CDHR1	9.14	2.28	4.01
IL18	29.31	7.50	3.91
KRT19	53.41	13.73	3.89
TXNIP	30.89	7.95	3.88
TFCP2L1	8.98	2.31	3.88
SESN3	14.93	3.86	3.86
PTPN14	6.67	1.74	3.83
BTG2	10.77	2.82	3.81
EHD3	7.58	2.04	3.72
KRT15	702.82	189.20	3.71
TANC2	6.26	1.71	3.66
PTPRS	8.34	2.29	3.64
SH3PXD2A	9.20	2.54	3.62

PRNP	11.98	3.33	3.60
LAPTM4B	13.82	3.90	3.55
C21orf91	6.81	1.92	3.54
MEF2A	5.05	1.45	3.47
ETS2	7.87	2.33	3.38
CTDSPL	6.91	2.04	3.38
NRM	7.08	2.10	3.37
CLSTN1	9.04	2.68	3.37
SLC2A1	29.74	9.15	3.25
TPBG	8.73	2.70	3.23
PTPRZ1	10.43	3.24	3.22
CRIM1	6.28	1.95	3.22
PRKAB2	5.00	1.55	3.22
ZNF91	5.23	1.65	3.16
GLTSCR2	13.93	4.44	3.14
BEX4	5.57	1.79	3.12
AJUBA	5.50	1.79	3.08
LAMB2	5.30	1.73	3.06
SNAI2	17.40	5.72	3.04
ARL4C	5.50	1.83	3.01
RBM4B	5.24	1.74	3.01
DHRS3	14.83	4.95	3.00
LGALS8	8.11	2.72	2.99
LAMC1	5.11	1.71	2.99
CCND2	9.56	3.20	2.98
LAMA3	7.64	2.60	2.93
RBBP8	11.22	3.88	2.89
ZNF395	5.46	1.90	2.88
APCDD1	20.11	7.04	2.86
SLC43A3	12.24	4.33	2.83
NAP1L1	13.98	4.94	2.83
CROT	5.08	1.82	2.80
AHCYL2	8.71	3.13	2.79
C2CD2	6.39	2.30	2.79
GSR	6.28	2.26	2.78
IFITM3	33.82	12.20	2.77
LIMA1	11.12	4.05	2.75
HLF	11.61	4.24	2.74
SELENBP1	6.39	2.36	2.71
SP100	5.75	2.12	2.71
EGR1	45.41	16.78	2.71
CRTAP	7.64	2.83	2.70
MXRA7	5.83	2.17	2.68

ITGB1	8.12	3.04	2.67
STOM	9.69	3.64	2.66
FTH1	62.96	23.67	2.66
FBXW7	5.14	1.95	2.64
CD74	7.23	2.75	2.63
TLR3	5.09	1.94	2.62
FGD6	6.09	2.33	2.62
CTDSP2	7.93	3.07	2.58
HAUS4	5.54	2.18	2.54
HSPA2	8.23	3.27	2.52
NF1	5.14	2.05	2.51
ST6GALNAC2	11.95	4.80	2.49
CDCA7	6.30	2.54	2.48
LDB1	9.70	3.93	2.47
DSG2	6.04	2.48	2.44
THSD4	21.51	8.87	2.43
DTX3L	5.01	2.09	2.40
STON2	7.57	3.18	2.38
PBX2	8.95	3.78	2.36
SLC7A1	49.11	20.98	2.34
PHF3	9.64	4.21	2.29
CALCOCO1	13.01	5.70	2.28
KDM6A	5.34	2.34	2.28
PARP14	7.41	3.26	2.27
AMOTL1	7.08	3.15	2.25
GSTM2	10.07	4.58	2.20
CHD3	8.38	3.83	2.19

* the green font denotes esophagus-specific genes
