# Supplementary material for: Single-cell RNA-Seq of human esophageal epithelium in homeostasis and allergic inflammation
Source: JCI Insight. 2022 Jun 8;7(11):e159093. doi: 10.1172/jci.insight.159093 (PMC9208762; doi:10.1172/jci.insight.159093)
Supplement: Supplemental table 4 [file jciinsight-7-159093-s302.pdf]

Supplemental Table 4. Markers of the human esophageal suprabasal epithelial layers

suprabasal layer markers*	RPKM basal	RPKM suprabasal	ratio basal/suprabasal
SERPINB3	2.18	36.71	0.06
KRT4	34.91	586.30	0.06
ALDH1A1	0.45	6.69	0.07
TGM3	1.11	13.07	0.08
GPX3	0.66	7.34	0.09
CXCL17	3.04	32.80	0.09
A2ML1	3.58	36.68	0.10
RHCG	3.52	34.55	0.10
ALDH3B2	0.96	9.13	0.11
SPRR1B	2.80	26.53	0.11
KRT6C	3.02	27.51	0.11
EPHX3	1.16	10.16	0.11
KRT6B	1.73	15.13	0.11
C2orf54	0.63	5.36	0.12
S100A8	38.84	330.82	0.12
CLCA4	0.62	5.21	0.12
SPINK5	7.43	61.98	0.12
CYP2C18	0.61	5.05	0.12
SPRR3	29.11	238.55	0.12
MUC15	0.79	5.93	0.13
KRT6A	20.25	149.68	0.14
S100A9	5.07	36.57	0.14
DSG1	1.53	11.01	0.14
KRT1	0.80	5.53	0.14
SCEL	1.04	7.20	0.15
C10orf99	1.95	12.43	0.16
CSTA	76.50	447.69	0.17
SPARCL1	1.48	8.60	0.17
SAMHD1	2.09	11.96	0.17
CSTB	32.53	185.73	0.18
SERPINB13	2.34	12.72	0.18
ZNF750	3.00	16.21	0.19
KRT13	307.29	1575.82	0.20
RHOV	1.26	6.43	0.20
SLC39A2	1.10	5.40	0.20
KLK13	8.57	41.85	0.20
SCNN1A	1.73	7.95	0.22
CRABP2	12.92	57.13	0.23

CTTNBP2	1.18	5.22	0.23
GRHL3	3.73	16.13	0.23
SLPI	1.69	7.14	0.24
S100A11	254.83	998.88	0.26
DSC2	12.12	47.40	0.26
SCIN	2.23	8.71	0.26
BARX2	4.84	18.59	0.26
LPAR5	2.43	9.34	0.26
PTGS1	1.62	6.12	0.26
VPS25	2.12	7.90	0.27
BBOX1	1.74	6.35	0.27
PVRL4	1.72	6.16	0.28
KLK11	3.16	11.16	0.28
LYPD3	10.39	36.64	0.28
SERPINB1	3.02	10.48	0.29
FGFBP1	2.45	8.06	0.30
ANXA1	90.94	298.35	0.30
NUCB2	1.80	5.89	0.31
AQP3	62.14	202.78	0.31
FABP5	42.59	138.72	0.31
CALML3	4.28	13.89	0.31
NAGK	2.16	6.95	0.31
DYNLT3	1.76	5.63	0.31
CNN3	1.82	5.67	0.32
IL1RN	13.93	43.39	0.32
CDKN1A	4.79	14.86	0.32
TUBA4A	5.09	15.76	0.32
MAL2	11.51	35.11	0.33
NOTCH3	4.31	13.10	0.33
SMAGP	1.93	5.80	0.33
UGT1A7	6.37	19.01	0.34
THBD	1.70	5.03	0.34
ZNF185	10.98	32.46	0.34
AKR1C3	1.79	5.23	0.34
PDE6D	1.84	5.37	0.34
AP1B1	1.74	5.03	0.35
ICT1	5.56	15.95	0.35
MSMO1	2.36	6.74	0.35
LY6D	30.32	86.32	0.35
MTSS1	2.92	8.18	0.36
DBI	22.14	61.98	0.36
SFN	43.84	122.22	0.36
TIMM8B	2.72	7.56	0.36

ACPP	6.23	17.27	0.36
NDUFS6	3.57	9.70	0.37
SCD	2.85	7.70	0.37
CCND1	31.60	84.93	0.37
KLF8	2.27	6.05	0.38
CCDC56	4.44	11.80	0.38
SERPINB2	2.22	5.85	0.38
ACAA2	4.00	10.53	0.38
RAB25	26.37	69.09	0.38
AKR1B10	4.67	12.22	0.38
S100A16	13.43	34.62	0.39
BDH1	2.90	7.38	0.39
CES2	2.20	5.60	0.39
SLC9A3R1	14.03	34.98	0.40
S100A14	33.90	84.07	0.40
PLD1	2.55	6.31	0.40
TXN	37.76	92.32	0.41
NDUFAF2	2.39	5.81	0.41
DDAH1	2.32	5.65	0.41
ELOVL1	3.19	7.74	0.41
NDUFB3	10.41	25.11	0.41
CYP4B1	2.09	5.03	0.42
RPS26	10.85	26.01	0.42
TPD52L1	5.86	13.95	0.42
EMP1	16.94	39.90	0.42
NDUFS8	2.82	6.59	0.43
C7orf25	2.50	5.85	0.43
NAA20	3.49	8.13	0.43
NDUFAF3	2.19	5.06	0.43
PGD	34.13	78.36	0.44
MTMR10	4.29	9.83	0.44
USMG5	9.76	22.33	0.44
TCEA3	2.63	5.94	0.44
PTGR1	10.10	22.75	0.44
CLDN7	2.87	6.44	0.45
MPZL2	24.32	54.60	0.45
ADRB2	5.44	12.20	0.45
MYCBP	3.62	8.08	0.45
DSP	103.30	230.15	0.45
GLTP	5.09	11.32	0.45
DUOX1	3.92	8.69	0.45
UGDH	8.34	18.44	0.45
CAPNS2	8.34	18.32	0.46

TRIM29	61.30	133.28	0.46
B4GALT5	3.02	6.54	0.46
SUCLG1	5.30	11.48	0.46
ACAT2	3.18	6.88	0.46
GPD1L	4.13	8.92	0.46
ATP5J2	16.09	34.55	0.47
FAM162A	5.24	11.24	0.47
PDCD5	8.34	17.89	0.47
SUCLG2	3.38	7.21	0.47
MGST2	7.99	17.05	0.47
CYCS	7.89	16.76	0.47
TJP1	7.75	16.41	0.47
TAF13	2.55	5.39	0.47
MFSD6	3.32	7.01	0.47
CLTB	3.00	6.32	0.48
NOP10	3.36	7.07	0.48
PKP1	15.18	31.71	0.48
BHLHE40	21.34	44.56	0.48
FAM127A	4.27	8.86	0.48
PPIC	4.65	9.60	0.48
ANXA2	92.29	190.33	0.48
CDH1	7.09	14.58	0.49
MRPS22	5.63	11.42	0.49
DYNLT1	13.59	27.55	0.49
SH3RF2	5.60	11.35	0.49
CHMP4B	4.26	8.61	0.50
CTSC	4.22	8.46	0.50
PGAM1	3.29	6.60	0.50
PRELID1	5.92	11.86	0.50
NDRG2	3.42	6.82	0.50
CIB1	2.53	5.04	0.50
PSMB6	7.10	14.13	0.50
GSTP1	32.41	64.47	0.50
PPL	6.08	12.09	0.50
PGM2	3.38	6.71	0.50
IDH2	3.66	7.23	0.51
MPP7	4.17	8.22	0.51
MRPL20	2.66	5.23	0.51
NDUFB2	17.95	35.20	0.51
JUP	26.38	51.67	0.51
TFRC	4.27	8.32	0.51
DYNLL1	44.34	86.40	0.51
LAD1	8.80	17.11	0.51

GPX2	11.91	23.05	0.52
MIEN1	5.85	11.31	0.52
BRP44L	4.16	8.00	0.52
MRPL13	14.15	27.10	0.52
EZR	11.71	22.32	0.52
UQCRQ	3.56	6.77	0.53
ENSA	29.02	54.95	0.53
OGFRL1	3.79	7.17	0.53
DSG3	45.09	84.90	0.53
CYB5A	3.37	6.34	0.53
FAM206A	3.35	6.28	0.53
SFT2D2	7.11	13.26	0.54
RAB11A	3.74	6.96	0.54
DLG1	3.47	6.45	0.54
MINOS1	3.47	6.44	0.54
MANSC1	3.66	6.79	0.54
FKBP4	7.60	14.08	0.54
NDUFA7	2.87	5.31	0.54
ATP6V0D1	4.76	8.79	0.54
TMSB4X	6.49	11.93	0.54
HSD17B4	8.97	16.43	0.55
PERP	53.86	97.98	0.55
POMP	7.20	13.08	0.55
DHRS1	3.14	5.68	0.55
UBE2T	4.13	7.44	0.56
CAPZB	4.54	8.18	0.56
SLC35A4	2.83	5.09	0.56
ATP5J	23.35	41.95	0.56
ADH7	28.55	50.92	0.56
TMEM154	3.30	5.87	0.56
PTN	22.14	39.28	0.56
C21orf59	7.62	13.51	0.56
ELL2	7.75	13.70	0.57
PSMC2	7.94	14.03	0.57
SUSD4	7.31	12.91	0.57
COX6A1	24.15	42.29	0.57
TIMM21	3.26	5.70	0.57
MDH2	12.11	21.18	0.57
COX8A	10.87	19.00	0.57
ARF6	4.51	7.89	0.57
SLIRP	10.28	17.96	0.57
DAPL1	34.00	59.29	0.57
PPIB	15.23	26.38	0.58

COMT	5.05	8.74	0.58
EMP2	19.88	34.32	0.58
TECR	42.59	73.52	0.58
RAB10	12.17	20.98	0.58
HMGCR	3.78	6.50	0.58
GTF3C6	2.96	5.06	0.58
EIF2B1	6.12	10.43	0.59
ADI1	4.32	7.34	0.59
OSTF1	3.95	6.69	0.59
ERMP1	3.58	6.06	0.59
JAG1	4.69	7.93	0.59
SERPINB5	13.09	22.01	0.59
CMAS	5.80	9.75	0.60
ARPC3	6.57	10.99	0.60
MOCS2	3.93	6.58	0.60
NDUFS1	5.03	8.36	0.60
RAB5C	3.10	5.14	0.60
RFFL	3.06	5.08	0.60
PSMD14	4.19	6.89	0.61
DNAJC8	4.39	7.18	0.61
ALDH9A1	12.85	20.98	0.61
AIM1	11.61	18.73	0.62
EPHX1	9.07	14.55	0.62
CA12	7.79	12.44	0.63
TM4SF1	5.47	8.70	0.63
MTX2	5.08	8.07	0.63
LMAN2	4.18	6.62	0.63
SLC25A5	28.68	45.13	0.64
YWHAZ	90.00	140.48	0.64
MRPS10	7.45	11.58	0.64
ATP5H	12.00	18.55	0.65
TIMM23	7.04	10.80	0.65
CLCA2	20.43	31.24	0.65
HNRNPAB	20.73	30.72	0.67
CAPZA1	8.34	12.33	0.68
ANXA7	11.41	16.81	0.68
TOMM70A	4.05	5.87	0.69
MT1X	30.03	43.49	0.69

* the green font denotes esophagus-specific genes
